# Sexual Selection Halts the Relaxation of Protamine 2 among Rodents

**DOI:** 10.1371/journal.pone.0029247

**Published:** 2011-12-21

**Authors:** Lena Lüke, Alberto Vicens, Francois Serra, Juan Jose Luque-Larena, Hernán Dopazo, Eduardo R. S. Roldan, Montserrat Gomendio

**Affiliations:** 1 Reproductive Ecology and Biology Group, Museo Nacional de Ciencias Naturales (CSIC), Madrid, Spain; 2 Evolutionary Genomics Laboratory, Bioinformatics and Genomics Department, Centro de Investigación Príncipe Felipe (CIPF), Valencia, Spain; 3 Area de Zoología, Departamento de Ciencias Agroforestales, E.T.S. Ingenierías Agrarias, Universidad de Valladolid, Campus La Yutera, Palencia, Spain; Institut Jacques Monod, France

## Abstract

Sexual selection has been proposed as the driving force promoting the rapid evolutionary changes observed in some reproductive genes including protamines. We test this hypothesis in a group of rodents which show marked differences in the intensity of sexual selection. Levels of sperm competition were not associated with the evolutionary rates of protamine 1 but, contrary to expectations, were negatively related to the evolutionary rate of cleaved- and mature-protamine 2. Since both domains were found to be under relaxation, our findings reveal an unforeseen role of sexual selection: to halt the degree of degeneration that proteins within families may experience due to functional redundancy. The degree of relaxation of protamine 2 in this group of rodents is such that in some species it has become dysfunctional and it is not expressed in mature spermatozoa. In contrast, protamine 1 is functionally conserved but shows directed positive selection on specific sites which are functionally relevant such as DNA-anchoring domains and phosphorylation sites. We conclude that in rodents protamine 2 is under relaxation and that sexual selection removes deleterious mutations among species with high levels of sperm competition to maintain the protein functional and the spermatozoa competitive.

## Introduction

The idea that genes involved in reproductive processes evolve rapidly has gained widespread acceptance [1], [2]. However, recent evidence suggests that the evolutionary pattern of reproduction-related proteins is more heterogeneous than previously assumed [3]. Rates of molecular evolution vary according to different components of the reproductive process, different timing of gene expression, and the tissue or organ in which genes are expressed [4]–[7]. Heterogeneity in evolutionary rates is even found in mature sperm cell proteins, so that only a small proportion evolves rapidly [8]. Thus, most reproductive proteins seem to be under strong evolutionary constraints and only a small subset seems to evolve rapidly.

Since sexual selection is known to drive the rapid evolution of reproductive traits which make sperm more competitive, it has been proposed that it could also drive rapid evolution among genes related to such processes [1], [2], [3]. Sperm competition occurs when females mate with more than one male, and ejaculates from rival males compete within the female tract to fertilise the available ova [9]. An almost universal response to increased levels of sperm competition is an increase in sperm numbers, which is achieved by an increase in relative testes size [9], [10]. Relative testes size has been found to be associated to levels of sperm competition in many taxa [9]–[11], has been shown to be related to levels of genetic paternity [12], and it is available for a large number of species, so it is widely used as a reliable index of levels of sperm competition. Sperm competition also favours improvements in sperm quality [13], increases in size and swimming speed [14]–[16], and sperm with elongated heads that offer a lower degree of resistance to the medium in which they swim (less drag) [16]. Finally, sperm competition selects a larger proportion of sperm ready to fertilise and sperm which are more sensitive to the signals emitted by the ovum [17].

It has been widely assumed that such changes in phenotypic traits would be linked to rapid divergence in the coding sequences of proteins underlying such reproductive processes [1], [2]. However, despite the popularity of this hypothesis the evidence is extremely limited. Only three studies have found positive associations between rates of divergence in coding sequences of reproductive genes and levels of sperm competition: two genes coding for proteins in the seminal fluid related to coagulum or copulatory plug formation (SEMG2 [18], SVS2 [19]), and proteins expressed in the sperm surface (ADAMs 2 and 18 [20]). A recent study has found the opposite pattern, i.e. more rapid evolution among seminal fluid proteins in monandrous taxa, leading to the suggestion that relaxed selective constraint may also be responsible for fast evolutionary rates [21].

It has been claimed that protamines are the fastest evolving reproductive proteins and that this is a consequence of sexual selection acting on them [22]. Evidence of positive selection has been detected for protamine 1 in primates [22], [23]. However, recent studies on protamines have revealed a different scenario. A study on a group of closely related species of *Mus* (Rodentia) found links between the degree of divergence in the promoters of protamine 2, levels of sperm competition, and sperm swimming velocity [24]. This study concluded that in the early stages of speciation only weak positive selection is detected, while major changes occur in promoters, which increase the efficiency of DNA condensation within the sperm head, leading to changes in sperm head shape which presumably make it more hydrodynamic resulting in an increase in sperm swimming speed [24].

Protamines are a diverse family of small, arginine-rich nuclear proteins that replace histones and transition proteins during the process of sperm nucleus condensation in spermatogenesis [25]. The high charge density of these arginine-rich proteins allows them to bind DNA with high affinity and to more efficiently shield the charges on the DNA phosphate backbone than histones. Such charge neutralization results in a genetically inactive state of the spermatid genome and in a major reduction of the size of the sperm nucleus [26].

Expression of protamines is specific to testis and protamine mRNAs are exclusively detected in postmeiotic spermatid stages [27], [28]. Protamines first appear in elongating spermatids, coincident with the initiation of the final stage of chromatin condensation. In some eutherian mammals two types of protamines have been identified: protamine 1 and protamine 2. While protamine 1 is a major sperm protamine in all mammals, protamine 2 is present only in the sperm of primates, most rodents, and a subset of other placental mammals [27], [28].

Protamine 1 is typically 49 or 50 amino acids long and contains a highly conserved arginine rich DNA-binding domain as well as multiple serine and threonine residues that may be used as phosphorylation sites [27]. Protamine 1 is not synthesized as precursor. In contrast, the protamine 2 gene codes for a protamine 2 precursor (hereafter, “protamine 2 precursor”), which is processed in late-spermatids by proteolytic cleavages in its N-terminal region resulting in about 40% of the protein being removed (this removed domain is hereafter referred to as “cleaved-protamine 2”) [25], [28]. The fully processed form of protamine 2 (herafter, “mature-protamine 2”) is slightly larger than protamine 1 and has 63 amino acids in the mouse. Mature-protamine 2 exhibits similar structural and functional properties to protamine 1, but this is not the case for cleaved-protamine 2.

The aim of this study is to analyse the evolution of the coding sequences of protamines 1 and 2 in a group of 16 rodent species, belonging to the family Cricetidae (subfamilies Arvicolinae and Cricetinae). These species represent a more divergent group, which split from other members of the Cricetidae about 18 MYA [29], than the group of *Mus* species examined in a previous study (Fig. S1) [24], which emerged about 5 MYA [30]. We therefore expected to find more changes in the protamine coding sequences in this group of more divergent taxa. These species also show a wider range of levels of sperm competition [13]. We tested if sexual selection is influencing the rate of molecular evolution of protamines. Since the protamine 2 precursor consists of two structurally and functionally different parts, namely cleaved-protamine 2 and mature-protamine 2, we analyse them separately to investigate the possibility that they may be under different selective regimes. Analysing cleaved- and mature-protamine 2 separately also allows us to compare protamine 1 with its functional equivalent, i.e. mature-protamine 2. In order to improve our understanding of the adaptive meaning of changes at the molecular level, we carried out a specific analysis of changes at functionally important sites such as DNA-anchoring domains and phosphorylation sites.

## Materials and Methods

### Species

The study includes 16 species of the family Cricetidae, 10 of which belong to the subfamily Arvicolinae (*Arvicola sapidus*, *Arvicola terrestris*, *Clethrionomys glareolus*, *Chinomys nivalis*, *Microtus arvalis*, *Microtus cabrerae*, *Microtus agrestis*, *Microtus gerbei*, *Pitymys duodecimostatus*, *Pitymys lusitanicus*), 5 to the subfamily Cricetinae (*Cricetulus griseus*, *Mesocricetus auratus*, *Phodopus sungorus*, *Phodopus campbelli*, *Phodopus roborovskii*), and 1 to the subfamily Sigmodontinae *(Sigmodon hispidus)* [29]. This group of species has experienced rapid evolutionary radiation and diversification [29], and has different levels of sperm competition (as shown by their differences in relative testes size-see below). Individuals belonging to the Arvicolinae were trapped in the field during the breeding season at different locations in Spain [13]. Individuals belonging to the Cricetinae were from laboratory strains purchased from commercial suppliers and were unrelated. We obtained the gene sequences of at least 4 individuals per species to generate a consensus sequence.

### Protamine sequences

Protamine 1 *(Prm1)* and protamine 2 (*Prm2*) sequences of *Sigmodon hispidus* were obtained from NCBI Genebank (Accession EU980395 for *Prm1* and EU980396 for *Prm2*). *Prm1* sequence for *Phodopus sungorus*, *Phodopus roborovskii*, and *Cricetulus griseus*, and *Prm1* and *Prm2* sequences for *Mesocricetus auratus* were obtained from the literature [19], [31]. All other nucleotide sequences were obtained through PCR amplification and sequencing.

### DNA isolation and gene amplification

Genomic DNA was extracted from different frozen tissues using the E.Z.N.A® Tissue DNA kit (Omega, Madrid, Spain) following the manufacturer's recommendations.

Protamine sequences were amplified by Polymerase Chain Reaction (PCR). PCR mixtures were prepared in a 50 µl volume containing PCR Gold buffer 1× (Roche, Barcelona, Spain), 2.5 mM MgCl_2_ (Roche), 0.8 mM dNTPs mix supplying 0.2 mM of each deoxinucleotide triphosphate (Applied-Biosystems, Barcelona, Spain), 0.3 mM of forward and reverse primers (Applied Biosystems), 2 U of Taq Gold DNA polymerase (Roche), and 20–100 ng/µl of genomic DNA template. All PCRs were performed in a Veriti thermocycler (Applied-Biosystems). The conditions of the thermocycler program consisted of 35–45 cycles with an initial denaturation of 95°C for 30–40 s, an annealing stage at 52–62°C (depending on template and primers) for 40 s, and an elongation stage at 72°C for 30–50 s (depending on gene length).

PCR primers were designed on the basis of protamine genomic sequences of other closely related rodent species accessible in the literature or in NCBI GeneBank. All alignments were performed in Bioedit [32] and most conserved segments within untranslated regions (UTRs) were chosen. When protamines of one or more individuals of each closely related group were sequenced, new specific primers on the basis of these sequences were designed to ensure efficient PCR performance.

PCR products were purified by using the E.Z.N.A.® Cycle Pure kit (Omega). In cases in which additional nonspecific bands were obtained after separation in a 1.5% agarose gel, bands of <600 bp size for *Prm2* and <300 bp size for *Prm1* were extracted with E.Z.N.A.® Gel Extraction Kit (Omega). Purified products were sequenced (Secugen S.L., Madrid, Spain).

### Alignments and Trees

The processing of the sequenced fragments was done using the sequence viewer and alignment editor Bioedit [32]. The fragments were reduced to a consensus sequence and trimmed to coding sequence. These sequences combined with database sequences were aligned on the basis of their amino acid sequences and retranslated using ClustalW implemented in Bioedit [32]. We produced two different input trees. The input tree used for branch and site analyses includes our chosen range of Cricetidae with *Mus musculus* used as outgroup (Fig. S1A). In the clade analysis *Oryctolagus cuniculus* was included as outgroup and 12 Muroidea species were used as a background (Fig. S1B).

The phylogenetic trees were built based on information gathered from the literature [13], [24], [33]–[35] (Fig. S1).

### Selective Pressures

The nonsynonymous/synonymous substitutions rate ratio (ω = d_N_/d_S_) is an indicator of selective pressure at the protein level, with ω = 1 indicating neutral evolution, ω<1 purifying selection, and ω>1 diversifying positive selection [36].

To estimate rates of sequence evolution we used the application Codeml implemented in PAML 4 [37], [38] through the ETE toolkit [39]. Based on the input alignments and the input tree the ω-value (d_N_/d_S_) was generated by different models for all/chosen branches of the tree (branch analysis) or for each codon site of the alignment (site analysis). To test for positive selection on branches or sites, the models (see below) are compared with corresponding null-models by means of likelihood-ratio-tests. These tests compare twice the log-likelihoods of the used model (alternative) and the null-model (conservative) to critical values from a chi-square distribution with the degrees of freedom equal to the difference in the number of parameters between the two models. The positive selection on sites was additionally crosschecked through the program SLR [40]. For the Codeml codon frequency setting, as well as the setting for number of categories, we used the setting with the best fit for each analysis according to the preliminary likelihood-ratio-analysis.

### Clade analysis

By computing the clade model comparing Cricetidae as foreground clade against a background of 12 murid species (available from NCBI GeneBank) we obtained the evolutionary rate of the foreground clade in contrast to the background (Fig. S1B). Three models were computed: Model 1 “one ratio” in which all taxa were constrained to evolve at the same rate; Model 2 “two-ratio Cricetidae fixed” where the background clade ω was let free to be estimated and the Cricetidae clade ω was restrained to a value of ω = 1; and Model 3 “two ratio” model which estimates for both background and the Cricetidae clade a free and independent ω.

True cases of positive selection for Cricetidae are reported if three conditions are fulfilled: Cricetidae clade and background clade evolve at a significantly different rates (Model 1 *vs* Model 3), Model 3 presents a better fit against the Model 2, and the ω value estimated is higher than 1. Candidates for relaxation for Cricetidae are reported if two conditions are fulfilled: Cricetidae clade evolves at a significantly different omega than the background (Model 1 *vs* Model 3), and this omega was not significantly different from 1 (Model 2 *vs* Model 3) [41]. Comparisons between models were done by means of likelihood-ratio tests (Fig. S2).

### Evolutionary rates and levels of sperm competition

To obtain species-specific ω values to analyse the relation between evolutionary rate and sperm competition levels for each species, we used the free branch model (PAML 4, Codeml) and calculated an ω value for each species by addition of d_N_ values and d_S_ values from the root to the terminal species branch and taking the ratio (d_N_/d_S_) of the sum to obtain the ω value. By calculating ω in this way we take into account the total accumulated selective pressures in protamines during their evolution, which is more suitable for testing relationships against phenotypic data which do reflect the whole phenotypic evolution [42].

We used the species relative testes mass as a proxy for levels of sperm competition as in our previous studies [13], [16]. This earlier work has shown that relative testes mass is related to a wide range of ejaculate traits which improve sperm competitiveness, and is therefore a reliable indicator of the intensity of post-copulatory sexual selection. Males (N = 5 for each species) were sacrificed by cervical dislocation and weighed. After removal the testes where weighed and relative testes mass was then calculated using the regression equation for rodents in Kenagy and Trombulak [43]. Animal handling and housing followed the standards of the Spanish Animal Protection Regulation RD1201/2005, which conforms to European Union Regulation 2003/65. This study was approved by the Bioethics Committee of the Consejo Superior de Investigaciones Científicas (CSIC, Spain).

### Phylogenetic generalized least squares (pGLS) analysis

Species data may not be free of phylogenetic association, since they may share character values as a result of a common ancestry rather than independent evolution, and thus may not be truly independent. To control for this phylogenetic inertia, we used phylogenetic generalized least squares (pGLS) analyses between the ω-values computed from the root and the species relative testes mass. We performed the pGLS analysis using the program COMPARE 4.6b [44].

### Site analysis

To test evolution along coding sequences and infer amino acids under positive selection we applied likelihood ratio tests comparing a null model that does not allow sites with ω>1 with a selection model that does. We used two likelihood ratio tests. The first compared a nearly neutral model M1a, which assumes values for ω between 0 and 1, with a model M2a which allows values of ω>1. The second test is more refined and compares two models assuming a β distribution for ω values. In this case, the null model M7 that limits ω between 0 and 1 is compared to the alternative model M8, that adds an extra class of sites with an ω ratio estimated that can be greater than 1 [40], [45]. If the alternative models showed a significantly better fit in the likelihood-ratio-test Bayesian statistics were used to identify those codons that have been subject to adaptive evolution, where posterior probabilities higher than 0.95 in both M2a and M8 were considered.

### Anchoring domains, post-translational modification motifs and cleaving sites

Possible target areas of post-translational modifications were determined using the ScanProSite tool of ExPASy Proteomic Server and verified by Net-Phos server 2.0. Proposed phosphorylation sites in the literature [27], [46] were also taken into account.

DNA-anchoring domains were predicted selecting regions containing 3 or more consecutive arginine or lysine residues flanked by short peptide segments containing cysteine residues, following the literature on structural and functional characteristics of protamines [27], [47].

Post-translational processing cleavage sites in the protamine 2 precursor were identified based on previous studies [48].

## Results

### Degree of similarity between protamines 1 and 2

A pairwise percentage identity analysis of the amino acid sequences of *Mus musculus* protamines and their domains using the sequence alignment ClustalW implemented in Bioedit [32] shows the following amino acid sequence identities: PRM1 and mature-PRM2, 50.9%; PRM1 and cleaved-PRM2, 16%; and cleaved-PRM2 and mature-PRM2 15%.

NCBI BLAST (Tool: blastn) [49] searches indicate no sequence throughout the mouse genome that shows a significant similarity to cleaved-PRM2 over its whole length. However, it seems to include a repetitive element (5′-AGGAGCAGGGGCAGGGGCAAGGGCTGAG-3′) approximately between bases 70 and 100. This element was found in BLAST searches throughout the mouse genome (e.g. *Mus musculus* chromosome 11 genomic contig, strain C57BL/6J: max score = 44.6, e-value = 0.02) as well as in virus genomes (e.g. Ictalurid herpesvirus: max score = 81.9, e-value = 0.07).

On the basis of the structural and functional similarities between PRM1 and mature-PRM2, as well as the fact that cleaved-PRM2 shows few similarities to either of them and seems to have a different origin, subsequent analyses were carried out on cleaved-PRM2 and mature-PRM2 separately.

### Clade analysis

In the evolutionary analysis of the Cricetidae clade, in contrast to other rodents, the likelihood-ratio-test comparing the two ratio model and the one ratio model in codeml (PAML 4) suggests a significant difference in the selective constraints of the Cricetidae clade and the rodent background for both *Prm1* and the two *Prm2* domains (Table 1). The likelihood-ratio-test comparison between the Model 2 “two ratio, Cricetidae fixed” and Model 3 “two ratio” suggests positive selection for the Cricetidae clade for *Prm1*, while mature-*Prm2* and cleaved-*Prm2* show evidence of relaxation (Table 1).

**Table 1:** Clade analysis for protamines.

		Protamine 1		cleaved-Protamine 2		mature-Protamine 2	
Foreground clade	Background clade	ω	Selection	ω	Selection	ω	Selection
Cricetidae	Muroidea	2.44	Positive	2.75	Relaxation	1.66	Relaxation

Clade analysis of a Cricetidae foreground clade against a murid background.

### Evolutionary rate of divergence and relative testes mass

**Figure 1:**
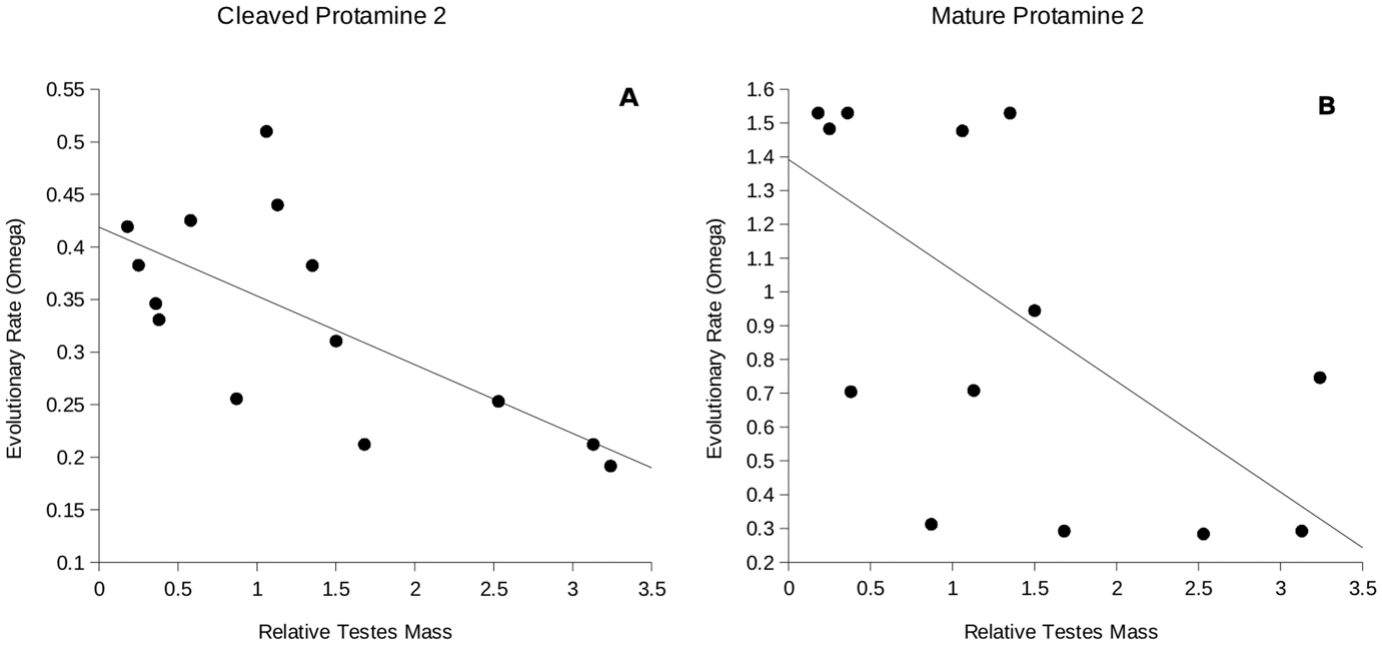
Evolutionary rate of protamine 2 evolution and sexual selection in rodents. Relationship between evolutionary rate (ω) and relative testes mass for **A** cleaved region of protamine 2, and **B** mature-protamine 2.

To test for a relationship between the evolutionary rates of protamines and levels of sexual selection we correlated the ω value with the species-specific relative testes mass. To correct for phylogenetic effects we used the pGLS tool of the program COMPARE 4.6b [44]. ω values of *Prm1* showed no significant relationship with relative testes mass although there is a weak negative relationship (α = 1.42, CI 95% (slope) = −0.18 to 0.08, correlation = −0.194) (Table 2). In contrast, ω values of mature-*Prm2* and cleaved-*Prm2* showed significant negative relationships with relative testes mass (cleaved-*Prm2*: α = 15.50, CI 95% (slope) = −11.54 to −2.40, correlation = −0.69; mature-*Prm2*: α = 8.17, CI 95% (slope) = −1.98 to −0.21, correlation = −0.574) (Fig. 1, Table 2).

**Table 2:** Protamine evolution and relative testis mass.

		95% CI (slope)				
Set	N	CI−	CI+	lnL	alpha	correlation
Protamine 1	15	−0.18	0.08	−14.951	1.42	−0.194
cleaved-Protamine 2	14	−11.54	−2.95	−2.40	15.5	−0.69*
mature-Protamine 2	14	−1.98	−0.21	−3.59	8.17	−0.574*

PGLS analysis results. N indicates number of taxa analyzed, CI− and CI+ the confidence intervals for the regression slope, lnL the maximum likelihood estimate of alpha, and alpha the measure of evolutionary constraints acting on phenotypes (COMPARE 4.6b). Asterisks indicate statistically significant relationships.

### Site analysis

To test selective pressures influencing protamine sequences at the site level, we set a Bayesian Empirical Bayes (BEB) that classifies all sites in three classes in terms of its posterior mean ω. The class differentiation is computed by model 2a or 1a depending on which model shows the best fit (likelihood-ratio test). Class 1 contains sites with ω values between 0 and 1 and therefore subject to purifying selection. Class contains 2 sites evolving neutrally with an ω value close to 1. In class 3 (just in model 2a) sites with ω greater than 1 are indicative of positive selection or functional relaxation. The percentage of sites in each class is shown in Table 3. In PRM1 most sites are evolving neutrally (site class 2)(51%) or are under purifying selection (site class 1) (41%) (Fig. S3). The selection models (M2a and M8) explained the evolutionary influences on PRM1 significantly better (P<0.01 for M2 and P<0.05 for M8) than the neutral models (M1a and M7). Three positively selected sites (33C, 38T, 39V) were detected by model M2a and two sites (33C, 39V) by the more stringent model M8 (Table 3). In the case of cleaved-PRM2 it was not significantly better explained by the selection models. The best fit was shown by the neutral models. In addition, according to the site class distribution 63.02% of sites are under purifying selection, and 36.98% are evolving neutrally (Fig. S4) with 10 sites under significant relaxation. In contrast, mature-PRM2 is under lower selective constraints, with only 13.32% of sites under purifying selection and 86.67% belonging to site class 2 with ω values close to one, although only 14 sites are under significant relaxation (Fig. S5). The selection models were not accepted in the likelihood-ratio-test. The SLR analysis supported these results. Codeml (PAML 4) output files for the selection model M2 have been included in the supplementary material (Figs. S6–8).

**Table 3:** Site analysis. Selective pressures and Likelihood-ratio-tests in protamine 1 and protamine 2 sequences.

Protein (domain)	N	Lc	Selective pressures:	% class 1 (ω<1)	% class 2 (ω = 1)	% class 3 (ω>1)	Best fit model	2Δl	parameter estimates	PSS
			All sites	41	51	8	M2a (selection)	9.71**	p_0_ = 0.41, p_1_ = 0.50993, p_2_ = 0.07950, ω_0_ = 069,	33C*, 39V
**Protamine1**	16	153	Anchoring domains	70	16.6	13.3	M8 (beta and ω)	7.34*	p_0_ = 0.58495, p = 0.0050, q = 0.04910, p_1_ = 0.41505, ω = 2.65844	33C*, 38T*, 39V**
			Phosphorylation sites	57.1	28.6	14.3				
			All sites	63.02	36.98		M1a (neutral)	2.64	p_0_ = 0.63016, p_1_ = 0.36984, ω_1_ = 1.00	not allowed
**cleaved-Protamine 2**	16	165	Cleaving sites	57.14	42.86		M7 (beta)	3.57	p = 0.09398, q = 0.13753	not allowed
			Phosphorylation sites	100	0					
			All sites	13.32	86.67		M1a (neutral)	2.12	p_0_ = 0.13325, p_1_ = 0.86675, ω_1_ = 1.00	not allowed
**mature-Protamine 2**	16	162	Anchoring domains	0	100		M7 (beta)	2.52	p = 35.58178, q = 0.00500	not allowed
			Phosphorylation sites	0	100					

Parameter estimation and likelihood scores under models of variable ω ratios among sites for protamine 1, cleaved region of protamine 2 and the mature form of protamine 2. The data have N sequences, each of Lc codons after alignment gaps are removed. Differences between log-likelihood values of models with 99% statistical significance level for 2 d. f. are indicated in ** and with 95% of statistical significance in *. The proportion of sites under positive selection (p1), or under selective constraint (p0) and parameters p and q for the beta distribution are given. Positively selected sites (PSS) with a posterior probability >0.95 (*) and >0.99 (**) in a Bayes Empirical Bayes are indicated. Selectives pressures are shown as proportion of amino acid sites for different classes of selective regimes. Class 1: sites under purifying selection (0<ω<1); Class 2: sites neutrally evolving (ω = 1); Class 3: sites subject to positive selection or relaxation (ω>1). Proportions of sites in each selective class were also calculated for DNA-anchoring domains, phosphorylation motifs and cleaving sites.

### Evolutionary profile of functional domains

**Figure 2:**
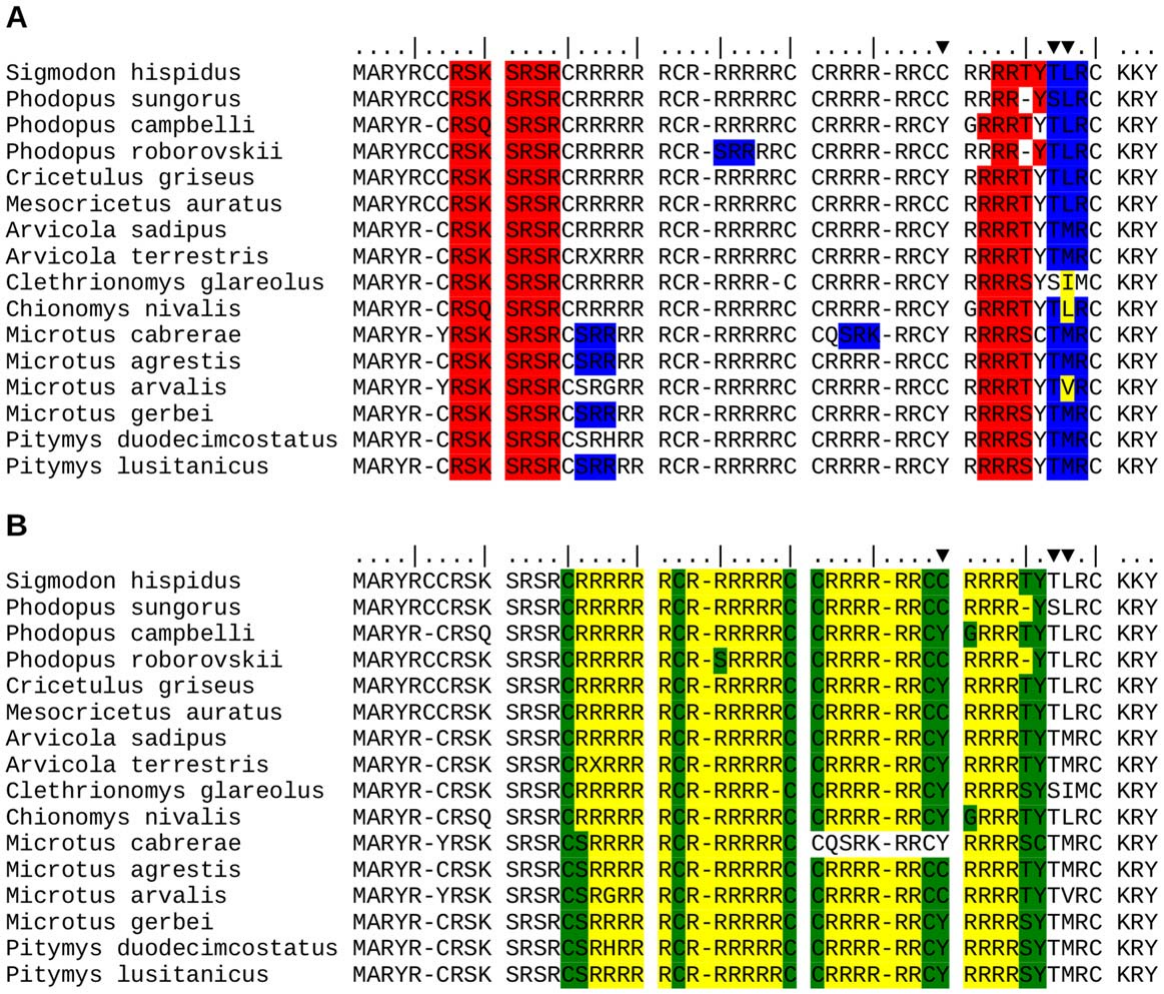
Phosphorylation sites and anchoring domains in protamine 1. **A** - Alignment of Cricetidae species indicating predicted phosphorylation sites. Highlighted in blue: Phosphorylation motifs by Protein Kinase C (PKC) according to ScanProSite and NetPhos. Highlighted in red: Phosphorylation motifs by Protein Kinase A (PKA) according ScanProSite and NetPhos. Arrows indicate positively selected sites (Codeml M2a/M8). **B** - Alignment of Cricetidae species indicating DNA-anchoring domains. Highlighted in green: Arg and Lys residues directly implicated in DNA binding. Highlighted in yellow: Flanking uncharged residues that stabilize DNA-Arg/Lys interactions. Arrows indicate positively selected sites (Codeml M2a/M8).

**Figure 3:**
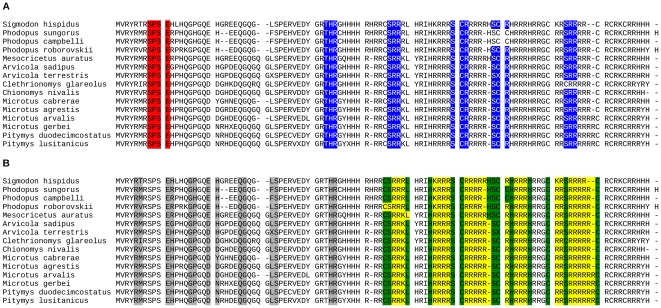
Phosphorylation sites, anchoring domains and cleaving sites in protamine 2. **A** - Alignment of Cricetidae species indicating predicted phosphorylation sites. Highlighted in blue: Phosphorylation motifs by Protein Kinase C (PKC) according to ScanProSite and NetPhos. Highlighted in red: Phosphorylation motifs by caseine kinase II according ScanProSite and NetPhos. **B** - Alignment of Cricetidae species indicating DNA-anchoring domains and cleaving sites. Highlighted in green: Arg and Lys residues directly implicated in DNA binding. Marked in yellow: Flanking uncharged residues that stabilize DNA-Arg/Lys interactions. Highlighted in grey: Cleaving sites.

The two protamines contain several putative DNA-anchoring domains composed of three or more consecutive arginine and lysine residues [27]. For PRM1, most sites involved in DNA-anchoring domains are subjected to purifying selection with low ω values (70% of sites of class 1 after BEB analysis), but there is also a substantial proportion falling into site class 3 (positive selection) (13.3%) (Table 3, Fig. S3). Moreover, one out of three positively selected sites (33C) falls into the flanking region of a DNA-anchoring domain (Figs. 2 and S3). DNA-anchoring domains in mature-PRM2 seem to be evolving neutrally with 100% of sites showing ω values close to 1 (class 2) (Table 3, Fig. 3).

Both protamines undergo post-translational modifications through phosphorylation of several motifs containing typically serine and threonine residues. This could regulate the interaction with DNA [27]. In PRM1, 57.1% of sites within phosphorylation motifs were found under constraints of purifying selection after BEB analysis (Table 3). The rest of the residues were evolving neutrally (28.6% class 2) and a substantial proportion under positive selection (14.3% class 3). Furthermore, two out of three sites under positive selection (38T and 39V) are located within phosphorylation sites, specifically in phosphorylation targets for protein kinase C, and one affecting directly a threonine/serine residue (Fig. 2).

Phosphorylation sites in cleaved-PRM2 seem to evolve under high selective constraints showing 100% of sites with a low ω value (class 1). Moreover, evidence of positive selection was not found for any site (Table 3). In the case of mature-PRM2, all sites involved in post-translational phosphorylation showed ω values close to 1 (class 2). Thus, it can be assumed that phosphorylation sites in mature-PRM2 are evolving neutrally, following the evolutionary trend of the whole sequence (Figs. 3 and S4–5).

Precursor-PRM2 is processed after translation by several specific cleaving sites distributed along the cleaved-PRM2 region by a proteolytic process [27]. A total of 8 out of 14 cleaving sites in cleaved-PRM2 were estimated to be under selective constraints (57.14% of sites in class 1) and the rest of the sites were evolving neutrally with ω values close to 1 (42.86%) (Table 3).

### Protamine 2 pseudogene in *Cricetulus griseus*

**Figure 4:**
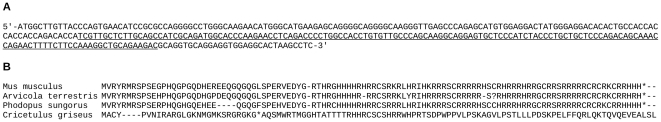
Protamine 2 of *Cricetulus griseus*. **A** - Coding sequence of the protamine 2 gene of *C. griseus*. Underlined is a region with null homology to any protamine sequence. **B** - Comparison between protamine 2 protein sequences of *Mus musculus*, *A. terrestris*, *P. sungorus* and *C. griseus*.

We compared the *Prm2* sequence of *Cricetulus griseus* with that of *P. sungorus*, *A. terrestris* and *M. musculus*, representing Cricetidae and non-Cricetidae respectively. The coding sequence of *C. griseus Prm2* reveals a complete divergence in relation to its orthologue, suggesting that *Prm2* in this species has accumulated successive and defective mutations through evolution thus becoming a dysfunctional gene or pseudogene (Fig. 4). *Cricetulus griseus* was therefore excluded from *Prm2* analyses due to its degeneration and its null expression in this species [31].

## Discussion

The results of this study show that, among rodents, protamine 1 and the two domains of protamine 2 evolve under different selective regimes, and are influenced by sexual selection to different extents. Clade tests show that protamine 1 is under positive selection, but no relationship between the rate of divergence and relative testes size (a proxy for levels of sperm competition) was found. In contrast, cleaved-protamine 2 and mature-protamine 2 are under relaxation, and there is a negative relationship for both between rate of divergence and relative testes size, suggesting a strong influence of sperm competition.

Previous studies on the influence of sexual selection on the evolution of protamines found only weak evidence of positive selection on the protamine 2 precursor in a group of closely related species of *Mus* [24], but a high degree of divergence in promoters which was associated with levels of sperm competition and sperm function. The latter study concluded that in the early stages of speciation few changes in coding sequences are found, whereas further changes are likely to be present between species that have diverged to a greater extent. The results presented here support this claim, since we find evidence of positive selection and relaxation in a group of more distantly related species of rodents.

It is generally assumed that post-copulatory sexual selection promotes positive selection by driving rapid changes in reproductive genes which result in adaptive advantages [1]–[3], although it has also been shown that seminal fluid proteins show faster evolutionary rates among monandrous species due to relaxation of selective constraints [21]. No association was found between levels of sperm competition and rate of divergence in protamine 1 in this and previous studies [19], suggesting that positive selection in this protein is not driven by sexual selection. In contrast, a negative relationship was found between rate of divergence of both cleaved- and mature-protamine 2 and levels of sperm competition, in agreement with studies on seminal fluid proteins in buttlerflies [21]. Since both domains are under relaxation, this suggests that the role of sexual selection in this case is not to promote rapid changes, but rather to halt the degree of relaxation of protamine 2 by removing deleterious mutations. Since protamines 1 and 2 are very similar and seem to perform the same function, it is possible that protamine 2 is under relaxation due to functional redundancy. In this scenario, the role of sexual selection may be to remove deleterious mutations which diminish sperm competitiveness among species with high levels of sperm competition, so it will act as a force “opposing” further degeneration in order the maintain the protein fully functional.

It is noteworthy that sexual selection has only been shown to influence proteins belonging to families such as SEMG2 [18], SVS 2 [19] and ADAMs 2 and 18 [20], and protamine 2 (this study). This evidence suggests that the effects of sexual selection may be particularly pronounced among families of proteins, which share many functional similarities and may play complementary roles. In this scenario, it is possible that the protein which performs the main function will remain conserved, while other proteins of the same family may be “free” either to change rapidly under the influence of sexual selection to evolve competitive traits, or may be under relaxation due to functional redundancy and in this latter case sexual selection prevents deterioration beyond a certain level in order to preserve its functionality.

The idea that protamine 2 in this group of rodents may suffer from relaxation of selective constraints and degeneration is supported by the fact that one of the species studied (*Cricetulus griseus*) has a protamine 2 sequence which is highly divergent and mature-protamine 2 is not expressed in sperm. Similarly, other species of this family (Cricetidae) do not present mature-protamine 2 in sperm [31], [50]. Thus, among hamsters, species belonging to the genera *Cricetulus* and *Cricetus*, do not have mature-protamine 2 in fully-differentiated spermatozoa, while those belonging to the genera *Mesocricetus* and *Phodopus* do. Furthermore, among the latter species the proportion of mature-protamine 2 in sperm ranges between 33 and 50%, which is lower than the proportion found in other rodents such as *Mus* [50]. This evidence suggests that protamine 2 in *Cricetulus griseus* has become a dysfunctional gene or a pseudogene, supporting the idea that the role of protamine 2 is secondary in this group and that protamine 1, which is present in all species, performs the main function of DNA compaction.

Interestingly, all mammalian species have protamine 1 but protamine 2 is present only in primates and some rodents [27], [28], and among the latter the proportion of protamine 2 in mature sperm varies widely between species [31], [50], but the significance of this interspecific level of variation is not well understood. The high degree of similarity between protamine 1 and mature-protamine 2 supports the idea that they may share a common origin [51]. In contrast, the region of protamine 2 that is cleaved shows a very low degree of similarity with protamine 1, and may be of retroviral origin [51].

Site analysis revealed that protamine 1 is evolving under selective regimes, while the two domains of protamine 2 are evolving neutrally. For protamine 1, we detected robust evidence of positive selection acting on two sites (positions 33 and 39) and weaker evidence for one additional site (position 38), in agreement with previous findings on rodents [19]. Strong evidence of positive selection in protamine 1 was also detected comparing human and mouse sperm-specific orthologues [52], but was not found when closely related species were analysed [24]. Our data show that while most sites are under purifying selection or evolving neutrally, which helps to maintain a conserved structure and ensures that the protein remains functional, selection targets a few sites which may be of particular functional relevance.

In contrast, cleaved-protamine 2 and mature-protamine 2 seem to evolve neutrally. Among cleaved-protamine 2 most sites are under purifying selection, while among mature-protamine 2 most sites are evolving neutrally. No positively selected sites were detected. So far, few studies have analysed the evolution of protamine 2 and they have analyzed the entire precursor protein without distinguishing its two regions [24], [52]. Torgerson et al. [52] did not find evidence of positive selection driving the evolution of the protamine 2 precursor comparing human and mouse orthologues, thus supporting our results. On the other hand, a study of a group of closely related species of *Mus* found evidence of one site under positive selection [24]. This site in our analysis was not detected as positively selected but showed a high ω value.

Among protamines, functionally important sites include DNA-anchoring domains, phosporylation sites and cleaving sites. DNA-anchoring domains are only present in protamine 1 and mature-protamine 2, phosphorylation sites are present in both protamines, and cleaving sites are by definition only present in the section of protamine 2 which is sequentially cleaved. Most DNA-anchoring domains are under purifying selection in protamine 1, although a substantial proportion are under positive selection. In contrast, all DNA-anchoring domains are evolving neutrally in mature-protamine 2. Similary, most phosphorylation sites are under purifying selection in protamine 1, although a susbtantial proportion are under positive selection. Among cleaved-protamine 2 all phosporylation motifs are under purifying selection, while all of them evolve neutrally in mature-protamine 2. It is important to point out that two positively selected sites detected in protamine 1 (38T, 39V) are located in phosphorylation motifs, while one (33C) is located in the flanking region of a DNA-anchoring domain, suggesting that they are the targets of selection. The functional importance of DNA-anchoring domains is obvious, while that of phosphorylation sites is currently less clear. Protamines are phosphorylated as soon as they are synthesized and phosphorylation is required for protamines to bind to DNA [27], [28], so it is possible that positive selection on these sites may also result in changes in the degree of condensation of DNA. Finally, 57% of cleaving sites in protamine 2 are under purifying selection. Thus, most functionally important sites seem to be under purifying selection in protamine 1, and cleaved-protamine 2, suggesting that their main functional roles remain conserved. However, one site in a flanking region of a DNA-anchoring domain and two phosphorylation sites in protamine 1 are under positive selection suggesting that adaptive changes take place at specific functionally important sites which may modify the ability to bind to DNA. In contrast, functionally important sites in mature-protamine 2 evolve neutrally. This is consistent with the previous findings suggesting stronger selective constraints acting on protamine 1, and suggests that such constraints are even greater for functionally relevant sites.

In conclusion, among rodents protamine 1 and protamine 2 are under different selective constraints, since protamine 1 is under positive selection while cleaved and mature-protamine 2 are under relaxation. The role of sexual selection in rodents seems to be to halt the degree of degeneration of both domains of protamine 2, which in some species has even become dysfunctional and is not expressed in mature sperm. In contrast, protamine 1 is functionally conserved but shows directed positive selection on specific functional relevant sites such as DNA-anchoring domains and phosphorylation sites. While protamine 1 and mature-protamine 2 have many structural similarities, the cleaved region seems to be of retroviral origin. Our findings suggest that since genes in families have many structural and functional similarities, their evolutionary patterns should be studied jointly in order to understand how selective forces act upon all of them in cases where they may play complementary roles or have functional redundancies.

## Supporting Information

Figure S1 **Phylogenetic trees.** **A** - Tree of study species (Cricetidae). Input tree for branch and site analyses. *Mus m. musculus* was used as outgroup. **B** - Tree of study species (Cricetidae) including 12 rodent species as a background. *Oryctolagus cuniculus* was used as an outgroup. Input tree for clade analyses. Phylogenetic trees were constructed based on literature (Jaarola et al 2004 Mol Phylogenet Evol 33: 647–663; Galewski et al. 2006 BMC Evol Biol 6: 80; Neumann et al. 2006 Mol Phylogenet Evol 39: 135–148; Martín-Coello et al. 2009 Proc Roy Soc B 276: 2427; Gomez-Montoto et al. 2011 PLoS ONE 6: e18173).
(TIFF)

Figure S2 **PAML codeml clade analysis.** Models and analysis employed to detect the mode of selection acting on Protamine 1 and Protamine 2 domains. The employed models were compared by means of Likelihood-ratio-tests.
(TIFF)

Figure S3 **Amino acid sequence alignment of Protamine 1.** Study species (Cricetidae) represented by abbreviated code: *Arvicola sadipus* (ASA), *Arvicola terrestris* (ATE), *Clethrionomys glareolus* (CGL), *Cricetulus griseus* (CGR), *Chionomys nivalis* (CNI), *Microtus agrestis* (MAG), *Microtus arvalis* (MAR), *Mesocricetus auratus* (MAU), *Microtus cabrerae* (MCA), *Microtus gerbei* (MGE), *Mus musculus musculus* (MMU), *Phodopus campbelli* (PCM), *Pitymys duodecimcostatus* (PDU), *Pitymys lusitanicus* (PLU), *Phodopus roborovskii* (PRO), *Phodopus sungorus* (PSU), *Sigmodon hispidus* (SHI). Alignment of Protamine 1 including a histogram showing ω values (red line) estimated under model M2a in each site with standard error (bars). Arrows in Protamine 1 histogram indicate sites subjected to positive selection according to Codeml (PaML 4) site analysis (33, 38 and 39). Evidence for residue 38 was estimated under model M2a and for residues 33 and 39 under M2a and M8. Note that gaps are removed in the alignment but they were included in analysis.
(TIFF)

Figure S4 **Amino acid sequence alignment of cleaved-Protamine 2** Study species (Cricetidae) represented by abbreviated code: *Arvicola sadipus* (ASA), *Arvicola terrestris* (ATE), *Clethrionomys glareolus* (CGL), *Cricetulus griseus* (CGR), *Chionomys nivalis* (CNI), *Microtus agrestis* (MAG), *Microtus arvalis* (MAR), *Mesocricetus auratus* (MAU), *Microtus cabrerae* (MCA), *Microtus gerbei* (MGE), *Mus musculus musculus* (MMU), *Phodopus campbelli* (PCM), *Pitymys duodecimcostatus* (PDU), *Pitymys lusitanicus* (PLU), *Phodopus roborovskii* (PRO), *Phodopus sungorus* (PSU), *Sigmodon hispidus* (SHI). Alignment of cleaved-Protamine 2 including a histogram showing ω values (red line) estimated under model M2a in each site with standard error (bars). Note that gaps are removed in the alignment but they were included in analysis.
(TIFF)

Figure S5 **Amino acid sequence alignment of mature-Protamine 2.** Study species (Cricetidae) represented by abbreviated code: *Arvicola sadipus* (ASA), *Arvicola terrestris* (ATE), *Clethrionomys glareolus* (CGL), *Cricetulus griseus* (CGR), *Chionomys nivalis* (CNI), *Microtus agrestis* (MAG), *Microtus arvalis* (MAR), *Mesocricetus auratus* (MAU), *Microtus cabrerae* (MCA), *Microtus gerbei* (MGE), *Mus musculus musculus* (MMU), *Phodopus campbelli* (PCM), *Pitymys duodecimcostatus* (PDU), *Pitymys lusitanicus* (PLU), *Phodopus roborovskii* (PRO), *Phodopus sungorus* (PSU), *Sigmodon hispidus* (SHI). Alignment of mature-Protamine 2 including a histogram showing omega values (red line) estimated under model M2a in each site with standard error (bars). Note that gaps are removed in the alignment but they were included in analysis.
(TIFF)

Figure S6 **Codeml output file (M2) for Protamine 1.** Included species of Cricetidae represented by abbreviated code: *Arvicola sadipus* (ASA), *Arvicola terrestris* (ATE), *Clethrionomys glareolus* (CGL), *Cricetelus griseus* (CGR), *Chionomys nivalis* (CNI), *Microtus agrestis* (MAG), *Microtus arvalis* (MAR), *Mesocricetus auratus* (MAU), *Microtus cabrerae* (MCA), *Microtus gerbei* (MGE), *Mus musculus musculus* (MMU), *Phodopus campbelli* (PCM), *Pitymys duodecimcostatus* (PDU), *Pitymys lusitanicus* (PLU), *Phodopus roborovskii* (PRO), *Phodopus sungorus* (PSU), *Sigmodon hispidus* (SHI).
(PDF)

Figure S7 **Codeml output file (M2) for cleaved-Protamine 2.** Included species of Cricetidae represented by abbreviated code: *Arvicola sadipus* (ASA), *Arvicola terrestris* (ATE), *Clethrionomys glareolus* (CGL), *Cricetelus griseus* (CGR), *Chionomys nivalis* (CNI), *Microtus agrestis* (MAG), *Microtus arvalis* (MAR), *Mesocricetus auratus* (MAU), *Microtus cabrerae* (MCA), *Microtus gerbei* (MGE), *Mus musculus musculus* (MMU), *Phodopus campbelli* (PCM), *Pitymys duodecimcostatus* (PDU), *Pitymys lusitanicus* (PLU), *Phodopus roborovskii* (PRO), *Phodopus sungorus* (PSU), *Sigmodon hispidus* (SHI).
(PDF)

Figure S8 **Codeml output file (M2) for mature-Protamine 2.** Included species of Cricetidae represented by abbreviated code: *Arvicola sadipus* (ASA), *Arvicola terrestris* (ATE), *Clethrionomys glareolus* (CGL), *Cricetelus griseus* (CGR), *Chionomys nivalis* (CNI), *Microtus agrestis* (MAG), *Microtus arvalis* (MAR), *Mesocricetus auratus* (MAU), *Microtus cabrerae* (MCA), *Microtus gerbei* (MGE), *Mus musculus musculus* (MMU), *Phodopus campbelli* (PCM), *Pitymys duodecimcostatus* (PDU), *Pitymys lusitanicus* (PLU), *Phodopus roborovskii* (PRO), *Phodopus sungorus* (PSU), *Sigmodon hispidus* (SHI).
(PDF)
