# Supplementary figures and images for: Sexual Selection Halts the Relaxation of Protamine 2 among Rodents
Source: PLoS One. 2011 Dec 21;6(12):e29247. doi: 10.1371/journal.pone.0029247 (PMC3244444; doi:10.1371/journal.pone.0029247)

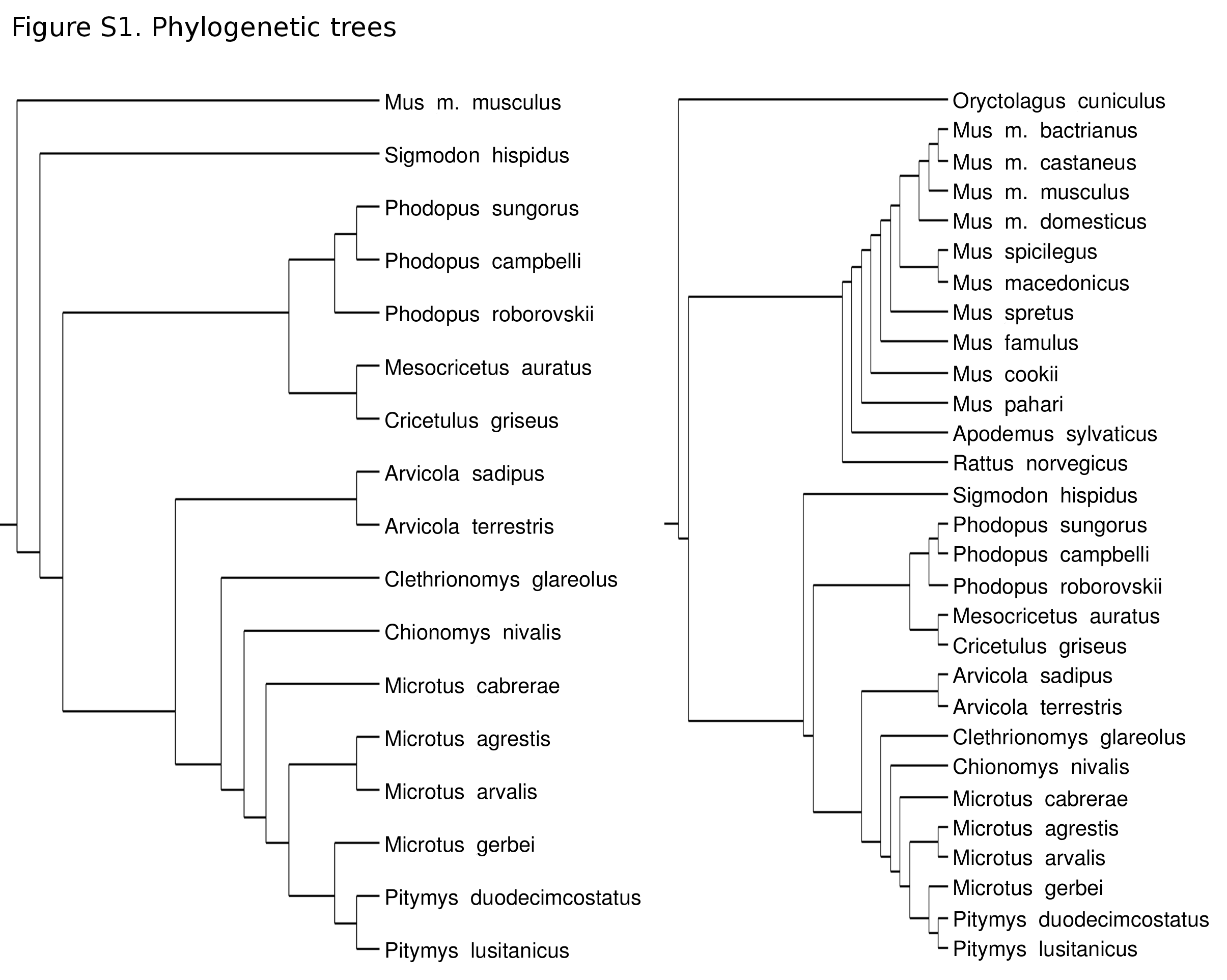

Supplement: Figure S1 — Phylogenetic trees. A - Tree of study species (Cricetidae). Input tree for branch and site analyses. Mus m. musculus was used as outgroup. B - Tree of study species (Cricetidae) including 12 rodent species as a background. Oryctolagus cuniculus was used as an outgroup. Input tree for clade analyses. Phylogenetic trees were constructed based on literature (Jaarola et al 2004 Mol Phylogenet Evol 33: 647–663; Galewski et al. 2006 BMC Evol Biol 6: 80; Neumann et al. 2006 Mol Phylogenet Evol 39: 135–148; Martín-Coello et al. 2009 Proc Roy Soc B 276: 2427; Gomez-Montoto et al. 2011 PLoS ONE 6: e18173). (TIFF) [file pone.0029247.s001.tiff]

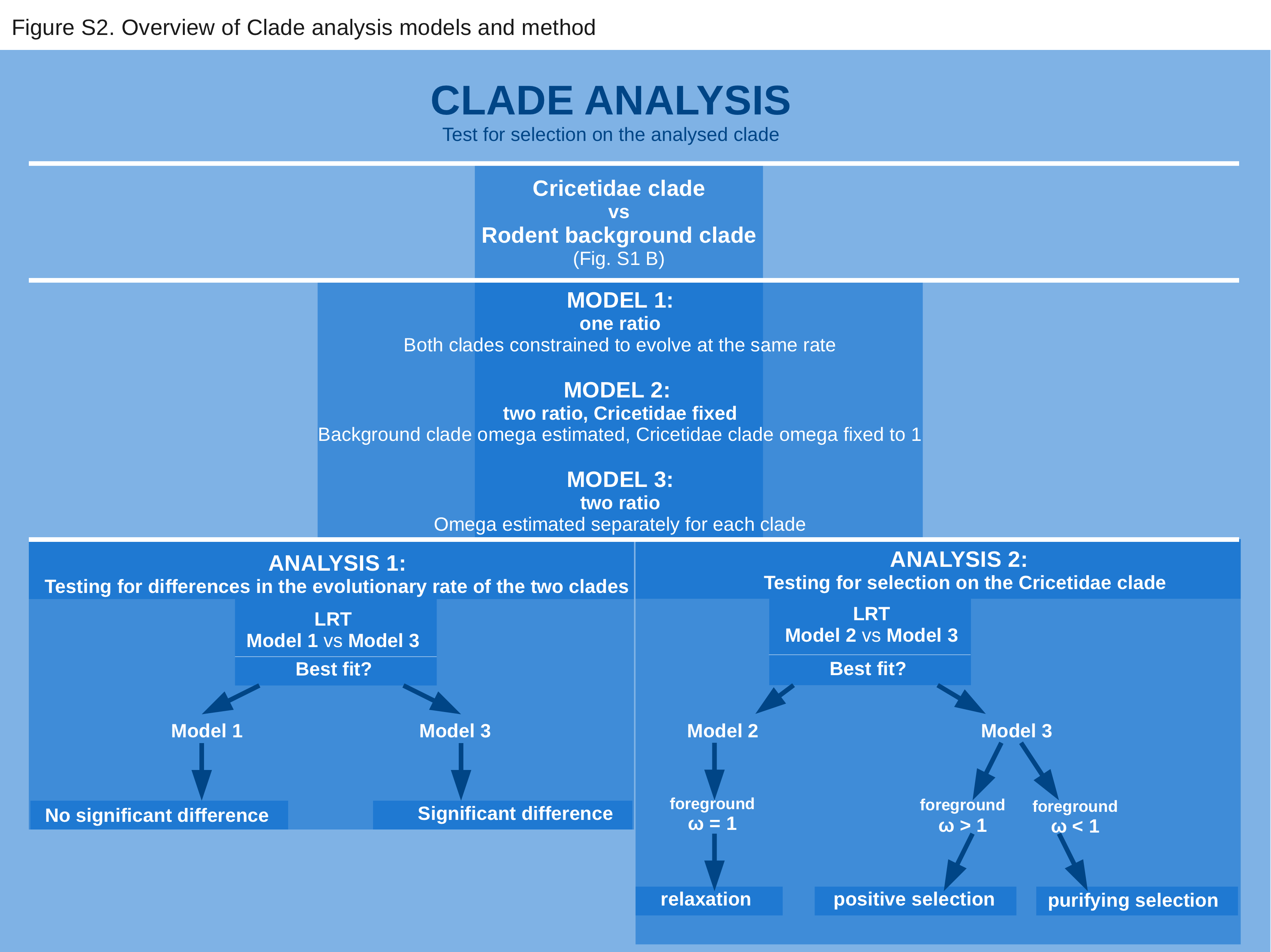

Supplement: Figure S2 — PAML codeml clade analysis. Models and analysis employed to detect the mode of selection acting on Protamine 1 and Protamine 2 domains. The employed models were compared by means of Likelihood-ratio-tests. (TIFF) [file pone.0029247.s002.tiff]

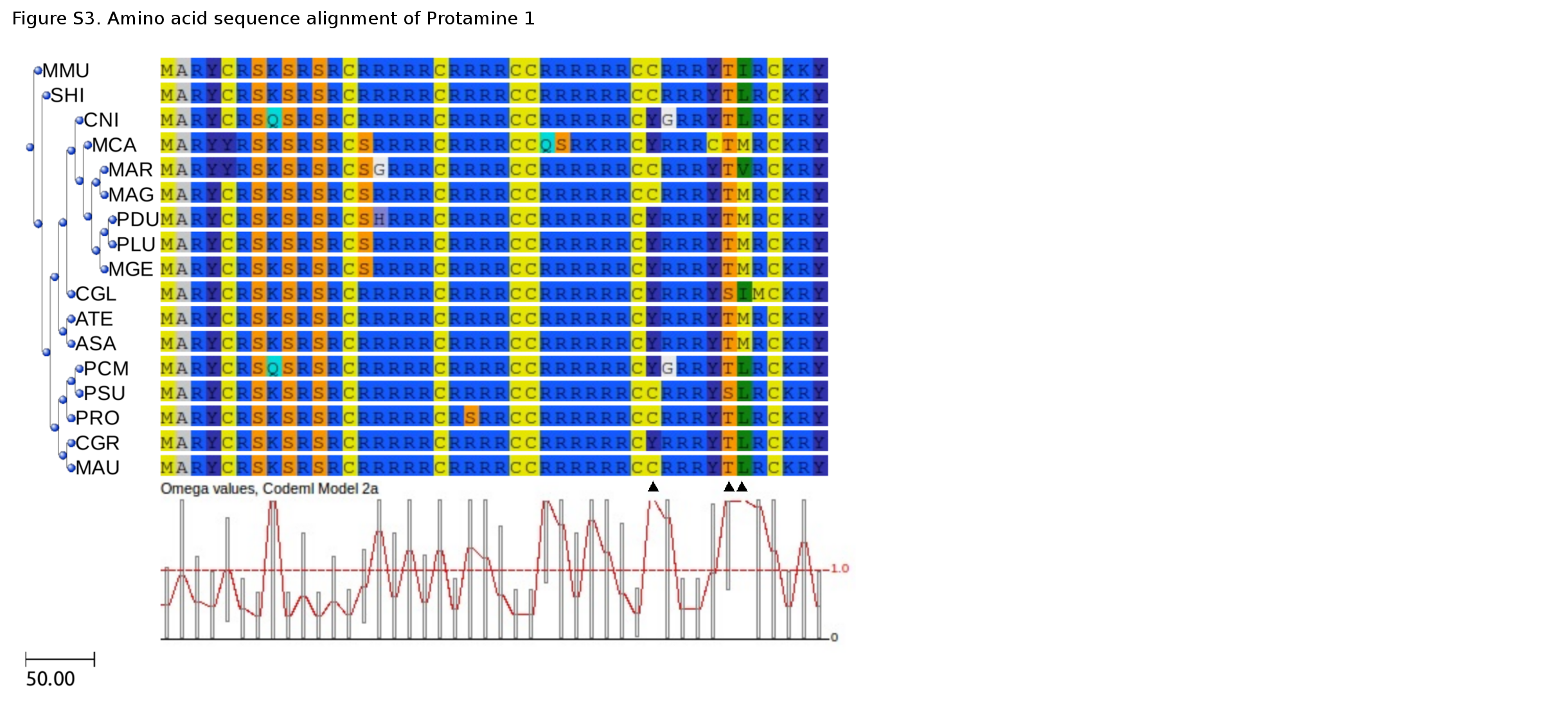

Supplement: Figure S3 — Amino acid sequence alignment of Protamine 1. Study species (Cricetidae) represented by abbreviated code: Arvicola sadipus (ASA), Arvicola terrestris (ATE), Clethrionomys glareolus (CGL), Cricetulus griseus (CGR), Chionomys nivalis (CNI), Microtus agrestis (MAG), Microtus arvalis (MAR), Mesocricetus auratus (MAU), Microtus cabrerae (MCA), Microtus gerbei (MGE), Mus musculus musculus (MMU), Phodopus campbelli (PCM), Pitymys duodecimcostatus (PDU), Pitymys lusitanicus (PLU), Phodopus roborovskii (PRO), Phodopus sungorus (PSU), Sigmodon hispidus (SHI). Alignment of Protamine 1 including a histogram showing ω values (red line) estimated under model M2a in each site with standard error (bars). Arrows in Protamine 1 histogram indicate sites subjected to positive selection according to Codeml (PaML 4) site analysis (33, 38 and 39). Evidence for residue 38 was estimated under model M2a and for residues 33 and 39 under M2a and M8. Note that gaps are removed in the alignment but they were included in analysis. (TIFF) [file pone.0029247.s003.tiff]

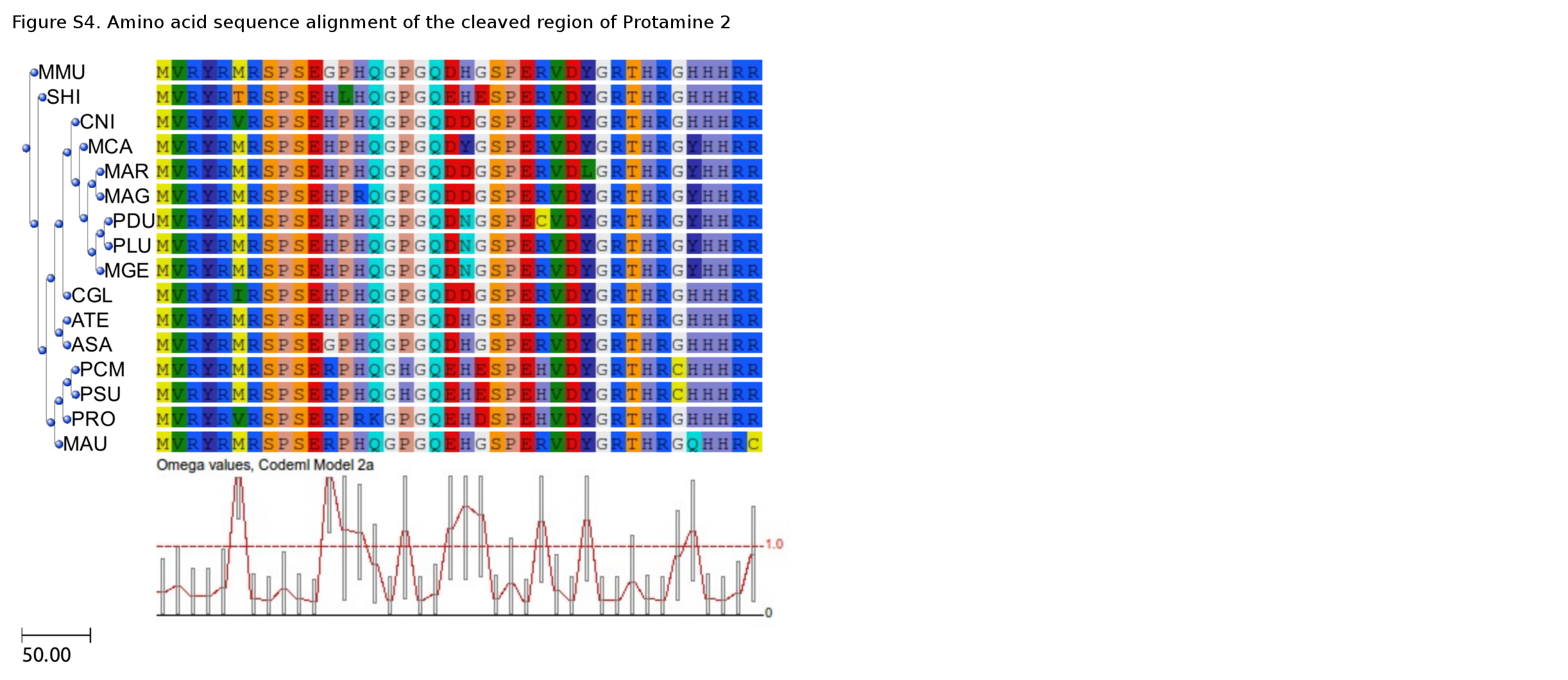

Supplement: Figure S4 — Amino acid sequence alignment of cleaved-Protamine 2 Study species (Cricetidae) represented by abbreviated code: Arvicola sadipus (ASA), Arvicola terrestris (ATE), Clethrionomys glareolus (CGL), Cricetulus griseus (CGR), Chionomys nivalis (CNI), Microtus agrestis (MAG), Microtus arvalis (MAR), Mesocricetus auratus (MAU), Microtus cabrerae (MCA), Microtus gerbei (MGE), Mus musculus musculus (MMU), Phodopus campbelli (PCM), Pitymys duodecimcostatus (PDU), Pitymys lusitanicus (PLU), Phodopus roborovskii (PRO), Phodopus sungorus (PSU), Sigmodon hispidus (SHI). Alignment of cleaved-Protamine 2 including a histogram showing ω values (red line) estimated under model M2a in each site with standard error (bars). Note that gaps are removed in the alignment but they were included in analysis. (TIFF) [file pone.0029247.s004.tiff]

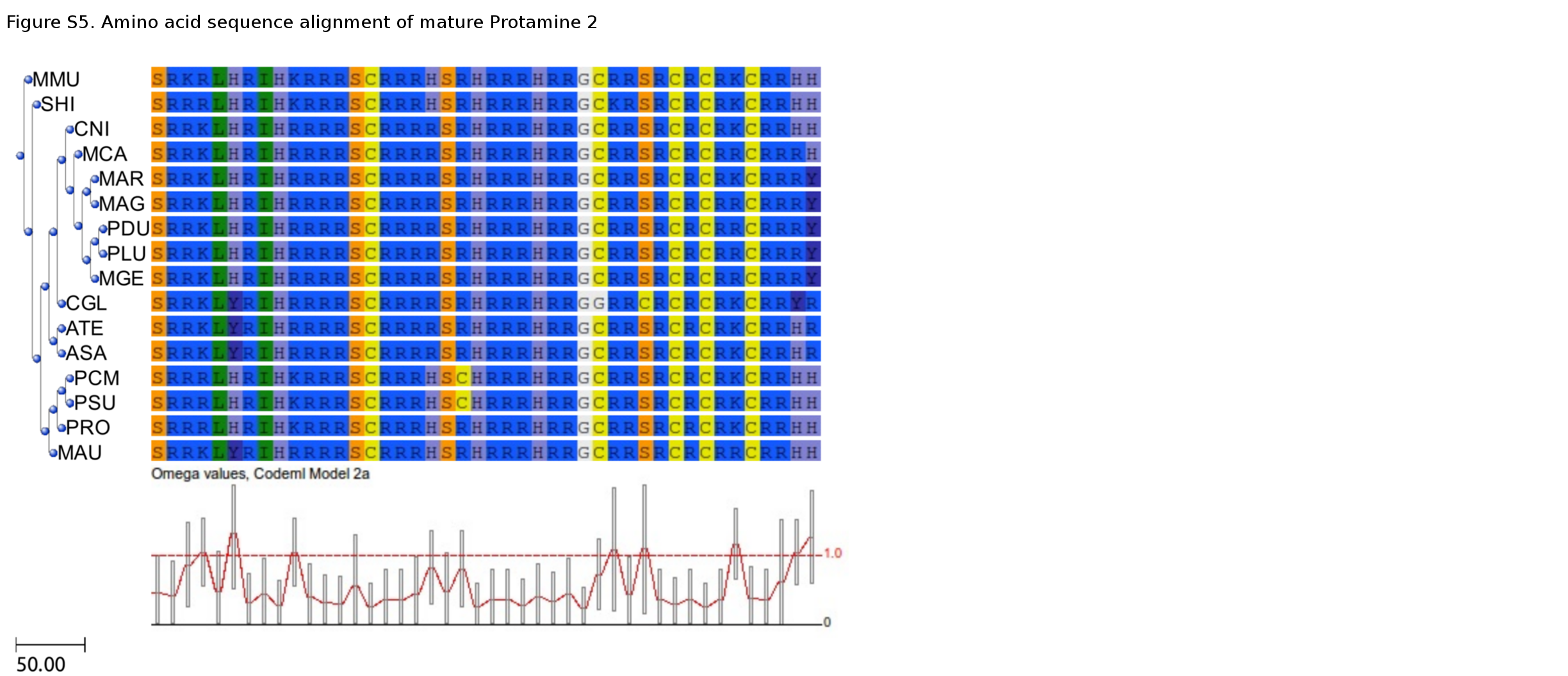

Supplement: Figure S5 — Amino acid sequence alignment of mature-Protamine 2. Study species (Cricetidae) represented by abbreviated code: Arvicola sadipus (ASA), Arvicola terrestris (ATE), Clethrionomys glareolus (CGL), Cricetulus griseus (CGR), Chionomys nivalis (CNI), Microtus agrestis (MAG), Microtus arvalis (MAR), Mesocricetus auratus (MAU), Microtus cabrerae (MCA), Microtus gerbei (MGE), Mus musculus musculus (MMU), Phodopus campbelli (PCM), Pitymys duodecimcostatus (PDU), Pitymys lusitanicus (PLU), Phodopus roborovskii (PRO), Phodopus sungorus (PSU), Sigmodon hispidus (SHI). Alignment of mature-Protamine 2 including a histogram showing omega values (red line) estimated under model M2a in each site with standard error (bars). Note that gaps are removed in the alignment but they were included in analysis. (TIFF) [file pone.0029247.s005.tiff]
