# Supplementary material for: Sexual Selection Halts the Relaxation of Protamine 2 among Rodents
Source: PLoS One. 2011 Dec 21;6(12):e29247. doi: 10.1371/journal.pone.0029247 (PMC3244444; doi:10.1371/journal.pone.0029247)
Supplement: Figure S6 — Codeml output file (M2) for Protamine 1. Included species of Cricetidae represented by abbreviated code: Arvicola sadipus (ASA), Arvicola terrestris (ATE), Clethrionomys glareolus (CGL), Cricetelus griseus (CGR), Chionomys nivalis (CNI), Microtus agrestis (MAG), Microtus arvalis (MAR), Mesocricetus auratus (MAU), Microtus cabrerae (MCA), Microtus gerbei (MGE), Mus musculus musculus (MMU), Phodopus campbelli (PCM), Pitymys duodecimcostatus (PDU), Pitymys lusitanicus (PLU), Phodopus roborovskii (PRO), Phodopus sungorus (PSU), Sigmodon hispidus (SHI). (PDF) [file pone.0029247.s006.pdf]

Figure S6. Protamine 1
Codeml output file M2

Arvicola sadipus (ASA)
Arvicola terrestris (ATE)
Clethrionomys glareolus (CGL)
Cricetelus griseus (CGR)
Chionomys nivalis (CNI)
Microtus agrestis (MAG)
Microtus arvalis (MAR)
Mesocricetus auratus (MAU)
Microtus cabrerae (MCA)
Microtus gerbei (MGE)
Mus musculus musculus (MMU)
Phodopus campbelli (PCM)
Pitymys duodecimcostatus (PDU)
Pitymys lusitanicus (PLU)
Phodopus roborovskii (PRO)
Phodopus sugorus (PSU)
Sigmodon hispidus (SHI)

```
     17    153

MAR  ATG GCC AGA TAC CG- --C TAC CGC AGC AAA AGC AGG AGC AGA TGC AGC CGC GGC AGG CGG AGA TGT CGC AGA AGG AGG AGG CGA TGC TGC CGG AGG AGG AGG CGA AGG TGC TGC CGC CGC CGC CGC ACA TAC ACT GTG AGG TGT AAA AGA TAC
MAG  ATG GCC AGA TAC CG- --C TGC CGT AGC AAA AGC AGG AGC AGA TGC AGC CGC CGC AGG CGG AGA TGT CGC AGA CGG AGG AGG CGA TGC TGC CGG AGG AGG AGG CGA AGG TGC TGC CGC CGC CGC CGC ACA TAC ACC ATG AGG TGT AAA AGA TAC
SHI  ATG GCC AGA TAC CGA TGC TGT CGA AGC AAA AGC AGG AGC AGA TGC CGC CGC CGC AGG CGA AGA TGT CGC AGA CGG AGG AGG CGA TGC TGC CGG AGG AGG AGG CGA AGA TGC TGC CGC CGC CGC CGC ACA TAC ACC CTA AGG TGT AAA AAA TAC
MMU  ATG GCC AGA TAC CGA TGC TGC CGC AGC AAA AGC AGG AGC AGA TGC CGC CGT CGC AGG CGA AGA TGT CGC AGA CGG AGG AGG CGA TGC TGC CGG CGG AGG AGG CGA AGA TGC TGC CGT CGC CGC CGC TCA TAC ACC ATA AGG TGT AAA AAA TAC
MAU  ATG GCC AGA TAC CGA TGC TGC CGC AGC AAA AGC AGG AGC AGA TGC CGC CGC CGC AGG CGG CGA TGT CGC AGA CGG AGG AGG CGA TGC TGC CGG AGG AGG AGG CGA AGA TGC TGC CGC CGC CGC CGC ACA TAC ACC CTA AGG TGT AAA AGA TAC
PSU  ATG GCC AGA TAC CGA TGC TGC CGC AGC AAA AGC AGG AGC AGA TGC CGC CGC CGC AGG CGA AGA TGT CGC AGA CGG AGG AGG CGA TGC TGC CGG AGG AGG AGG CGA AGG TGC TGC CGC CGC CGC CG- --A TAC TCC CTG AGG TGT AAA AGA TAC
PRO  ATG GCC AGA TAC CGA TGC TGC CGC AGC AAA AGC AGG AGC AGA TGC CGC CGC CGC AGG CGG AGA TGT CGC AGT CGG AGG AGG CGA TGC TGC CGG CGG AGG AGG CGA AGG TGC TGC CGC CGC CGC AG- --A TAC ACC CTG AGG TGT AAA AGA TAC
CGR  ATG GCC AGA TAC CGA TGC TGC CGC AGC AAA AGC AGG AGC AGA TGC CGC CGC CGC AGG CGA AGA TGT CGC AGA CGG AGA AGG CGA TGC TGC CGG AGG AGG AGG CGA AGG TGT TAC CGC CGC CGC CGT ACA TAC ACC CTA AGG TGT AAA AGG TAC
CGL  ATG GCC AGA TAC CG- --C TGC CGC AGC AAA AGC AGG AGC AGA TGC CGC CGT CGC AGG CGG AGA TGT CGC AGA CGG AGG AG- --A TGC TGC CGG AGG AGG AGG CGA AGG TGC TAC CGC CGC CGC CGC TCA TAC TCC ATA ATG TGT AAA AGA TAC
ASA  ATG GCC AGA TAC CG- --C TGC CGC AGC AAA AGC AGG AGC AGA TGC CGC CGC CGC AGG CGG AGA TGT CGC AGA CGG AGG AGG CGA TGC TGC CGG AGG AGG AGG CGA AGG TGC TAC CGC CGC CGC CGC ACA TAC ACC ATG AGG TGT AAA AGA TAC
ATE  ATG GCC AGA TAC CG- --C TGC CGC AGC AAA AGC AGG AGC AGA TGC CGC CRC CGC AGG CGG AGA TGT CGC AGA CGG AGG AGG CGA TGC TGC CGG AGG AGG AGG CGA AGG TGC TAC CGC CGC CGC CGC ACA TAC ACC ATG AGG TGT AAA AGA TAC
CNI  ATG GCC AGA TAC CG- --C TGC CGC AGC CAA AGC AGG AGC AGA TGC CGC CGC CGC AGG CGG AGA TGT CGC AGA CGG AGG AGG CGA TGC TGC CGG AGG AGG AGG CGA AGG TGC TAC GGC CGC CGC CGC ACA TAC ACC CTG AGG TGT AAA AGA TAC
PCM  ATG GCC AGA TAC CG- --C TGC CGC AGC CAA AGC AGG AGC AGA TGC CGC CGC CGC AGG CGG AGA TGT CGC AGA CGG AGG AGG CGA TGC TGC CGG AGG AGG AGG CGA AGG TGC TAC GGC CGC CGC CGC ACA TAC ACC CTG AGG TGT AAA AGA TAC
MGE  ATG GCC AGA TAC CG- --C TGC CGC AGC AAA AGC AGG AGC AGA TGC AGC CGC CGC AGG CGG AGA TGT CGC AGA AGG AGG AGG CGA TGC TGC CGG AGG AGG AGG CGA AGG TGC TAC CGC CGC CGC CGC TCA TAC ACC ATG AGG TGT AAA AGA TAC
PDU  ATG GCC AGA TAC CG- --C TGC CGC AGC AAA AGC AGG AGC AGA TGC AGC CGC CAC AGG CGG AGA TGT CGC AGA AGG AGG AGG CGA TGC TGC CGG AGG AGG AGG CGA AGG TGC TAC CGC CGC CGC CGC TCA TAC ACC ATG AGG TGT AAA AGA TAC
PLU  ATG GCC AGA TAC CG- --C TGC CGC AGC AAA AGC AGG AGC AGA TGC AGC CGC CGC AGG CGG AGA TGT CGC AGA AGG AGG AGG CGA TGC TGC CGG AGG AGG AGG CGA AGG TGC TAC CGC CGC CGC CGC TCA TAC ACC ATG AGG TGT AAA AGA TAC
MCA  ATG GCC AGA TAC CG- --C TAC CGC AGC AAA AGC AGG AGC AGA TGC AGC CGC CGC AGG CGG AGA TGT CGC AGA CGG AGG AGG CGA TGC TGC CAG AGC AGG AAG CGA AGG TGC TAC CGT CGC CGC CGC TCA TGC ACC ATG AGG TGT AAA AGA TAC
```

Printing out site pattern counts

```
        17        120  P

MAR   --C AAA AAA ACA ACT AGA AGA AGA AGA AGC AGC AGG AGG AGG AGG AGG AGG AGG AGG ATG CG- CGA CGA CGC CGC CGC CGC CGC CGG CGG GCC GGC GTG TAC TAC TAC TGC TGC TGC TGT
MAG   --C AAA AAA ACA ACC AGA AGA AGA AGA AGC AGC AGG AGG AGG AGG AGG AGG AGG CGG ATG CG- CGA CGA CGC CGC CGC CGC CGT CGG CGG GCC CGC ATG TAC TAC TGC TGC TGC TGC TGT
SHI   TGC AAA AAA ACA ACC AAA AGA AGA AGA AGC CGC AGA AGG AGG AGG AGG AGG AGG CGG ATG CGA CGA CGA CGC CGC CGC CGC CGA CGA CGG GCC CGC CTA TAC TAC TGT TGC TGC TGC TGT
MMU   TGC AAA AAA TCA ACC AAA AGA AGA AGA AGC CGC AGA AGG AGG AGG AGG AGG CGG CGG ATG CGA CGA CGA CGC CGC CGT CGT CGC CGA CGG GCC CGC ATA TAC TAC TGC TGC TGC TGC TGT
```

```
MAU   TGC AAA AAA ACA ACC AGA AGA AGA CGA AGC CGC AGA AGG AGG AGG AGG AGG AGG CGG ATG CGA CGA CGA CGC CGC CGC CGC CGC CGG CGG GCC CGC CTA TAC TAC TGC TGC TGC TGC TGT
PSU   TGC AAA AAA --A TCC AGA AGA AGA AGA AGC CGC AGG AGG AGG AGG AGG AGG AGG CGG ATG CGA CGA CGA CG- CGC CGC CGC CGC CGA CGG GCC CGC CTG TAC TAC TGC TGC TGC TGC TGT
PRO   TGC AAA AAA --A ACC AGA AGA AGT AGA AGC CGC AGG AGG AGG AGG AGG AGG CGG CGG ATG CGA CGA CGA AG- CGC CGC CGC CGC CGG CGG GCC CGC CTG TAC TAC TGC TGC TGC TGC TGT
CGR   TGC AAA AAA ACA ACC AGG AGA AGA AGA AGC CGC AGG AGA AGG AGG AGG AGG AGG CGG ATG CGA CGA CGA CGT CGC CGC CGC CGC CGA CGG GCC CGC CTA TAC TAC TGC TAC TGC TGT TGT
CGL   --C AAA AAA TCA TCC AGA AGA AGA AGA AGC CGC AGG AGG AG- AGG AGG ATG AGG CGG ATG CG- --A CGA CGC CGC CGC CGT CGC CGG CGG GCC CGC ATA TAC TAC TGC TAC TGC TGC TGT
ASA   --C AAA AAA ACA ACC AGA AGA AGA AGA AGC CGC AGG AGG AGG AGG AGG AGG AGG CGG ATG CG- CGA CGA CGC CGC CGC CGC CGC CGG CGG GCC CGC ATG TAC TAC TGC TAC TGC TGC TGT
ATE   --C AAA AAA ACA ACC AGA AGA AGA AGA AGC CGC AGG AGG AGG AGG AGG AGG AGG CGG ATG CG- CGA CGA CGC CGC CGC CRC CGC CGG CGG GCC CGC ATG TAC TAC TGC TAC TGC TGC TGT
CNI   --C AAA CAA ACA ACC AGA AGA AGA AGA AGC CGC AGG AGG AGG AGG AGG AGG AGG CGG ATG CG- CGA CGA CGC CGC GGC CGC CGC CGG CGG GCC CGC CTG TAC TAC TGC TAC TGC TGC TGT
PCM   --C AAA CAA ACA ACC AGA AGA AGA AGA AGC CGC AGG AGG AGG AGG AGG AGG AGG CGG ATG CG- CGA CGA CGC CGC GGC CGC CGC CGG CGG GCC CGC CTG TAC TAC TGC TAC TGC TGC TGT
MGE   --C AAA AAA TCA ACC AGA AGA AGA AGA AGC AGC AGG AGG AGG AGG AGG AGG AGG AGG ATG CG- CGA CGA CGC CGC CGC CGC CGC CGG CGG GCC CGC ATG TAC TAC TGC TAC TGC TGC TGT
PDU   --C AAA AAA TCA ACC AGA AGA AGA AGA AGC AGC AGG AGG AGG AGG AGG AGG AGG AGG ATG CG- CGA CGA CGC CGC CGC CGC CGC CGG CGG GCC CAC ATG TAC TAC TGC TAC TGC TGC TGT
PLU   --C AAA AAA TCA ACC AGA AGA AGA AGA AGC AGC AGG AGG AGG AGG AGG AGG AGG AGG ATG CG- CGA CGA CGC CGC CGC CGC CGC CGG CGG GCC CGC ATG TAC TAC TGC TAC TGC TGC TGT
MCA   --C AAA AAA TCA ACC AGA AGA AGA AGA AGC AGC AGG AGG AGG AAG AGG AGG AGC CGG ATG CG- CGA CGA CGC CGC CGT CGC CGC CGG CAG GCC CGC ATG TAC TGC TAC TAC TGC TGC TGT



    1    1    1    1    1    1    2    1    1    3    1    1    1    1    1
    3    1    1    1    1    1    1    1    1    3    1    1    1    1    1
    1    1    1    2    1    1    1    3    1    2

CODONML (in paml version 4.4d, March 2011)  1.phy
Model: One dN/dS ratio for branches
Codon frequency model: Fcodon
Site-class models:  PositiveSelection
ns =  17  ls =  51

Codon usage in sequences
--------------------------------------------------------------------------------------------------------------
Phe TTT  0  0  0  0  0  0 | Ser TCT  0  0  0  0  0  0 | Tyr TAT  0  0  0  0  0  0 | Cys TGT  2  2  3  2  2  2
    TTC  0  0  0  0  0  0 |     TCC  0  0  0  0  0  1 |     TAC  4  3  3  3  3  3 |     TGC  5  6  6  7  7  7
Leu TTA  0  0  0  0  0  0 |     TCA  0  0  0  1  0  0 | *** TAA  0  0  0  0  0  0 | *** TGA  0  0  0  0  0  0
    TTG  0  0  0  0  0  0 |     TCG  0  0  0  0  0  0 |     TAG  0  0  0  0  0  0 | Trp TGG  0  0  0  0  0  0
--------------------------------------------------------------------------------------------------------------
Leu CTT  0  0  0  0  0  0 | Pro CCT  0  0  0  0  0  0 | His CAT  0  0  0  0  0  0 | Arg CGT  0  1  0  2  0  0
    CTC  0  0  0  0  0  0 |     CCC  0  0  0  0  0  0 |     CAC  0  0  0  0  0  0 |     CGC  7  7  8  7  9  8
    CTA  0  0  1  0  1  0 |     CCA  0  0  0  0  0  0 | Gln CAA  0  0  0  0  0  0 |     CGA  2  2  5  4  4  4
    CTG  0  0  0  0  0  1 |     CCG  0  0  0  0  0  0 |     CAG  0  0  0  0  0  0 |     CGG  2  3  2  3  3  2
--------------------------------------------------------------------------------------------------------------
Ile ATT  0  0  0  0  0  0 | Thr ACT  1  0  0  0  0  0 | Asn AAT  0  0  0  0  0  0 | Ser AGT  0  0  0  0  0  0
    ATC  0  0  0  0  0  0 |     ACC  0  1  1  1  1  0 |     AAC  0  0  0  0  0  0 |     AGC  4  4  3  3  3  3
    ATA  0  0  0  1  0  0 |     ACA  1  1  1  0  1  0 | Lys AAA  2  2  3  3  2  2 | Arg AGA  5  5  5  5  5  5
Met ATG  1  2  1  1  1  1 |     ACG  0  0  0  0  0  0 |     AAG  0  0  0  0  0  0 |     AGG 10  9  8  7  8  9
--------------------------------------------------------------------------------------------------------------
Val GTT  0  0  0  0  0  0 | Ala GCT  0  0  0  0  0  0 | Asp GAT  0  0  0  0  0  0 | Gly GGT  0  0  0  0  0  0
    GTC  0  0  0  0  0  0 |     GCC  1  1  1  1  1  1 |     GAC  0  0  0  0  0  0 |     GGC  1  0  0  0  0  0
    GTA  0  0  0  0  0  0 |     GCA  0  0  0  0  0  0 | Glu GAA  0  0  0  0  0  0 |     GGA  0  0  0  0  0  0
    GTG  1  0  0  0  0  0 |     GCG  0  0  0  0  0  0 |     GAG  0  0  0  0  0  0 |     GGG  0  0  0  0  0  0
--------------------------------------------------------------------------------------------------------------

--------------------------------------------------------------------------------------------------------------
Phe TTT  0  0  0  0  0  0 | Ser TCT  0  0  0  0  0  0 | Tyr TAT  0  0  0  0  0  0 | Cys TGT  2  3  2  2  2  2
    TTC  0  0  0  0  0  0 |     TCC  0  0  1  0  0  0 |     TAC  3  4  4  4  4  4 |     TGC  7  5  5  5  5  5
Leu TTA  0  0  0  0  0  0 |     TCA  0  0  1  0  0  0 | *** TAA  0  0  0  0  0  0 | *** TGA  0  0  0  0  0  0
    TTG  0  0  0  0  0  0 |     TCG  0  0  0  0  0  0 |     TAG  0  0  0  0  0  0 | Trp TGG  0  0  0  0  0  0
--------------------------------------------------------------------------------------------------------------
Leu CTT  0  0  0  0  0  0 | Pro CCT  0  0  0  0  0  0 | His CAT  0  0  0  0  0  0 | Arg CGT  0  1  1  0  0  0
    CTC  0  0  0  0  0  0 |     CCC  0  0  0  0  0  0 |     CAC  0  0  0  0  0  0 |     CGC  8  8  8  9  8  8
    CTA  0  1  0  0  0  0 |     CCA  0  0  0  0  0  0 | Gln CAA  0  0  0  0  0  1 |     CGA  3  4  1  2  2  2
```

```
    CTG  1  0  0  0  0  1 |     CCG  0  0  0  0  0  0 |     CAG  0  0  0  0  0  0 |     CGG  4  2  3  3  3  3
--------------------------------------------------------------------------------------------------------------
Ile ATT  0  0  0  0  0  0 | Thr ACT  0  0  0  0  0  0 | Asn AAT  0  0  0  0  0  0 | Ser AGT  1  0  0  0  0  0
    ATC  0  0  0  0  0  0 |     ACC  1  1  0  1  1  1 |     AAC  0  0  0  0  0  0 |     AGC  3  3  3  3  3  3
    ATA  0  0  1  0  0  0 |     ACA  0  1  0  1  1  1 | Lys AAA  2  2  2  2  2  1 | Arg AGA  4  5  5  5  5  5
Met ATG  1  1  2  2  2  1 |     ACG  0  0  0  0  0  0 |     AAG  0  0  0  0  0  0 |     AGG  8  9  7  9  9  9
--------------------------------------------------------------------------------------------------------------
Val GTT  0  0  0  0  0  0 | Ala GCT  0  0  0  0  0  0 | Asp GAT  0  0  0  0  0  0 | Gly GGT  0  0  0  0  0  0
    GTC  0  0  0  0  0  0 |     GCC  1  1  1  1  1  1 |     GAC  0  0  0  0  0  0 |     GGC  0  0  0  0  0  1
    GTA  0  0  0  0  0  0 |     GCA  0  0  0  0  0  0 | Glu GAA  0  0  0  0  0  0 |     GGA  0  0  0  0  0  0
    GTG  0  0  0  0  0  0 |     GCG  0  0  0  0  0  0 |     GAG  0  0  0  0  0  0 |     GGG  0  0  0  0  0  0
--------------------------------------------------------------------------------------------------------------

--------------------------------------------------------------------------------------------------
Phe TTT  0  0  0  0  0 | Ser TCT  0  0  0  0  0 | Tyr TAT  0  0  0  0  0 | Cys TGT  2  2  2  2  2
    TTC  0  0  0  0  0 |     TCC  0  0  0  0  0 |     TAC  4  4  4  4  4 |     TGC  5  5  5  5  5
Leu TTA  0  0  0  0  0 |     TCA  0  1  1  1  1 | *** TAA  0  0  0  0  0 | *** TGA  0  0  0  0  0
    TTG  0  0  0  0  0 |     TCG  0  0  0  0  0 |     TAG  0  0  0  0  0 | Trp TGG  0  0  0  0  0
--------------------------------------------------------------------------------------------------
Leu CTT  0  0  0  0  0 | Pro CCT  0  0  0  0  0 | His CAT  0  0  0  0  0 | Arg CGT  0  0  0  0  1
    CTC  0  0  0  0  0 |     CCC  0  0  0  0  0 |     CAC  0  0  1  0  0 |     CGC  8  8  7  8  7
    CTA  0  0  0  0  0 |     CCA  0  0  0  0  0 | Gln CAA  1  0  0  0  0 |     CGA  2  2  2  2  2
    CTG  1  0  0  0  0 |     CCG  0  0  0  0  0 |     CAG  0  0  0  0  1 |     CGG  3  2  2  2  2
--------------------------------------------------------------------------------------------------
Ile ATT  0  0  0  0  0 | Thr ACT  0  0  0  0  0 | Asn AAT  0  0  0  0  0 | Ser AGT  0  0  0  0  0
    ATC  0  0  0  0  0 |     ACC  1  1  1  1  1 |     AAC  0  0  0  0  0 |     AGC  3  4  4  4  5
    ATA  0  0  0  0  0 |     ACA  1  0  0  0  0 | Lys AAA  1  2  2  2  2 | Arg AGA  5  5  5  5  5
Met ATG  1  2  2  2  2 |     ACG  0  0  0  0  0 |     AAG  0  0  0  0  1 |     AGG  9 10 10 10  7
--------------------------------------------------------------------------------------------------
Val GTT  0  0  0  0  0 | Ala GCT  0  0  0  0  0 | Asp GAT  0  0  0  0  0 | Gly GGT  0  0  0  0  0
    GTC  0  0  0  0  0 |     GCC  1  1  1  1  1 |     GAC  0  0  0  0  0 |     GGC  1  0  0  0  0
    GTA  0  0  0  0  0 |     GCA  0  0  0  0  0 | Glu GAA  0  0  0  0  0 |     GGA  0  0  0  0  0
    GTG  0  0  0  0  0 |     GCG  0  0  0  0  0 |     GAG  0  0  0  0  0 |     GGG  0  0  0  0  0
--------------------------------------------------------------------------------------------------

Codon position x base (3x4) table for each sequence.

#1: MAR
position  1:    T:0.22449    C:0.22449    A:0.48980    G:0.06122
position  2:    T:0.04082    C:0.06122    A:0.12245    G:0.77551
position  3:    T:0.06122    C:0.44898    A:0.20408    G:0.28571
Average         T:0.10884    C:0.24490    A:0.27211    G:0.37415

#2: MAG
position  1:    T:0.22449    C:0.26531    A:0.48980    G:0.02041
position  2:    T:0.04082    C:0.06122    A:0.10204    G:0.79592
position  3:    T:0.06122    C:0.44898    A:0.20408    G:0.28571
Average         T:0.10884    C:0.25850    A:0.26531    G:0.36735

#3: SHI
position  1:    T:0.23529    C:0.31373    A:0.43137    G:0.01961
position  2:    T:0.03922    C:0.05882    A:0.11765    G:0.78431
position  3:    T:0.05882    C:0.43137    A:0.29412    G:0.21569
Average         T:0.11111    C:0.26797    A:0.28105    G:0.33987

#4: MMU
position  1:    T:0.25490    C:0.31373    A:0.41176    G:0.01961
position  2:    T:0.03922    C:0.05882    A:0.11765    G:0.78431
```

```
position  3:    T:0.07843    C:0.43137    A:0.27451    G:0.21569
Average         T:0.12418    C:0.26797    A:0.26797    G:0.33987

#5: MAU
position  1:    T:0.23529    C:0.33333    A:0.41176    G:0.01961
position  2:    T:0.03922    C:0.05882    A:0.09804    G:0.80392
position  3:    T:0.03922    C:0.47059    A:0.25490    G:0.23529
Average         T:0.10458    C:0.28758    A:0.25490    G:0.35294

#6: PSU
position  1:    T:0.26531    C:0.30612    A:0.40816    G:0.02041
position  2:    T:0.04082    C:0.04082    A:0.10204    G:0.81633
position  3:    T:0.04082    C:0.46939    A:0.22449    G:0.26531
Average         T:0.11565    C:0.27211    A:0.24490    G:0.36735

#7: PRO
position  1:    T:0.24490    C:0.32653    A:0.40816    G:0.02041
position  2:    T:0.04082    C:0.04082    A:0.10204    G:0.81633
position  3:    T:0.06122    C:0.46939    A:0.18367    G:0.28571
Average         T:0.11565    C:0.27891    A:0.23129    G:0.37415

#8: CGR
position  1:    T:0.23529    C:0.31373    A:0.43137    G:0.01961
position  2:    T:0.03922    C:0.05882    A:0.11765    G:0.78431
position  3:    T:0.07843    C:0.43137    A:0.25490    G:0.23529
Average         T:0.11765    C:0.26797    A:0.26797    G:0.34641

#9: CGL
position  1:    T:0.27660    C:0.27660    A:0.42553    G:0.02128
position  2:    T:0.06383    C:0.06383    A:0.12766    G:0.74468
position  3:    T:0.06383    C:0.46809    A:0.21277    G:0.25532
Average         T:0.13475    C:0.26950    A:0.25532    G:0.34043

#10: ASA
position  1:    T:0.22449    C:0.28571    A:0.46939    G:0.02041
position  2:    T:0.04082    C:0.06122    A:0.12245    G:0.77551
position  3:    T:0.04082    C:0.46939    A:0.20408    G:0.28571
Average         T:0.10204    C:0.27211    A:0.26531    G:0.36054

#11: ATE
position  1:    T:0.22917    C:0.27083    A:0.47917    G:0.02083
position  2:    T:0.04167    C:0.06250    A:0.12500    G:0.77083
position  3:    T:0.04167    C:0.45833    A:0.20833    G:0.29167
Average         T:0.10417    C:0.26389    A:0.27083    G:0.36111

#12: CNI
position  1:    T:0.22449    C:0.30612    A:0.42857    G:0.04082
position  2:    T:0.04082    C:0.06122    A:0.12245    G:0.77551
position  3:    T:0.04082    C:0.46939    A:0.20408    G:0.28571
Average         T:0.10204    C:0.27891    A:0.25170    G:0.36735

#13: PCM
position  1:    T:0.22449    C:0.30612    A:0.42857    G:0.04082
position  2:    T:0.04082    C:0.06122    A:0.12245    G:0.77551
position  3:    T:0.04082    C:0.46939    A:0.20408    G:0.28571
Average         T:0.10204    C:0.27891    A:0.25170    G:0.36735

#14: MGE
```

```
position  1:    T:0.24490    C:0.24490    A:0.48980    G:0.02041
position  2:    T:0.04082    C:0.06122    A:0.12245    G:0.77551
position  3:    T:0.04082    C:0.46939    A:0.20408    G:0.28571
Average         T:0.10884    C:0.25850    A:0.27211    G:0.36054

#15: PDU
position  1:    T:0.24490    C:0.24490    A:0.48980    G:0.02041
position  2:    T:0.04082    C:0.06122    A:0.14286    G:0.75510
position  3:    T:0.04082    C:0.46939    A:0.20408    G:0.28571
Average         T:0.10884    C:0.25850    A:0.27891    G:0.35374

#16: PLU
position  1:    T:0.24490    C:0.24490    A:0.48980    G:0.02041
position  2:    T:0.04082    C:0.06122    A:0.12245    G:0.77551
position  3:    T:0.04082    C:0.46939    A:0.20408    G:0.28571
Average         T:0.10884    C:0.25850    A:0.27211    G:0.36054

#17: MCA
position  1:    T:0.24490    C:0.26531    A:0.46939    G:0.02041
position  2:    T:0.04082    C:0.06122    A:0.16327    G:0.73469
position  3:    T:0.06122    C:0.46939    A:0.20408    G:0.26531
Average         T:0.11565    C:0.26531    A:0.27891    G:0.34014

Sums of codon usage counts
------------------------------------------------------------------------------
Phe F TTT       0 | Ser S TCT       0 | Tyr Y TAT       0 | Cys C TGT      36
      TTC       0 |       TCC       2 |       TAC      62 |       TGC      95
Leu L TTA       0 |       TCA       6 | *** * TAA       0 | *** * TGA       0
      TTG       0 |       TCG       0 |       TAG       0 | Trp W TGG       0
------------------------------------------------------------------------------
Leu L CTT       0 | Pro P CCT       0 | His H CAT       0 | Arg R CGT       6
      CTC       0 |       CCC       0 |       CAC       1 |       CGC     133
      CTA       3 |       CCA       0 | Gln Q CAA       2 |       CGA      45
      CTG       4 |       CCG       0 |       CAG       1 |       CGG      44
------------------------------------------------------------------------------
Ile I ATT       0 | Thr T ACT       1 | Asn N AAT       0 | Ser S AGT       1
      ATC       0 |       ACC      14 |       AAC       0 |       AGC      58
      ATA       2 |       ACA       9 | Lys K AAA      34 | Arg R AGA      84
Met M ATG      25 |       ACG       0 |       AAG       1 |       AGG     148
------------------------------------------------------------------------------
Val V GTT       0 | Ala A GCT       0 | Asp D GAT       0 | Gly G GGT       0
      GTC       0 |       GCC      17 |       GAC       0 |       GGC       3
      GTA       0 |       GCA       0 | Glu E GAA       0 |       GGA       0
      GTG       1 |       GCG       0 |       GAG       0 |       GGG       0
------------------------------------------------------------------------------

(Ambiguity data are not used in the counts.)


Codon position x base (3x4) table, overall

position  1:    T:0.23986    C:0.28520    A:0.44988    G:0.02506
position  2:    T:0.04177    C:0.05847    A:0.12053    G:0.77924
position  3:    T:0.05251    C:0.45943    A:0.22076    G:0.26730
Average         T:0.11138    C:0.26770    A:0.26372    G:0.35720

Codon frequencies under model, for use in evolver (TTT TTC TTA TTG ... GGG):
  0.00000000  0.00000000  0.00000000  0.00000000
```

```
  0.00000000  0.00237129  0.00703089  0.00000000
  0.00000000  0.07351005  0.00000000  0.00000000
  0.04152249  0.11263637  0.00000000  0.00000000
  0.00000000  0.00000000  0.00351544  0.00461361
  0.00000000  0.00000000  0.00000000  0.00000000
  0.00000000  0.00119405  0.00234363  0.00115340
  0.00727578  0.16726607  0.05548489  0.05335568
  0.00000000  0.00000000  0.00234363  0.02883506
  0.00115340  0.01659904  0.01054633  0.00000000
  0.00000000  0.00000000  0.03984170  0.00115340
  0.00116125  0.06924847  0.09911285  0.17186489
  0.00000000  0.00000000  0.00000000  0.00115340
  0.00000000  0.02015598  0.00000000  0.00000000
  0.00000000  0.00000000  0.00000000  0.00000000
  0.00000000  0.00355694  0.00000000  0.00000000
```

```
Nei & Gojobori 1986. dN/dS (dN, dS)
(Pairwise deletion)
(Note: This matrix is not used in later ML. analysis.
Use runmode = -2 for ML pairwise comparison.)
```

```
MAR
MAG  0.2959 (0.0272 0.0919)
SHI  0.2052 (0.0464 0.2261) 0.2323 (0.0321 0.1382)
MMU  0.2470 (0.0608 0.2462) 0.1921 (0.0368 0.1914) 0.1242 (0.0177 0.1424)
MAU  0.2417 (0.0370 0.1530) 0.2189 (0.0228 0.1044) 0.0799 (0.0088 0.1104) 0.1893 (0.0267 0.1413)
PSU  0.5087 (0.0481 0.0946) 0.4577 (0.0284 0.0622) 0.1551 (0.0183 0.1182) 0.2389 (0.0323 0.1353) 0.0779 (0.0091 0.1173)
PRO  0.5106 (0.0482 0.0943) 0.4594 (0.0285 0.0620) 0.0994 (0.0183 0.1846) 0.2397 (0.0323 0.1349) 0.0781 (0.0091 0.1170) 0.3229 (0.0183 0.0568)
CGR  0.1749 (0.0464 0.2651) 0.1534 (0.0321 0.2092) 0.0854 (0.0177 0.2073) 0.1471 (0.0358 0.2432) 0.0429 (0.0088 0.2057) 0.1548 (0.0183 0.1184) 0.0992 (0.0183 0.1849)
CGL  0.7284 (0.0832 0.1142) 0.9067 (0.0576 0.0635) 0.3562 (0.0583 0.1636) 0.2981 (0.0383 0.1285) 0.5199 (0.0484 0.0931) 0.4180 (0.0347 0.0830) 0.6685 (0.0553 0.0828) 0.1903 (0.0383 0.2014)
ASA  0.7767 (0.0461 0.0593) 0.6171 (0.0180 0.0293) 0.2360 (0.0322 0.1366) 0.2396 (0.0369 0.1540) 0.3175 (0.0229 0.0722) 0.9495 (0.0286 0.0301) 0.9530 (0.0286 0.0300) 0.0798 (0.0136 0.1708) 1.2388 (0.0381 0.0307)
ATE  0.7673 (0.0469 0.0611) 0.6096 (0.0184 0.0301) 0.2329 (0.0328 0.1409) 0.3021 (0.0376 0.1244) 0.3139 (0.0234 0.0744) 0.9382 (0.0291 0.0310) 0.9417 (0.0291 0.0309) 0.0787 (0.0139 0.1764)-1.0000 (0.0388 0.0000)-1.0000 (0.0000 0.0000)
CNI  1.1317 (0.0657 0.0581) 1.6143 (0.0463 0.0287) 0.2477 (0.0372 0.1501) 0.3659 (0.0613 0.1676) 0.3239 (0.0278 0.0858) 1.3102 (0.0386 0.0294) 1.3148 (0.0386 0.0294) 0.0996 (0.0184 0.1844) 1.3891 (0.0633 0.0456)-1.0000 (0.0275 0.0000)-1.0000 (0.0280 0.0000)
PCM  1.1317 (0.0657 0.0581) 1.6143 (0.0463 0.0287) 0.2477 (0.0372 0.1501) 0.3659 (0.0613 0.1676) 0.3239 (0.0278 0.0858) 1.3102 (0.0386 0.0294) 1.3148 (0.0386 0.0294) 0.0996 (0.0184 0.1844) 1.3891 (0.0633 0.0456)-1.0000 (0.0275 0.0000)-1.0000 (0.0280 0.0000)-1.0000 (0.0000 0.0000)
MGE  1.5471 (0.0458 0.0296) 0.2951 (0.0179 0.0608) 0.2931 (0.0510 0.1740) 0.1903 (0.0367 0.1928) 0.3954 (0.0416 0.1051) 0.6064 (0.0381 0.0628) 0.6088 (0.0381 0.0626) 0.1520 (0.0320 0.2107) 0.5910 (0.0378 0.0640) 0.6117 (0.0180 0.0294) 0.6041 (0.0183 0.0304) 1.6005 (0.0462 0.0288) 1.6005 (0.0462 0.0288)
PDU  1.8426 (0.0551 0.0299) 0.4392 (0.0270 0.0615) 0.3435 (0.0604 0.1760) 0.2358 (0.0460 0.1951) 0.4796 (0.0509 0.1062) 0.7520 (0.0477 0.0635) 0.7549 (0.0478 0.0633) 0.1937 (0.0413 0.2132) 0.7326 (0.0474 0.0648) 0.9106 (0.0271 0.0298) 0.8991 (0.0276 0.0307) 1.9068 (0.0556 0.0291) 1.9068 (0.0556 0.0291)-1.0000 (0.0089 0.0000)
PLU  1.5471 (0.0458 0.0296) 0.2951 (0.0179 0.0608) 0.2931 (0.0510 0.1740) 0.1903 (0.0367 0.1928) 0.3954 (0.0416 0.1051) 0.6064 (0.0381 0.0628) 0.6088 (0.0381 0.0626) 0.1520 (0.0320 0.2107) 0.5910 (0.0378 0.0640) 0.6117 (0.0180 0.0294) 0.6041 (0.0183 0.0304) 1.6005 (0.0462 0.0288) 1.6005 (0.0462 0.0288)-1.0000 (0.0000 0.0000)-1.0000 (0.0089 0.0000)
MCA  0.7923 (0.0744 0.0939) 1.0465 (0.0645 0.0617) 0.5673 (0.1001 0.1765) 0.6810 (0.0849 0.1246) 0.8470 (0.0902 0.1065) 1.3846 (0.0881 0.0637) 1.3900 (0.0882 0.0635) 0.3739 (0.0800 0.2139) 1.3486 (0.0876 0.0650) 2.1703 (0.0648 0.0298) 2.1436 (0.0660 0.0308) 3.2496 (0.0950 0.0292) 3.2496 (0.0950 0.0292) 0.7314 (0.0454 0.0621) 0.8705 (0.0547 0.0628) 0.7314
(0.0454 0.0621)
```

```
TREE #  1:  (4, (3, ((((6, 13), 7), (5, 8)), ((10, 11), (9, (12, (17, ((2, 1), (14, (15, 16))))))))));   MP score: -1
This is a rooted tree.  Please check!
lnL(ntime: 32  np: 37):   -474.830556      +0.000000
  18..4    18..19   19..3    19..20   20..21   21..22   22..23   23..6    23..13   22..7    21..24   24..5    24..8    20..25   25..26   26..10   26..11   25..27   27..9    27..28   28..12   28..29   29..17   29..30   30..31
31..2    31..1    30..32   32..14   32..33   33..15   33..16
 0.059598 0.057709 0.093223 0.129838 0.027068 0.000004 0.000004 0.081958 0.057421 0.116827 0.031363 0.074864 0.150471 0.000004 0.000004 0.000004 0.000004 0.000004 0.145277 0.000004 0.084345 0.027080 0.204942 0.000004 0.000004
0.048501 0.172945 0.055220 0.000004 0.000004 0.027589 0.000004 2.155747 0.397201 0.489333 0.000001 9.962781

Note: Branch length is defined as number of nucleotide substitutions per codon (not per neucleotide site).

tree length =   1.64629

(4: 0.059598, (3: 0.093223, ((((6: 0.081958, 13: 0.057421): 0.000004, 7: 0.116827): 0.000004, (5: 0.074864, 8: 0.150471): 0.031363): 0.027068, ((10: 0.000004, 11: 0.000004): 0.000004, (9: 0.145277, (12: 0.084345, (17: 0.204942,
((2: 0.048501, 1: 0.172945): 0.000004, (14: 0.000004, (15: 0.027589, 16: 0.000004): 0.000004): 0.055220): 0.000004): 0.027080): 0.000004): 0.000004): 0.000004): 0.129838): 0.057709);

(MMU: 0.059598, (SHI: 0.093223, ((((PSU: 0.081958, PCM: 0.057421): 0.000004, PRO: 0.116827): 0.000004, (MAU: 0.074864, CGR: 0.150471): 0.031363): 0.027068, ((ASA: 0.000004, ATE: 0.000004): 0.000004, (CGL: 0.145277, (CNI: 0.084345,
(MCA: 0.204942, ((MAG: 0.048501, MAR: 0.172945): 0.000004, (MGE: 0.000004, (PDU: 0.027589, PLU: 0.000004): 0.000004): 0.055220): 0.000004): 0.027080): 0.000004): 0.000004): 0.000004): 0.129838): 0.057709);

Detailed output identifying parameters
```

kappa (ts/tv) = 2.15575

dN/dS (w) for site classes (K=3)

p: 0.39720 0.48933 0.11347
w: 0.00000 1.00000 9.96278

dN & dS for each branch

branch	t	N	S	dN/dS	dN	dS	N*dN	S*dS
18..4	0.060	87.2	65.8	1.6198	0.0238	0.0147	2.1	1.0
18..19	0.058	87.2	65.8	1.6198	0.0230	0.0142	2.0	0.9
19..3	0.093	87.2	65.8	1.6198	0.0372	0.0230	3.2	1.5
19..20	0.130	87.2	65.8	1.6198	0.0518	0.0320	4.5	2.1
20..21	0.027	87.2	65.8	1.6198	0.0108	0.0067	0.9	0.4
21..22	0.000	87.2	65.8	1.6198	0.0000	0.0000	0.0	0.0
22..23	0.000	87.2	65.8	1.6198	0.0000	0.0000	0.0	0.0
23..6	0.082	87.2	65.8	1.6198	0.0327	0.0202	2.9	1.3
23..13	0.057	87.2	65.8	1.6198	0.0229	0.0141	2.0	0.9
22..7	0.117	87.2	65.8	1.6198	0.0466	0.0288	4.1	1.9
21..24	0.031	87.2	65.8	1.6198	0.0125	0.0077	1.1	0.5
24..5	0.075	87.2	65.8	1.6198	0.0299	0.0184	2.6	1.2
24..8	0.150	87.2	65.8	1.6198	0.0600	0.0371	5.2	2.4
20..25	0.000	87.2	65.8	1.6198	0.0000	0.0000	0.0	0.0
25..26	0.000	87.2	65.8	1.6198	0.0000	0.0000	0.0	0.0
26..10	0.000	87.2	65.8	1.6198	0.0000	0.0000	0.0	0.0
26..11	0.000	87.2	65.8	1.6198	0.0000	0.0000	0.0	0.0
25..27	0.000	87.2	65.8	1.6198	0.0000	0.0000	0.0	0.0
27..9	0.145	87.2	65.8	1.6198	0.0580	0.0358	5.1	2.4
27..28	0.000	87.2	65.8	1.6198	0.0000	0.0000	0.0	0.0
28..12	0.084	87.2	65.8	1.6198	0.0336	0.0208	2.9	1.4
28..29	0.027	87.2	65.8	1.6198	0.0108	0.0067	0.9	0.4
29..17	0.205	87.2	65.8	1.6198	0.0818	0.0505	7.1	3.3
29..30	0.000	87.2	65.8	1.6198	0.0000	0.0000	0.0	0.0
30..31	0.000	87.2	65.8	1.6198	0.0000	0.0000	0.0	0.0
31..2	0.049	87.2	65.8	1.6198	0.0193	0.0119	1.7	0.8
31..1	0.173	87.2	65.8	1.6198	0.0690	0.0426	6.0	2.8
30..32	0.055	87.2	65.8	1.6198	0.0220	0.0136	1.9	0.9
32..14	0.000	87.2	65.8	1.6198	0.0000	0.0000	0.0	0.0
32..33	0.000	87.2	65.8	1.6198	0.0000	0.0000	0.0	0.0
33..15	0.028	87.2	65.8	1.6198	0.0110	0.0068	1.0	0.4
33..16	0.000	87.2	65.8	1.6198	0.0000	0.0000	0.0	0.0

Naive Empirical Bayes (NEB) analysis
Positively selected sites (*: P>95%; **: P>99%)
(amino acids refer to 1st sequence: MAR)

	Pr(w>1)	post mean +- SE for w
31 R	0.619	6.549
38 C	0.995**	9.914
43 T	0.993**	9.902
45 T	0.567	6.081
46 V	1.000**	9.961

```
Bayes Empirical Bayes (BEB) analysis (Yang, Wong & Nielsen 2005. Mol. Biol. Evol. 22:1107-1118)
Positively selected sites (*: P>95%; **: P>99%)
(amino acids refer to 1st sequence: MAR)

            Pr(w>1)     post mean +- SE for w

    31 R      0.634         5.453 +- 3.799
    38 C      0.996**       8.158 +- 1.837
    43 T      0.993**       8.140 +- 1.874
    45 T      0.677         5.713 +- 3.630
    46 V      1.000**       8.189 +- 1.782



The grid (see ternary graph for p0-p1)

w0:   0.050  0.150  0.250  0.350  0.450  0.550  0.650  0.750  0.850  0.950
w2:   1.500  2.500  3.500  4.500  5.500  6.500  7.500  8.500  9.500 10.500


Posterior on the grid

w0:   0.308  0.225  0.168  0.123  0.083  0.049  0.025  0.012  0.005  0.002
w2:   0.000  0.001  0.011  0.038  0.081  0.129  0.168  0.191  0.196  0.186

Posterior for p0-p1 (see the ternary graph)

 0.000
 0.001 0.001 0.001
 0.000 0.001 0.005 0.006 0.003
 0.000 0.000 0.001 0.007 0.019 0.017 0.007
 0.000 0.000 0.000 0.001 0.007 0.023 0.046 0.031 0.009
 0.000 0.000 0.000 0.000 0.001 0.006 0.018 0.047 0.069 0.038 0.008
 0.000 0.000 0.000 0.000 0.000 0.001 0.003 0.014 0.029 0.062 0.067 0.033 0.006
 0.000 0.000 0.000 0.000 0.000 0.000 0.000 0.002 0.006 0.019 0.029 0.053 0.047 0.022 0.004
 0.000 0.000 0.000 0.000 0.000 0.000 0.000 0.000 0.001 0.004 0.006 0.017 0.021 0.036 0.031 0.014 0.002
 0.000 0.000 0.000 0.000 0.000 0.000 0.000 0.000 0.000 0.001 0.001 0.003 0.005 0.012 0.014 0.024 0.022 0.010 0.002

sum of density on p0-p1 =   1.000000

Time used:  2:00
```
