# Supplementary material for: Sexual Selection Halts the Relaxation of Protamine 2 among Rodents
Source: PLoS One. 2011 Dec 21;6(12):e29247. doi: 10.1371/journal.pone.0029247 (PMC3244444; doi:10.1371/journal.pone.0029247)
Supplement: Figure S7 — Codeml output file (M2) for cleaved-Protamine 2. Included species of Cricetidae represented by abbreviated code: Arvicola sadipus (ASA), Arvicola terrestris (ATE), Clethrionomys glareolus (CGL), Cricetelus griseus (CGR), Chionomys nivalis (CNI), Microtus agrestis (MAG), Microtus arvalis (MAR), Mesocricetus auratus (MAU), Microtus cabrerae (MCA), Microtus gerbei (MGE), Mus musculus musculus (MMU), Phodopus campbelli (PCM), Pitymys duodecimcostatus (PDU), Pitymys lusitanicus (PLU), Phodopus roborovskii (PRO), Phodopus sungorus (PSU), Sigmodon hispidus (SHI). (PDF) [file pone.0029247.s007.pdf]

Figure S7. Cleaved Protamine 2
Codeml output file M2

Arvicola sadipus (ASA)
Arvicola terrestris (ATE)
Clethrionomys glareolus (CGL)
Cricetelus griseus (CGR)
Chionomys nivalis (CNI)
Microtus agrestis (MAG)
Microtus arvalis (MAR)
Mesocricetus auratus (MAU)
Microtus cabrerae (MCA)
Microtus gerbei (MGE)
Mus musculus musculus (MMU)
Phodopus campbelli (PCM)
Pitymys duodecimcostatus (PDU)
Pitymys lusitanicus (PLU)
Phodopus roborovskii (PRO)
Phodopus sugorus (PSU)
Sigmodon hispidus (SHI)

```
     16    165

SHI  ATG GTT CGC TAC CGA ACA AGG AGC CCC AGT GAG CAT CTG CAC CAG GGG CCT GGG CAA GAA CAT GGA CGG G-- --- -AA GAA CAG GGG CAA GGG CTA AGC CCA GAG CGC GTG GAG GAC TAT GGG AGG ACA CAC AGG GGC CAC CAC CAC CAC AGA CAC CGT CGC TGC
ASA  ATG GTT CGC TAC CGA ATG AGG AGC CCC AGT GAG GGT CCA CAC CAG GGG CCT GGG CAA GAC CAT GGA CCC GAC GAG CAG GGG CAG GGG CAA GGG CTG AGC CCA GAG CGT GTG GAG GAC TAT GGG AGG ACA CAC AGG GGT CAC CAC CAT CAC AGA C-- -GG CGC TGC
ATE  ATG GTT CGC TAC CGA ATG AGG AGC CCC AGT GAG CAT CCA CAC CAG GGG CCT GGG CAA GAC CAT GGA CCC GAC GAG CAG GGG CAG GGG CAA GGG YTG AGC CCA GAG CGT GTG GAG GAC TAT GGG AGG ACA CAC AGG GGT CAC CAC CAT CAC AGA C-- -GG CGC TGC
CGL  ATG GTT CGC TAC CGA ATA AGG AGC CCC AGT GAG CAT CCA CAC CAG GGG CCT GGG CAA GAC GAT GGA CAT AAA GAC CAG GGG CAG AGG CAA GGG CTG AGC CCA GAG CGT GTG GAG GAC TAT GGG AGG ACA CAT AGG GGT CAC CAC CAT CAC AGA C-- -GG CGC TGC
CNI  ATG GTT CGG TAC CGA GTG AGG AGC CCC AGT GAG CAT CCA CAC CAG GGG CCT GGG CAA GAC GAT GGA CAC GAC GAG CAG GGG CAG GGG CAA GGG CTG AGT CCA GAG CGT GTG GAT GAC TAT GGG AGG ACA CAC AGG GGT CAC CAC CAT CAT AGA C-- -GG CGC TGC
MAR  ATG GTT CGT TAC CGA ATG AGG AGC CCC AGT GAG CAT CCA CAC CAG GGG CCT GGG CAA GAC GAT GGA CAC GAC GAG CAG GGG CA- --- --A GGG CTG AGT CCA GAG CGT GTG GAG GAC TTG GGG AGG ACA CAC AGG GGT TAC CAC CAT CAC AGA C-- -GG CGC TGC
MCA  ATG GTT CGT TAC CGA ATG AGG AGC CCC AGT GAG CAT CCA CAC CAG GGG CCT GGG CAA GAC TAT GGA CAC AAC GAG CAG GGG CAG G-- --- -GG CTG AGT CCA GAG CGT GTG GAG GAT TAT GGG AGG ACA CAC AGG GGT TAC CAC CAT CAC AGA C-- -GG CGC TGC
MGE  ATG GTT CGT TAC CGA ATG AGG AGT CCC AGT GAG CAT CCA CAC CAG GGG CCT GGG CAA GAC AAT AGA CAC RAC GAG CAG GGG CAG GGG CAA GGG CTG AGT CCA GAG CGT GTG GAG GAC TAT GGG AGG ACA CAC AGG GGT TAC CAC CAT CAC AGA C-- -GG CGC TGC
MAG  ATG GTT CGT TAC CGA ATG AGG AGC CCC AGT GAG CAT CCA CGC CAG GGG CCT GGG CAA GAC GAT GGA CGT GAC GAG CAG GGG CAG GGG CAA GGG CTG AGT CCA GAG CGT GTG GAG GAC TAT GGG AGG ACA CAC AGG GGT TAC CAC CAT CAC AGA C-- -GG CGC TGC
PDU  ATG GTT CGT TAC CGA ATG AGG AGT CCC AGT GAG CAT CCA CAC CAG GGG CCT GGG CAA GAC AAT AGA CAC GAC GAG CAG GGG CAG GGG CAA GGG CTG AGT CCA GAG TGT GTG GAG GAC TAT GGG AGG ACA CAC AGG GGT TAC CAC CAT CAC AGA C-- -GG CGC TGC
PLU  ATG GTT CGT TAC CGA ATG AGG AGT CCC AGT GAG CAT CCA CAC CAG GGG CCT GGG CAA GAC AAT AGA CAC GAC GAA CAG GGG CAG GGG CAA GGG CTG AGT CCA GAG CGT GTG GAR GAC TAT GGG AGG ACA CAC AGG GGT TAC CAY CAT CAC AGA C-- -GG CGC TGC
MAU  ATG GTT CGT TAC CGA ATG AGG AGC CCC AGT GAG CGT CCG CAC CAG GGG CCT GGG CAA GAA CAT GGA CGT GAA GAG CAA GGG CAG GGG CAA GGG TTG AGC CCA GAG CGT GTG GAG GAC TAT GGG AGG ACA CAC AGG GGC CAA CAC CAC CAC AGA CGT CGC TGC ---
PSU  ATG GTT CGT TAC CGA ATG AGA AGC CCC AGT GAG CGT CCA CAC CAG GGG CAC GGG CAA GAA CAT G-- --- --- --- -AA GAG CAG GGG CAA GGG TTC AGC CCA GAG CAT GTG GAG GAC TAT GGG AGG ACA CAT AGG TGC CAC CAC CAC CAC AGA CAC CGT CGC TGC
PRO  ATG GTT CGT TAC CGA GTG AGA AGC CCC AGT GAG CGT CCA CGC AAG GGG CCT GGG CAA GAA CAT G-- --- --- --- -AA GAT CAG GGG CAA GGG TTG AGC CCA GAG CAT GTG GAG GAC TAT GGG AGG ACA CAT AGG GGC CAC CAC CAT CAC AGA CAC CGT CGC TGC
PCM  ATG GTT CGT TAC CGA ATG AGA AGC CCC AGT GAG CGT CCA CAC CAG GGG CAC GGG CAA GAA CAT G-- --- --- --- -AA GAG CAG GGG CAA GGG TTC AGC CCA GAG CAT GTG GAG GAC TAT GGG AGG ACA CAT AGG TGC CAC CAC CAC CAC AGA CAC CGT CGC TGC
MMU  ATG GTT CGC TAC CGA ATG AGG AGC CCC AGT GAG GGT CCG CAC CAG GGG CCT GGA CAA GAC CAT GAA CGC GAG GAG CAG GGG CAG GGG CAA GGG CTG AGC CCA GAG CGC GTA GAG GAC TAT GGG AGG ACA CAC AGG GGC CAC CAC CAC CAC AGA CAC AGG CGC TGC
```

Printing out site pattern counts

```
        16        156  P

SHI  --- -AA ACA ACA AGA AGC AGC AGG AGG AGT ATG CAA CAA CAC CAC CAC CAC CAC CAC CAC CAG CAG CAT CAT CCA CCC CCT CGA CGC CGC CGC CGG CGT CTA CTG G-- GAA GAA GAC GAG GAG GGA GGC GGG GGG GGG GGG GTG GTT TAC TAT TGC
ASA  GAG CAG ACA ATG AGA AGC AGC AGG AGG AGT ATG CAA CAA C-- CAC CAC CAC CAC CAC CAT CAG CAG CAT GGT CCA CCC CCT CGA CGC CGC CGT CCC -GG CTG CCA GAC GAC GGG GAC GAG GAG GGA GGT GGG GGG GGG GGG GTG GTT TAC TAT TGC
ATE  GAG CAG ACA ATG AGA AGC AGC AGG AGG AGT ATG CAA CAA C-- CAC CAC CAC CAC CAC CAT CAG CAG CAT CAT CCA CCC CCT CGA CGC CGC CGT CCC -GG YTG CCA GAC GAC GGG GAC GAG GAG GGA GGT GGG GGG GGG GGG GTG GTT TAC TAT TGC
CGL  GAC CAG ACA ATA AGA AGC AGC AGG AGG AGT ATG CAA CAA C-- CAC CAC CAC CAC CAT CAT CAG CAG GAT CAT CCA CCC CCT CGA CGC CGC CGT CAT -GG CTG CCA AAA GAC GGG GAC GAG GAG GGA GGT AGG GGG GGG GGG GTG GTT TAC TAT TGC
CNI  GAG CAG ACA GTG AGA AGC AGT AGG AGG AGT ATG CAA CAA C-- CAC CAC CAC CAT CAC CAT CAG CAG GAT CAT CCA CCC CCT CGA CGC CGG CGT CAC -GG CTG CCA GAC GAC GGG GAC GAG GAT GGA GGT GGG GGG GGG GGG GTG GTT TAC TAT TGC
```

```
MAR  GAG CAG ACA ATG AGA AGC AGT AGG AGG AGT ATG --A CAA C-- CAC CAC TAC CAC CAC CAT CA- CAG GAT CAT CCA CCC CCT CGA CGC CGT CGT CAC -GG CTG CCA GAC GAC GGG GAC GAG GAG GGA GGT --- GGG GGG GGG GTG GTT TAC TTG TGC
MCA  GAG CAG ACA ATG AGA AGC AGT AGG AGG AGT ATG --- CAA C-- CAC CAC TAC CAC CAC CAT CAG CAG TAT CAT CCA CCC CCT CGA CGC CGT CGT CAC -GG CTG CCA AAC GAC GGG GAT GAG GAG GGA GGT G-- -GG GGG GGG GTG GTT TAC TAT TGC
MGE  GAG CAG ACA ATG AGA AGT AGT AGG AGG AGT ATG CAA CAA C-- CAC CAC TAC CAC CAC CAT CAG CAG AAT CAT CCA CCC CCT CGA CGC CGT CGT CAC -GG CTG CCA RAC GAC GGG GAC GAG GAG AGA GGT GGG GGG GGG GGG GTG GTT TAC TAT TGC
MAG  GAG CAG ACA ATG AGA AGC AGT AGG AGG AGT ATG CAA CAA C-- CAC CGC TAC CAC CAC CAT CAG CAG GAT CAT CCA CCC CCT CGA CGC CGT CGT CGT -GG CTG CCA GAC GAC GGG GAC GAG GAG GGA GGT GGG GGG GGG GGG GTG GTT TAC TAT TGC
PDU  GAG CAG ACA ATG AGA AGT AGT AGG AGG AGT ATG CAA CAA C-- CAC CAC TAC CAC CAC CAT CAG CAG AAT CAT CCA CCC CCT CGA CGC CGT TGT CAC -GG CTG CCA GAC GAC GGG GAC GAG GAG AGA GGT GGG GGG GGG GGG GTG GTT TAC TAT TGC
PLU  GAA CAG ACA ATG AGA AGT AGT AGG AGG AGT ATG CAA CAA C-- CAY CAC TAC CAC CAC CAT CAG CAG AAT CAT CCA CCC CCT CGA CGC CGT CGT CAC -GG CTG CCA GAC GAC GGG GAC GAG GAR AGA GGT GGG GGG GGG GGG GTG GTT TAC TAT TGC
MAU  GAG CAA ACA ATG AGA AGC AGC AGG AGG AGT ATG CAA CAA CGT CAC CAC CAA CAC CAC CAC CAG CAG CAT CGT CCA CCC CCT CGA TGC CGT CGT CGT CGC TTG CCG GAA GAA GGG GAC GAG GAG GGA GGC GGG GGG GGG GGG GTG GTT TAC TAT ---
PSU  --- -AA ACA ATG AGA AGC AGC AGA AGG AGT ATG CAA CAA CAC CAC CAC CAC CAC CAT CAC CAG CAG CAT CGT CCA CCC CAC CGA CGC CGT CAT --- CGT TTC CCA --- GAA GAG GAC GAG GAG G-- TGC GGG GGG GGG GGG GTG GTT TAC TAT TGC
PRO  --- -AA ACA GTG AGA AGC AGC AGA AGG AGT ATG CAA CAA CAC CAC CGC CAC CAC CAT CAT CAG AAG CAT CGT CCA CCC CCT CGA CGC CGT CAT --- CGT TTG CCA --- GAA GAT GAC GAG GAG G-- GGC GGG GGG GGG GGG GTG GTT TAC TAT TGC
PCM  --- -AA ACA ATG AGA AGC AGC AGA AGG AGT ATG CAA CAA CAC CAC CAC CAC CAC CAT CAC CAG CAG CAT CGT CCA CCC CAC CGA CGC CGT CAT --- CGT TTC CCA --- GAA GAG GAC GAG GAG G-- TGC GGG GGG GGG GGG GTG GTT TAC TAT TGC
MMU  GAG CAG ACA ATG AGA AGC AGC AGG AGG AGT ATG CAA CAA CAC CAC CAC CAC CAC CAC CAC CAG CAG CAT GGT CCA CCC CCT CGA CGC CGC CGC CGC AGG CTG CCG GAG GAC GGG GAC GAG GAG GAA GGC GGG GGG GGA GGG GTA GTT TAC TAT TGC



    1    1    1    1    1    1    1    1    2    1    1    1    1    1    1
    1    1    1    1    1    1    1    1    1    1    1    1    1    1    1
    1    1    1    1    1    1    1    1    1    2    1    1    1    1    1
    1    2    1    1    1    1    1

CODONML (in paml version 4.4d, March 2011)  1.phy
Model: One dN/dS ratio for branches
Codon frequency model: Fcodon
Site-class models:  PositiveSelection
ns =  16  ls =  55

Codon usage in sequences
--------------------------------------------------------------------------------------------------------------
Phe TTT  0  0  0  0  0  0 | Ser TCT  0  0  0  0  0  0 | Tyr TAT  1  1  1  1  1  0 | Cys TGT  0  0  0  0  0  0
    TTC  0  0  0  0  0  0 |     TCC  0  0  0  0  0  0 |     TAC  1  1  1  1  1  2 |     TGC  1  1  1  1  1  1
Leu TTA  0  0  0  0  0  0 |     TCA  0  0  0  0  0  0 | *** TAA  0  0  0  0  0  0 | *** TGA  0  0  0  0  0  0
    TTG  0  0  0  0  0  1 |     TCG  0  0  0  0  0  0 |     TAG  0  0  0  0  0  0 | Trp TGG  0  0  0  0  0  0
--------------------------------------------------------------------------------------------------------------
Leu CTT  0  0  0  0  0  0 | Pro CCT  1  1  1  1  1  1 | His CAT  2  2  3  4  3  2 | Arg CGT  1  1  1  1  1  2
    CTC  0  0  0  0  0  0 |     CCC  1  2  2  1  1  1 |     CAC  7  5  5  4  5  5 |     CGC  3  2  2  2  1  1
    CTA  1  0  0  0  0  0 |     CCA  1  2  2  2  2  2 | Gln CAA  2  2  2  2  2  1 |     CGA  1  1  1  1  1  1
    CTG  1  1  0  1  1  1 |     CCG  0  0  0  0  0  0 |     CAG  2  3  3  3  3  2 |     CGG  1  0  0  0  1  0
--------------------------------------------------------------------------------------------------------------
Ile ATT  0  0  0  0  0  0 | Thr ACT  0  0  0  0  0  0 | Asn AAT  0  0  0  0  0  0 | Ser AGT  1  1  1  1  2  2
    ATC  0  0  0  0  0  0 |     ACC  0  0  0  0  0  0 |     AAC  0  0  0  0  0  0 |     AGC  2  2  2  2  1  1
    ATA  0  0  0  1  0  0 |     ACA  2  1  1  1  1  1 | Lys AAA  0  0  0  1  0  0 | Arg AGA  1  1  1  1  1  1
Met ATG  1  2  2  1  1  2 |     ACG  0  0  0  0  0  0 |     AAG  0  0  0  0  0  0 |     AGG  3  3  3  4  3  3
--------------------------------------------------------------------------------------------------------------
Val GTT  1  1  1  1  1  1 | Ala GCT  0  0  0  0  0  0 | Asp GAT  0  0  0  1  2  1 | Gly GGT  0  2  1  1  1  1
    GTC  0  0  0  0  0  0 |     GCC  0  0  0  0  0  0 |     GAC  1  3  3  3  3  3 |     GGC  1  0  0  0  0  0
    GTA  0  0  0  0  0  0 |     GCA  0  0  0  0  0  0 | Glu GAA  2  0  0  0  0  0 |     GGA  1  1  1  1  1  1
    GTG  1  1  1  1  2  1 |     GCG  0  0  0  0  0  0 |     GAG  3  4  4  3  3  4 |     GGG  5  6  6  5  6  5
--------------------------------------------------------------------------------------------------------------


--------------------------------------------------------------------------------------------------------------
Phe TTT  0  0  0  0  0  0 | Ser TCT  0  0  0  0  0  0 | Tyr TAT  2  1  1  1  1  1 | Cys TGT  0  0  0  1  0  0
    TTC  0  0  0  0  0  0 |     TCC  0  0  0  0  0  0 |     TAC  2  2  2  2  2  1 |     TGC  1  1  1  1  1  1
Leu TTA  0  0  0  0  0  0 |     TCA  0  0  0  0  0  0 | *** TAA  0  0  0  0  0  0 | *** TGA  0  0  0  0  0  0
    TTG  0  0  0  0  0  1 |     TCG  0  0  0  0  0  0 |     TAG  0  0  0  0  0  0 | Trp TGG  0  0  0  0  0  0
--------------------------------------------------------------------------------------------------------------
Leu CTT  0  0  0  0  0  0 | Pro CCT  1  1  1  1  1  1 | His CAT  2  2  2  2  2  1 | Arg CGT  2  2  3  1  2  5
    CTC  0  0  0  0  0  0 |     CCC  1  1  1  1  1  1 |     CAC  5  5  3  5  4  5 |     CGC  1  1  2  1  1  1
    CTA  0  0  0  0  0  0 |     CCA  2  2  2  2  2  1 | Gln CAA  1  2  2  2  2  4 |     CGA  1  1  1  1  1  1
    CTG  1  1  1  1  1  0 |     CCG  0  0  0  0  0  1 |     CAG  3  3  3  3  3  2 |     CGG  0  0  0  0  0  0
```

```
------------------------------------------------------------------------------------------------------------------
Ile ATT  0  0  0  0  0  0 | Thr ACT  0  0  0  0  0  0 | Asn AAT  0  1  0  1  1  0 | Ser AGT  2  3  2  3  3  1
    ATC  0  0  0  0  0  0 |     ACC  0  0  0  0  0  0 |     AAC  1  0  0  0  0  0 |     AGC  1  0  1  0  0  2
    ATA  0  0  0  0  0  0 |     ACA  1  1  1  1  1  1 | Lys AAA  0  0  0  0  0  0 | Arg AGA  1  2  1  2  2  1
Met ATG  2  2  2  2  2  2 |     ACG  0  0  0  0  0  0 |     AAG  0  0  0  0  0  0 |     AGG  3  3  3  3  3  3
------------------------------------------------------------------------------------------------------------------
Val GTT  1  1  1  1  1  1 | Ala GCT  0  0  0  0  0  0 | Asp GAT  1  0  1  0  0  0 | Gly GGT  1  1  1  1  1  0
    GTC  0  0  0  0  0  0 |     GCC  0  0  0  0  0  0 |     GAC  1  2  3  3  3  1 |     GGC  0  0  0  0  0  1
    GTA  0  0  0  0  0  0 |     GCA  0  0  0  0  0  0 | Glu GAA  0  0  0  0  1  2 |     GGA  1  0  1  0  0  1
    GTG  1  1  1  1  1  1 |     GCG  0  0  0  0  0  0 |     GAG  4  4  4  4  2  4 |     GGG  4  6  6  6  6  6
------------------------------------------------------------------------------------------------------------------

----------------------------------------------------------------------------------------
Phe TTT  0  0  0  0 | Ser TCT  0  0  0  0 | Tyr TAT  1  1  1  1 | Cys TGT  0  0  0  0
    TTC  1  0  1  0 |     TCC  0  0  0  0 |     TAC  1  1  1  1 |     TGC  2  1  2  1
Leu TTA  0  0  0  0 |     TCA  0  0  0  0 | *** TAA  0  0  0  0 | *** TGA  0  0  0  0
    TTG  0  1  0  0 |     TCG  0  0  0  0 |     TAG  0  0  0  0 | Trp TGG  0  0  0  0
----------------------------------------------------------------------------------------
Leu CTT  0  0  0  0 | Pro CCT  0  1  0  1 | His CAT  3  4  3  1 | Arg CGT  3  3  3  0
    CTC  0  0  0  0 |     CCC  1  1  1  1 |     CAC  7  4  7  7 |     CGC  1  2  1  4
    CTA  0  0  0  0 |     CCA  2  2  2  1 | Gln CAA  2  2  2  2 |     CGA  1  1  1  1
    CTG  0  0  0  1 |     CCG  0  0  0  1 |     CAG  2  1  2  3 |     CGG  0  0  0  0
----------------------------------------------------------------------------------------
Ile ATT  0  0  0  0 | Thr ACT  0  0  0  0 | Asn AAT  0  0  0  0 | Ser AGT  1  1  1  1
    ATC  0  0  0  0 |     ACC  0  0  0  0 |     AAC  0  0  0  0 |     AGC  2  2  2  2
    ATA  0  0  0  0 |     ACA  1  1  1  1 | Lys AAA  0  0  0  0 | Arg AGA  2  2  2  1
Met ATG  2  1  2  2 |     ACG  0  0  0  0 |     AAG  0  1  0  0 |     AGG  2  2  2  4
----------------------------------------------------------------------------------------
Val GTT  1  1  1  1 | Ala GCT  0  0  0  0 | Asp GAT  0  1  0  0 | Gly GGT  0  0  0  1
    GTC  0  0  0  0 |     GCC  0  0  0  0 |     GAC  1  1  1  2 |     GGC  0  1  0  1
    GTA  0  0  0  1 |     GCA  0  0  0  0 | Glu GAA  1  1  1  1 |     GGA  0  0  0  1
    GTG  1  2  1  0 |     GCG  0  0  0  0 |     GAG  4  3  4  5 |     GGG  5  5  5  5
----------------------------------------------------------------------------------------

Codon position x base (3x4) table for each sequence.

#1: SHI
position  1:    T:0.05769    C:0.46154    A:0.19231    G:0.28846
position  2:    T:0.09615    C:0.09615    A:0.40385    G:0.40385
position  3:    T:0.13462    C:0.32692    A:0.21154    G:0.32692
Average         T:0.09615    C:0.29487    A:0.26923    G:0.33974

#2: ASA
position  1:    T:0.05660    C:0.41509    A:0.18868    G:0.33962
position  2:    T:0.09434    C:0.11321    A:0.39623    G:0.39623
position  3:    T:0.16981    C:0.30189    A:0.15094    G:0.37736
Average         T:0.10692    C:0.27673    A:0.24528    G:0.37107

#3: ATE
position  1:    T:0.05769    C:0.42308    A:0.19231    G:0.32692
position  2:    T:0.07692    C:0.11538    A:0.42308    G:0.38462
position  3:    T:0.17308    C:0.30769    A:0.15385    G:0.36538
Average         T:0.10256    C:0.28205    A:0.25641    G:0.35897

#4: CGL
position  1:    T:0.05660    C:0.41509    A:0.22642    G:0.30189
position  2:    T:0.09434    C:0.09434    A:0.43396    G:0.37736
position  3:    T:0.20755    C:0.26415    A:0.18868    G:0.33962
```

```
Average         T:0.11950    C:0.25786    A:0.28302    G:0.33962

#5: CNI
position  1:    T:0.05660    C:0.41509    A:0.16981    G:0.35849
position  2:    T:0.09434    C:0.09434    A:0.43396    G:0.37736
position  3:    T:0.22642    C:0.24528    A:0.15094    G:0.37736
Average         T:0.12579    C:0.25157    A:0.25157    G:0.37107

#6: MAR
position  1:    T:0.08000    C:0.38000    A:0.20000    G:0.34000
position  2:    T:0.12000    C:0.10000    A:0.40000    G:0.38000
position  3:    T:0.20000    C:0.28000    A:0.14000    G:0.38000
Average         T:0.13333    C:0.25333    A:0.24667    G:0.36667

#7: MCA
position  1:    T:0.10000    C:0.40000    A:0.22000    G:0.28000
position  2:    T:0.10000    C:0.10000    A:0.44000    G:0.36000
position  3:    T:0.24000    C:0.26000    A:0.14000    G:0.36000
Average         T:0.14667    C:0.25333    A:0.26667    G:0.33333

#8: MGE
position  1:    T:0.07692    C:0.40385    A:0.23077    G:0.28846
position  2:    T:0.09615    C:0.09615    A:0.42308    G:0.38462
position  3:    T:0.23077    C:0.23077    A:0.15385    G:0.38462
Average         T:0.13462    C:0.24359    A:0.26923    G:0.35256

#9: MAG
position  1:    T:0.07547    C:0.39623    A:0.18868    G:0.33962
position  2:    T:0.09434    C:0.09434    A:0.39623    G:0.41509
position  3:    T:0.22642    C:0.24528    A:0.15094    G:0.37736
Average         T:0.13208    C:0.24528    A:0.24528    G:0.37736

#10: PDU
position  1:    T:0.09434    C:0.37736    A:0.22642    G:0.30189
position  2:    T:0.09434    C:0.09434    A:0.43396    G:0.37736
position  3:    T:0.22642    C:0.24528    A:0.15094    G:0.37736
Average         T:0.13836    C:0.23899    A:0.27044    G:0.35220

#11: PLU
position  1:    T:0.07843    C:0.39216    A:0.23529    G:0.29412
position  2:    T:0.09804    C:0.09804    A:0.41176    G:0.39216
position  3:    T:0.23529    C:0.23529    A:0.17647    G:0.35294
Average         T:0.13725    C:0.24183    A:0.27451    G:0.34641

#12: MAU
position  1:    T:0.07407    C:0.42593    A:0.18519    G:0.31481
position  2:    T:0.09259    C:0.09259    A:0.38889    G:0.42593
position  3:    T:0.18519    C:0.24074    A:0.20370    G:0.37037
Average         T:0.11728    C:0.25309    A:0.25926    G:0.37037

#13: PSU
position  1:    T:0.10000    C:0.44000    A:0.20000    G:0.26000
position  2:    T:0.10000    C:0.08000    A:0.44000    G:0.38000
position  3:    T:0.18000    C:0.32000    A:0.18000    G:0.32000
Average         T:0.12667    C:0.28000    A:0.27333    G:0.32000

#14: PRO
position  1:    T:0.08000    C:0.42000    A:0.20000    G:0.30000
```

```
position  2:    T:0.10000    C:0.10000    A:0.40000    G:0.40000
position  3:    T:0.24000    C:0.26000    A:0.18000    G:0.32000
Average         T:0.14000    C:0.26000    A:0.26000    G:0.34000

#15: PCM
position  1:    T:0.10000    C:0.44000    A:0.20000    G:0.26000
position  2:    T:0.10000    C:0.08000    A:0.44000    G:0.38000
position  3:    T:0.18000    C:0.32000    A:0.18000    G:0.32000
Average         T:0.12667    C:0.28000    A:0.27333    G:0.32000

#16: MMU
position  1:    T:0.05455    C:0.41818    A:0.20000    G:0.32727
position  2:    T:0.09091    C:0.09091    A:0.41818    G:0.40000
position  3:    T:0.10909    C:0.34545    A:0.16364    G:0.38182
Average         T:0.08485    C:0.28485    A:0.26061    G:0.36970

Sums of codon usage counts
------------------------------------------------------------------------------
Phe F TTT       0 | Ser S TCT       0 | Tyr Y TAT      16 | Cys C TGT       1
      TTC       2 |       TCC       0 |       TAC      22 |       TGC      18
Leu L TTA       0 |       TCA       0 | *** * TAA       0 | *** * TGA       0
      TTG       3 |       TCG       0 |       TAG       0 | Trp W TGG       0
------------------------------------------------------------------------------
Leu L CTT       0 | Pro P CCT      14 | His H CAT      38 | Arg R CGT      31
      CTC       0 |       CCC      18 |       CAC      83 |       CGC      26
      CTA       1 |       CCA      29 | Gln Q CAA      32 |       CGA      16
      CTG      11 |       CCG       2 |       CAG      41 |       CGG       2
------------------------------------------------------------------------------
Ile I ATT       0 | Thr T ACT       0 | Asn N AAT       3 | Ser S AGT      26
      ATC       0 |       ACC       0 |       AAC       1 |       AGC      22
      ATA       1 |       ACA      17 | Lys K AAA       1 | Arg R AGA      22
Met M ATG      28 |       ACG       0 |       AAG       1 |       AGG      47
------------------------------------------------------------------------------
Val V GTT      16 | Ala A GCT       0 | Asp D GAT       7 | Gly G GGT      12
      GTC       0 |       GCC       0 |       GAC      34 |       GGC       4
      GTA       1 |       GCA       0 | Glu E GAA       9 |       GGA      10
      GTG      17 |       GCG       0 |       GAG      59 |       GGG      87
------------------------------------------------------------------------------

(Ambiguity data are not used in the counts.)


Codon position x base (3x4) table, overall

position  1:    T:0.07461    C:0.41396    A:0.20337    G:0.30806
position  2:    T:0.09627    C:0.09627    A:0.41757    G:0.38989
position  3:    T:0.19735    C:0.27677    A:0.16727    G:0.35860
Average         T:0.12274    C:0.26233    A:0.26274    G:0.35219

Codon frequencies under model, for use in evolver (TTT TTC TTA TTG ... GGG):
  0.00000000  0.00230681  0.00000000  0.00370157
  0.00000000  0.00000000  0.00000000  0.00000000
  0.01845444  0.02537486  0.00000000  0.00000000
  0.00115340  0.02076125  0.00000000  0.00000000
  0.00000000  0.00000000  0.00119466  0.01398739
  0.01660824  0.02135345  0.03464509  0.00237261
  0.04568127  0.09977750  0.04208581  0.04888368
  0.03677538  0.03084387  0.01911453  0.00256780
```

```
  0.00000000  0.00000000  0.00116130  0.03229527
  0.00000000  0.00000000  0.01974209  0.00000000
  0.00346021  0.00118575  0.00126803  0.00115340
  0.02998847  0.02537486  0.02554859  0.05853633
  0.01880125  0.00000000  0.00118328  0.01997633
  0.00000000  0.00000000  0.00000000  0.00000000
  0.00822555  0.04109478  0.01183489  0.07033544
  0.01410094  0.00470031  0.01183276  0.11055658
```

```
Nei & Gojobori 1986. dN/dS (dN, dS)
(Pairwise deletion)
(Note: This matrix is not used in later ML. analysis.
Use runmode = -2 for ML pairwise comparison.)

SHI
ASA  0.2593 (0.0676 0.2608)
ATE  0.2119 (0.0496 0.2340)-1.0000 (0.0165 0.0000)
CGL  0.2534 (0.0673 0.2657) 1.2547 (0.0759 0.0605) 0.9248 (0.0590 0.0638)
CNI  0.2020 (0.0787 0.3896) 0.5468 (0.0500 0.0914) 0.3454 (0.0333 0.0964) 0.3001 (0.0540 0.1801)
MAR  0.2742 (0.0976 0.3560) 0.9716 (0.0621 0.0640) 0.6558 (0.0444 0.0676) 0.4574 (0.0620 0.1354) 0.6847 (0.0438 0.0640)
MCA  0.1822 (0.0766 0.4202) 0.5245 (0.0526 0.1003) 0.3294 (0.0351 0.1064) 0.2445 (0.0435 0.1777) 0.4338 (0.0436 0.1005) 1.0736 (0.0353 0.0329)
MGE  0.2117 (0.0813 0.3843) 0.5376 (0.0508 0.0945) 0.3389 (0.0338 0.0998) 0.3011 (0.0505 0.1679) 0.4447 (0.0421 0.0946) 1.0936 (0.0355 0.0324) 0.2567 (0.0174 0.0679)
MAG  0.1945 (0.0677 0.3480) 0.5493 (0.0500 0.0911) 0.3470 (0.0333 0.0960) 0.7259 (0.0672 0.0925) 0.4545 (0.0414 0.0912) 1.1178 (0.0349 0.0312) 0.5320 (0.0347 0.0652) 0.5446 (0.0335 0.0615)
PDU  0.2332 (0.0907 0.3890) 0.6154 (0.0581 0.0944) 0.4156 (0.0415 0.0998) 0.4490 (0.0752 0.1676) 0.5237 (0.0495 0.0945) 1.3401 (0.0434 0.0324) 0.5062 (0.0343 0.0678)-1.0000 (0.0082 0.0000) 0.6680 (0.0411 0.0615)
PLU  0.2160 (0.0854 0.3954) 0.3980 (0.0519 0.1305) 0.2504 (0.0346 0.1382) 0.4105 (0.0697 0.1699) 0.2623 (0.0342 0.1305) 0.5402 (0.0363 0.0672) 0.2545 (0.0269 0.1057) 0.0000 (0.0000 0.0316) 0.3588 (0.0342 0.0954) 0.2635 (0.0083 0.0316)
MAU  0.2242 (0.0663 0.2956) 0.2114 (0.0509 0.2406) 0.2443 (0.0516 0.2111) 0.3527 (0.0864 0.2450) 0.2376 (0.0776 0.3268) 0.3528 (0.0922 0.2613) 0.2594 (0.0819 0.3158) 0.2369 (0.0697 0.2943) 0.3423 (0.0687 0.2006) 0.2926 (0.0860 0.2938) 0.2338 (0.0808 0.3456)
PSU  0.1963 (0.0660 0.3365) 0.2285 (0.0640 0.2802) 0.2268 (0.0555 0.2445) 0.3966 (0.0931 0.2347) 0.2467 (0.0935 0.3790) 0.3636 (0.1106 0.3041) 0.2386 (0.0886 0.3714) 0.2489 (0.0832 0.3342) 0.3340 (0.0933 0.2793) 0.2738 (0.0927 0.3387) 0.2537 (0.0874 0.3447) 0.3184 (0.0721 0.2266)
PRO  0.2218 (0.0733 0.3305) 0.3534 (0.0699 0.1978) 0.4278 (0.0710 0.1659) 0.5296 (0.0946 0.1786) 0.2839 (0.0800 0.2816) 0.5548 (0.1180 0.2127) 0.3587 (0.0955 0.2661) 0.3689 (0.0895 0.2426) 0.4046 (0.0798 0.1972) 0.4044 (0.0993 0.2454) 0.3777 (0.0942 0.2493) 0.3342 (0.0781 0.2336) 0.9163 (0.0614 0.0670)
PCM  0.1963 (0.0660 0.3365) 0.2285 (0.0640 0.2802) 0.2268 (0.0555 0.2445) 0.3966 (0.0931 0.2347) 0.2467 (0.0935 0.3790) 0.3636 (0.1106 0.3041) 0.2386 (0.0886 0.3714) 0.2489 (0.0832 0.3342) 0.3340 (0.0933 0.2793) 0.2738 (0.0927 0.3387) 0.2537 (0.0874 0.3447) 0.3184 (0.0721 0.2266)-1.0000 (0.0000 0.0000) 0.9163 (0.0614 0.0670)
MMU  0.3442 (0.0737 0.2141) 0.1245 (0.0245 0.1969) 0.2001 (0.0418 0.2087) 0.2331 (0.0758 0.3250) 0.2105 (0.0672 0.3194) 0.2689 (0.0807 0.3000) 0.1984 (0.0709 0.3572) 0.1786 (0.0594 0.3327) 0.1841 (0.0585 0.3181) 0.2270 (0.0754 0.3321) 0.1810 (0.0699 0.3862) 0.1609 (0.0574 0.3564) 0.1682 (0.0705 0.4192) 0.1830 (0.0763 0.4168) 0.1682 (0.0705 0.4192)
```

```
TREE #  1:  (16, (1, ((((13, 15), 14), 12), ((2, 3), (4, (5, (7, ((9, 6), (8, (10, 11))))))))));   MP score: -1
This is a rooted tree.  Please check!
check convergence..
lnL(ntime: 30  np: 35):   -616.334614      +0.000000
  17..16   17..18   18..1    18..19   19..20   20..21   21..22   22..13   22..15   21..14   20..12   19..23   23..24   24..2    24..3    23..25   25..4    25..26   26..5    26..27   27..7    27..28   28..29   29..9    29..6
28..30   30..8    30..31   31..10   31..11
 0.118722 0.074115 0.249133 0.000004 0.118663 0.146153 0.119262 0.000004 0.000004 0.115870 0.136523 0.177059 0.012937 0.044151 0.000004 0.035304 0.164116 0.039390 0.089812 0.028259 0.067366 0.000004 0.000004 0.064546 0.071478
0.066473 0.000004 0.000004 0.021777 0.022061 3.279198 0.632820 0.267891 0.064803 2.737211

Note: Branch length is defined as number of nucleotide substitutions per codon (not per neucleotide site).

tree length =   1.98320

(16: 0.118722, (1: 0.249133, ((((13: 0.000004, 15: 0.000004): 0.119262, 14: 0.115870): 0.146153, 12: 0.136523): 0.118663, ((2: 0.044151, 3: 0.000004): 0.012937, (4: 0.164116, (5: 0.089812, (7: 0.067366, ((9: 0.064546, 6:
0.071478): 0.000004, (8: 0.000004, (10: 0.021777, 11: 0.022061): 0.000004): 0.066473): 0.000004): 0.028259): 0.039390): 0.035304): 0.177059): 0.000004): 0.074115);

(MMU: 0.118722, (SHI: 0.249133, ((((PSU: 0.000004, PCM: 0.000004): 0.119262, PRO: 0.115870): 0.146153, MAU: 0.136523): 0.118663, ((ASA: 0.044151, ATE: 0.000004): 0.012937, (CGL: 0.164116, (CNI: 0.089812, (MCA: 0.067366, ((MAG:
0.064546, MAR: 0.071478): 0.000004, (MGE: 0.000004, (PDU: 0.021777, PLU: 0.022061): 0.000004): 0.066473): 0.000004): 0.028259): 0.039390): 0.035304): 0.177059): 0.000004): 0.074115);

Detailed output identifying parameters

kappa (ts/tv) =  3.27920


dN/dS (w) for site classes (K=3)

p:   0.63282  0.26789  0.09929
w:   0.06480  1.00000  2.73721
```

dN & dS for each branch

branch	t	N	S	dN/dS	dN	dS	N*dN	S*dS
17..16	0.119	121.0	44.0	0.5807	0.0332	0.0571	4.0	2.5
17..18	0.074	121.0	44.0	0.5807	0.0207	0.0357	2.5	1.6
18..1	0.249	121.0	44.0	0.5807	0.0696	0.1199	8.4	5.3
18..19	0.000	121.0	44.0	0.5807	0.0000	0.0000	0.0	0.0
19..20	0.119	121.0	44.0	0.5807	0.0332	0.0571	4.0	2.5
20..21	0.146	121.0	44.0	0.5807	0.0408	0.0703	4.9	3.1
21..22	0.119	121.0	44.0	0.5807	0.0333	0.0574	4.0	2.5
22..13	0.000	121.0	44.0	0.5807	0.0000	0.0000	0.0	0.0
22..15	0.000	121.0	44.0	0.5807	0.0000	0.0000	0.0	0.0
21..14	0.116	121.0	44.0	0.5807	0.0324	0.0558	3.9	2.5
20..12	0.137	121.0	44.0	0.5807	0.0382	0.0657	4.6	2.9
19..23	0.177	121.0	44.0	0.5807	0.0495	0.0852	6.0	3.8
23..24	0.013	121.0	44.0	0.5807	0.0036	0.0062	0.4	0.3
24..2	0.044	121.0	44.0	0.5807	0.0123	0.0213	1.5	0.9
24..3	0.000	121.0	44.0	0.5807	0.0000	0.0000	0.0	0.0
23..25	0.035	121.0	44.0	0.5807	0.0099	0.0170	1.2	0.7
25..4	0.164	121.0	44.0	0.5807	0.0459	0.0790	5.5	3.5
25..26	0.039	121.0	44.0	0.5807	0.0110	0.0190	1.3	0.8
26..5	0.090	121.0	44.0	0.5807	0.0251	0.0432	3.0	1.9
26..27	0.028	121.0	44.0	0.5807	0.0079	0.0136	1.0	0.6
27..7	0.067	121.0	44.0	0.5807	0.0188	0.0324	2.3	1.4
27..28	0.000	121.0	44.0	0.5807	0.0000	0.0000	0.0	0.0
28..29	0.000	121.0	44.0	0.5807	0.0000	0.0000	0.0	0.0
29..9	0.065	121.0	44.0	0.5807	0.0180	0.0311	2.2	1.4
29..6	0.071	121.0	44.0	0.5807	0.0200	0.0344	2.4	1.5
28..30	0.066	121.0	44.0	0.5807	0.0186	0.0320	2.2	1.4
30..8	0.000	121.0	44.0	0.5807	0.0000	0.0000	0.0	0.0
30..31	0.000	121.0	44.0	0.5807	0.0000	0.0000	0.0	0.0
31..10	0.022	121.0	44.0	0.5807	0.0061	0.0105	0.7	0.5
31..11	0.022	121.0	44.0	0.5807	0.0062	0.0106	0.7	0.5

Naive Empirical Bayes (NEB) analysis
Positively selected sites (*: P>95%; **: P>99%)
(amino acids refer to 1st sequence: SHI)

	Pr(w>1)	post mean +- SE for w
6 T	0.903	2.568
12 H	0.749	2.302
24 *	0.783	2.359

Bayes Empirical Bayes (BEB) analysis (Yang, Wong & Nielsen 2005. Mol. Biol. Evol. 22:1107-1118)
Positively selected sites (*: P>95%; **: P>99%)
(amino acids refer to 1st sequence: SHI)

	Pr(w>1)	post mean +- SE for w
6 T	0.864	3.052 +- 1.837
12 H	0.646	2.215 +- 1.398
24 *	0.727	2.580 +- 1.684

The grid (see ternary graph for p0-p1)

w0:	0.050	0.150	0.250	0.350	0.450	0.550	0.650	0.750	0.850	0.950
w2:	1.500	2.500	3.500	4.500	5.500	6.500	7.500	8.500	9.500	10.500

Posterior on the grid

w0:	0.725	0.252	0.022	0.001	0.000	0.000	0.000	0.000	0.000	0.000
w2:	0.240	0.288	0.189	0.118	0.070	0.041	0.024	0.015	0.009	0.006

Posterior for p0-p1 (see the ternary graph)

0.000																		
0.000	0.000	0.000																
0.000	0.000	0.000	0.000	0.000														
0.000	0.000	0.000	0.000	0.000	0.000	0.001												
0.000	0.000	0.000	0.000	0.000	0.000	0.000	0.002	0.014										
0.000	0.000	0.000	0.000	0.000	0.000	0.000	0.001	0.008	0.028	0.068								
0.000	0.000	0.000	0.000	0.000	0.000	0.000	0.000	0.003	0.012	0.045	0.094	0.099						
0.000	0.000	0.000	0.000	0.000	0.000	0.000	0.000	0.001	0.005	0.019	0.044	0.072	0.097	0.044				
0.000	0.000	0.000	0.000	0.000	0.000	0.000	0.000	0.001	0.003	0.010	0.021	0.035	0.052	0.039	0.033	0.007		
0.000	0.000	0.000	0.000	0.000	0.000	0.000	0.000	0.001	0.002	0.007	0.014	0.024	0.030	0.025	0.023	0.009	0.005	0.000

sum of density on p0-p1 =   1.000000

Time used: 1:53
