# Supplementary material for: Sexual Selection Halts the Relaxation of Protamine 2 among Rodents
Source: PLoS One. 2011 Dec 21;6(12):e29247. doi: 10.1371/journal.pone.0029247 (PMC3244444; doi:10.1371/journal.pone.0029247)
Supplement: Figure S8 — Codeml output file (M2) for mature-Protamine 2. Included species of Cricetidae represented by abbreviated code: Arvicola sadipus (ASA), Arvicola terrestris (ATE), Clethrionomys glareolus (CGL), Cricetelus griseus (CGR), Chionomys nivalis (CNI), Microtus agrestis (MAG), Microtus arvalis (MAR), Mesocricetus auratus (MAU), Microtus cabrerae (MCA), Microtus gerbei (MGE), Mus musculus musculus (MMU), Phodopus campbelli (PCM), Pitymys duodecimcostatus (PDU), Pitymys lusitanicus (PLU), Phodopus roborovskii (PRO), Phodopus sungorus (PSU), Sigmodon hispidus (SHI). (PDF) [file pone.0029247.s008.pdf]

Figure S8. Mature Protamine 2
Codeml output file M2

Arvicola sadipus (ASA)
Arvicola terrestris (ATE)
Clethrionomys glareolus (CGL)
Cricetelus griseus (CGR)
Chionomys nivalis (CNI)
Microtus agrestis (MAG)
Microtus arvalis (MAR)
Mesocricetus auratus (MAU)
Microtus cabrerae (MCA)
Microtus gerbei (MGE)
Mus musculus musculus (MMU)
Phodopus campbelli (PCM)
Pitymys duodecimcostatus (PDU)
Pitymys lusitanicus (PLU)
Phodopus roborovskii (PRO)
Phodopus sugorus (PSU)
Sigmodon hispidus (SHI)

```
     16    162

SHI  TCG CGT AGG AGA CTG CAC CGG ATC CAT AAG AGA CGT CGG TCA TGC AGG AGG CGA AGG AGA CAC TCC TGC CGC CAC AGG AGG CGG CAT CGC AGA GGC TGC AAA AGA TCC CG- --- --G AGG AGG AGA TGC AGG TGC AGG AAG TGT AGG AGG CAC CAC CAC ---
ASA  TCG CGT AGA AAG TTG TAC CGG ATC CAT AGG AGA CGC AGG TCA TGC AGG AGG CG- --G AGA CGT TCC TGC CGC CAC AGG AGG CGG CAT CGT AGG GGC TGC AGA AGA TCC CG- --A AGG AGG AGG AGA TGT AGG TGC AGG AAG TGT AGG AGG CAC CGC CAC ---
ATE  TCG CGT AGA AAG TTG TAC CGG ATC CAC AGG AGA CGC AGG TCA TGC AGG AGG AG- --G AGA CGT TCC TKC CGC CAC AGG AGG CGG CAT CGT AGG GGC TGC AGA AGA TCC CG- --A AGG AGG AGG AGA TGT AGG TGC AGG AAG TGT AGG AGG CAC CGC CAC ---
CGL  TCG CGT AGG AAG TTG TAC CGG ATC CAC AGG AGA CGC CGG TCA TGC AGG AGG CG- --G AGA CGT TCC TGC CGC CAC AGG AGG CGG CAT CGT AGA GGC GGC AGA AGA TGC CG- --A AGG AGG AGG AGA TGT AGG TGC AGA AAG TGT AGG AGG TAC CGC TAC ---
CNI  TCG CGT AGG AAG TTG CAT CGG ATC CAC AGG AGA CGC CGG TCA TGC AGG AGG CG- --G AGA CGT TCC TGC CGC CAC AGG AGG CGG CAT CGT AGA GGC TGC AGA AGA TCC CG- --G AGG AGG AGG AGA TGT AGG TGC AGG AAG TGT AGG CGC CAC CAC --- ---
MAR  TCG CGT AGG AAG TTG CAT CGG ATC CAC AGG AGA CGC CGG TCA TGC AGG AGG CG- --G AGA CGT TCC TGC CGC CAC AGG AGG CGG CAT CGT AGA GGC TGC AGA AGA TCC CGA AGG AGG AGG AGG AGA TGT AGG TGC AGG AAG TGT AGG AGG CGC TAC CAC ---
MCA  TCG CGT AGG AAG TTG CAT CGG ATC CAC AGG AGA CGC CGG TCA TGC AGG AGG CG- --G AGA CGT TCC TGC CGC CAC AGG AGG CGG CAT CGT AGA GGC TGC AGA AGA TCC CG- --A AGG AGG AGG AGA TGT AGG TGC AGG AGG TGT AGG AGG CGC CAC CAC ---
MGE  TCG CGT AGG AAG TTG CAT CGG ATC CAC AGG AGA CGC CGG TCA TGC AGG AGG CG- --G AGA CGT TCC TGC CGC CAC AGG AGG CGG CAT CGT AGA GGC TGC AGA AGA TCC CGA AGG AGG AGG AGG AGA TGT AGG TGC AGG AGG TGT AGG AGG CGC TAC CAC ---
MAG  TCG CGT AGG AAG TTG CAT CGG ATC CAC AGG AGA CGC CGG TCA TGC AGG AGG CG- --G AGA CGT TCC TGC CGC CAC AGG AGG CGG CAT CGT AGA GGC TGC AGA AGA TCC CGA AGG AGG AGG A-- -GA TGT AGG TGC AGG AGG TGT AGG AGG CGC TAC CAC ---
PDU  TCG CGT AGG AAG TTG CAT CGG ATC CAC AGG AGA CGC CGG TCA TGC AGG AGG CG- --G AGA CGT TCC TGC CGC CAC AGG AGG CGG CAT CGT AGA GGC TGC AGA AGA TCC CGA AGG AGG AGG AGG AGA TGT AGG TGC AGG AGG TGT AGG AGG CGC TAC CAC ---
PLU  TCG CGT AGG AAG TTG CAT CGG ATC CAC AGG AGA CGC CGG TCA TGC AGG AGG CG- --G AGA CGT TCC TGC CGC CAC AGG AGG CGG CAT CGT AGA GGC TGC AGA AGA TCC CGA AGG AGG AGG AGG AGA TGT AGG TGC AGG AGG TGT AGG AGG CGC TAC CAC ---
MAU  TCG CGT AGG AAG CTG TAC CGA ATC CAC AGG AGG CGC CGG TCA TGC AGG AGG CGG AGG AGA CAC TCC TGC CGT CAC AGG AGA CGG CAT CGC AGA GGC TGC AGA AGA TCA CG- --C AGG AGG AGG AGA TGC AGG TGC AGG AGG TGT AGG AGG CAC CAC CAC ---
PSU  TCG CGT AGA AGG CTG CAC CGG ATC CAC AAG AGG CGC CGG TCA TGC AGG AGG CG- --G AGA CAC TCC TGC TGC CAC AGG AGG CGG CAT CGC AGA GGC TGC AGA AGA TCC CG- --C AGG AGG AGG AGA TGC AGG TGC AGG AAG TGT AGG AGG CAC CAC CAC CAC
PRO  TCA CGT AGG AGG CTG CAC CGG ATC CAC AAG AGG CGC CGG TCA TGC AGG AGG CG- --G AGA CAC TCC TGC CGC CAC AGG AGG CGG CAT CGC AGA GGC TGC AGA AGA TCC CG- --C AGG AGG AGG AGA TGC AGG TGC AGG AAG TGT AGG AGG CAC CAC TAC CAC
PCM  TCG CGT AGA AGG CTG CAC CGG ATC CAC AAG AGG CGC CGG TCA TGC AGG AGG CG- --G AGA CAC TCC TGC TGC CAC AGG AGG CGG CAT CGC AGA GGC TGC AGA AGA TCC CG- --C AGG AGG AGG AGA TGC AGG TGC AGG AAG TGT AGG AGG CAC CAC CAC ---
MMU  TCT CGT AAG AGG CTA CAT AGG ATC CAC AAG AGG CGT CGG TCA TGC AGA AGG CGG AGG AGA CAC TCC TGC CGC CAC AGG AGG CGG CAT CGC AGA GGC TGC AGA AGA TCC CG- --A AGG AGG AGG AGA TGC AGG TGC AGG AAA TGT AGG AGG CAC CAT CAC ---
```

Printing out site pattern counts

```
        16        144  P

SHI  --- --- --G AAA AAG AAG AGA AGA AGA AGA AGA AGG AGG AGG AGG AGG AGG AGG AGG ATC CAC CAC CAC CAC CAC CAC CAT CAT CG- CGA CGC CGC CGG CGG CGG CGT CGT CTG GGC TCA TCC TCC TCG TGC TGC TGC TGC TGT
ASA  --- --A AGG AGA AAG AGG AAG AGA AGA AGA AGG --G AGA AGG AGG AGG AGG AGG AGG ATC CAC CAC CAC CGC CGT TAC CAT CAT CG- CG- CGC CGT AGG CGG CGG CGC CGT TTG GGC TCA TCC TCC TCG TGC TGC TGC TGT TGT
ATE  --- --A AGG AGA AAG AGG AAG AGA AGA AGA AGG --G AGA AGG AGG AGG AGG AGG AGG ATC CAC CAC CAC CGC CGT TAC CAC CAT CG- AG- CGC CGT AGG CGG CGG CGC CGT TTG GGC TCA TCC TCC TCG TGC TGC TKC TGT TGT
CGL  --- --A AGG AGA AAG AGG AAG AGA AGA AGA AGA --G AGG AGA AGG AGG AGG AGG AGG ATC CAC TAC TAC CGC CGT TAC CAC CAT CG- CG- CGC CGT CGG CGG CGG CGC CGT TTG GGC TCA TCC TGC TCG GGC TGC TGC TGT TGT
CNI  --- --G AGG AGA AAG AGG AAG AGA AGA AGA AGA --G AGG AGG AGG AGG AGG AGG CGC ATC CAC --- CAC CAC CGT CAT CAC CAT CG- CG- CGC CGT CGG CGG CGG CGC CGT TTG GGC TCA TCC TCC TCG TGC TGC TGC TGT TGT
```

```
MAR  --- AGG AGG AGA AAG AGG AAG AGA AGA AGA AGA --G AGG AGG AGG AGG AGG AGG AGG ATC CAC CAC CGC TAC CGT CAT CAC CAT CGA CG- CGC CGT CGG CGG CGG CGC CGT TTG GGC TCA TCC TCC TCG TGC TGC TGC TGT TGT
MCA  --- --A AGG AGA AGG AGG AAG AGA AGA AGA AGA --G AGG AGG AGG AGG AGG AGG AGG ATC CAC CAC CGC CAC CGT CAT CAC CAT CG- CG- CGC CGT CGG CGG CGG CGC CGT TTG GGC TCA TCC TCC TCG TGC TGC TGC TGT TGT
MGE  --- AGG AGG AGA AGG AGG AAG AGA AGA AGA AGA --G AGG AGG AGG AGG AGG AGG AGG ATC CAC CAC CGC TAC CGT CAT CAC CAT CGA CG- CGC CGT CGG CGG CGG CGC CGT TTG GGC TCA TCC TCC TCG TGC TGC TGC TGT TGT
MAG  --- AGG AGG AGA AGG AGG AAG -GA AGA AGA AGA --G AGG AGG A-- AGG AGG AGG AGG ATC CAC CAC CGC TAC CGT CAT CAC CAT CGA CG- CGC CGT CGG CGG CGG CGC CGT TTG GGC TCA TCC TCC TCG TGC TGC TGC TGT TGT
PDU  --- AGG AGG AGA AGG AGG AAG AGA AGA AGA AGA --G AGG AGG AGG AGG AGG AGG AGG ATC CAC CAC CGC TAC CGT CAT CAC CAT CGA CG- CGC CGT CGG CGG CGG CGC CGT TTG GGC TCA TCC TCC TCG TGC TGC TGC TGT TGT
PLU  --- AGG AGG AGA AGG AGG AAG AGA AGA AGA AGA --G AGG AGG AGG AGG AGG AGG AGG ATC CAC CAC CGC TAC CGT CAT CAC CAT CGA CG- CGC CGT CGG CGG CGG CGC CGT TTG GGC TCA TCC TCC TCG TGC TGC TGC TGT TGT
MAU  --- --C AGG AGA AGG AGG AAG AGA AGA AGG AGA AGG AGG AGG AGG AGA AGG AGG AGG ATC CAC CAC CAC CAC CAC CAC CAC TAC CAC CAT CG- CGG CGT CGC CGG CGA CGG CGC CGT CTG GGC TCA TCC TCA TCG TGC TGC TGC TGC TGT
PSU  CAC --C AGG AGA AAG AAG AGG AGA AGA AGG AGA --G AGA AGG AGG AGG AGG AGG AGG ATC CAC CAC CAC CAC CAC CAC CAC CAC CAC CAT CG- CG- TGC CGC CGG CGG CGG CGC CGT CTG GGC TCA TCC TCC TCG TGC TGC TGC TGC TGT
PRO  CAC --C AGG AGA AAG AAG AGG AGA AGA AGG AGA --G AGG AGG AGG AGG AGG AGG AGG ATC CAC TAC CAC CAC CAC CAC CAC CAC CAC CAT CG- CG- CGC CGC CGG CGG CGG CGC CGT CTG GGC TCA TCC TCC TCA TGC TGC TGC TGC TGT
PCM  --- --C AGG AGA AAG AAG AGG AGA AGA AGG AGA --G AGA AGG AGG AGG AGG AGG AGG ATC CAC CAC CAC CAC CAC CAC CAC CAC CAC CAT CG- CG- TGC CGC CGG CGG CGG CGC CGT CTG GGC TCA TCC TCC TCG TGC TGC TGC TGC TGT
MMU  --- --A AGG AGA AAA AAG AGG AGA AGA AGG AGA AGG AAG AGG AGG AGG AGA AGG AGG ATC CAC CAC CAC CAT CAC CAT CAC CAT CAC CAT CG- CGG CGC CGC CGG AGG CGG CGT CGT CTA GGC TCA TCC TCC TCT TGC TGC TGC TGC TGT



    1    1    1    1    1    1    1    1    2    1    1    1    1    1    1
    1    1    5    1    1    1    1    1    1    1    1    1    1    1    1
    1    1    1    1    1    1    1    1    1    1    1    1    1    1    2
    1    1    1

CODONML (in paml version 4.4d, March 2011)  1.phy
Model: One dN/dS ratio for branches
Codon frequency model: Fcodon
Site-class models:  PositiveSelection
ns =  16  ls =  54

Codon usage in sequences
--------------------------------------------------------------------------------------------------------------
Phe TTT  0  0  0  0  0  0 | Ser TCT  0  0  0  0  0  0 | Tyr TAT  0  0  0  0  0  0 | Cys TGT  1  2  2  2  2  2
    TTC  0  0  0  0  0  0 |     TCC  2  2  2  1  2  2 |     TAC  0  1  1  3  0  1 |     TGC  5  4  3  4  4  4
Leu TTA  0  0  0  0  0  0 |     TCA  1  1  1  1  1  1 | *** TAA  0  0  0  0  0  0 | *** TGA  0  0  0  0  0  0
    TTG  0  1  1  1  1  1 |     TCG  1  1  1  1  1  1 |     TAG  0  0  0  0  0  0 | Trp TGG  0  0  0  0  0  0
--------------------------------------------------------------------------------------------------------------
Leu CTT  0  0  0  0  0  0 | Pro CCT  0  0  0  0  0  0 | His CAT  2  2  1  1  2  2 | Arg CGT  2  3  3  3  3  3
    CTC  0  0  0  0  0  0 |     CCC  0  0  0  0  0  0 |     CAC  6  3  4  2  4  3 |     CGC  2  3  3  3  3  3
    CTA  0  0  0  0  0  0 |     CCA  0  0  0  0  0  0 | Gln CAA  0  0  0  0  0  0 |     CGA  1  0  0  0  0  1
    CTG  1  0  0  0  0  0 |     CCG  0  0  0  0  0  0 |     CAG  0  0  0  0  0  0 |     CGG  3  2  2  3  3  3
--------------------------------------------------------------------------------------------------------------
Ile ATT  0  0  0  0  0  0 | Thr ACT  0  0  0  0  0  0 | Asn AAT  0  0  0  0  0  0 | Ser AGT  0  0  0  0  0  0
    ATC  1  1  1  1  1  1 |     ACC  0  0  0  0  0  0 |     AAC  0  0  0  0  0  0 |     AGC  0  0  0  0  0  0
    ATA  0  0  0  0  0  0 |     ACA  0  0  0  0  0  0 | Lys AAA  1  0  0  0  0  0 | Arg AGA  6  6  6  7  6  6
Met ATG  0  0  0  0  0  0 |     ACG  0  0  0  0  0  0 |     AAG  2  2  2  2  2  2 |     AGG 12 14 14 12 12 14
--------------------------------------------------------------------------------------------------------------
Val GTT  0  0  0  0  0  0 | Ala GCT  0  0  0  0  0  0 | Asp GAT  0  0  0  0  0  0 | Gly GGT  0  0  0  0  0  0
    GTC  0  0  0  0  0  0 |     GCC  0  0  0  0  0  0 |     GAC  0  0  0  0  0  0 |     GGC  1  1  1  2  1  1
    GTA  0  0  0  0  0  0 |     GCA  0  0  0  0  0  0 | Glu GAA  0  0  0  0  0  0 |     GGA  0  0  0  0  0  0
    GTG  0  0  0  0  0  0 |     GCG  0  0  0  0  0  0 |     GAG  0  0  0  0  0  0 |     GGG  0  0  0  0  0  0
--------------------------------------------------------------------------------------------------------------


--------------------------------------------------------------------------------------------------------------
Phe TTT  0  0  0  0  0  0 | Ser TCT  0  0  0  0  0  0 | Tyr TAT  0  0  0  0  0  0 | Cys TGT  2  2  2  2  2  1
    TTC  0  0  0  0  0  0 |     TCC  2  2  2  2  2  1 |     TAC  0  1  1  1  1  1 |     TGC  4  4  4  4  4  5
Leu TTA  0  0  0  0  0  0 |     TCA  1  1  1  1  1  2 | *** TAA  0  0  0  0  0  0 | *** TGA  0  0  0  0  0  0
    TTG  1  1  1  1  1  0 |     TCG  1  1  1  1  1  1 |     TAG  0  0  0  0  0  0 | Trp TGG  0  0  0  0  0  0
--------------------------------------------------------------------------------------------------------------
Leu CTT  0  0  0  0  0  0 | Pro CCT  0  0  0  0  0  0 | His CAT  2  2  2  2  2  1 | Arg CGT  3  3  3  3  3  2
    CTC  0  0  0  0  0  0 |     CCC  0  0  0  0  0  0 |     CAC  4  3  3  3  3  6 |     CGC  3  3  3  3  3  2
    CTA  0  0  0  0  0  0 |     CCA  0  0  0  0  0  0 | Gln CAA  0  0  0  0  0  0 |     CGA  0  1  1  1  1  1
    CTG  0  0  0  0  0  1 |     CCG  0  0  0  0  0  0 |     CAG  0  0  0  0  0  0 |     CGG  3  3  3  3  3  3
```

```
------------------------------------------------------------------------------------------------------------
Ile ATT  0  0  0  0  0  0 | Thr ACT  0  0  0  0  0  0 | Asn AAT  0  0  0  0  0  0 | Ser AGT  0  0  0  0  0  0
    ATC  1  1  1  1  1  1 |     ACC  0  0  0  0  0  0 |     AAC  0  0  0  0  0  0 |     AGC  0  0  0  0  0  0
    ATA  0  0  0  0  0  0 |     ACA  0  0  0  0  0  0 | Lys AAA  0  0  0  0  0  0 | Arg AGA  6  6  5  6  6  6
Met ATG  0  0  0  0  0  0 |     ACG  0  0  0  0  0  0 |     AAG  1  1  1  1  1  1 |     AGG 14 15 14 15 15 15
------------------------------------------------------------------------------------------------------------
Val GTT  0  0  0  0  0  0 | Ala GCT  0  0  0  0  0  0 | Asp GAT  0  0  0  0  0  0 | Gly GGT  0  0  0  0  0  0
    GTC  0  0  0  0  0  0 |     GCC  0  0  0  0  0  0 |     GAC  0  0  0  0  0  0 |     GGC  1  1  1  1  1  1
    GTA  0  0  0  0  0  0 |     GCA  0  0  0  0  0  0 | Glu GAA  0  0  0  0  0  0 |     GGA  0  0  0  0  0  0
    GTG  0  0  0  0  0  0 |     GCG  0  0  0  0  0  0 |     GAG  0  0  0  0  0  0 |     GGG  0  0  0  0  0  0
------------------------------------------------------------------------------------------------------------


------------------------------------------------------------------------------------
Phe TTT  0  0  0  0 | Ser TCT  0  0  0  1 | Tyr TAT  0  0  0  0 | Cys TGT  1  1  1  1
    TTC  0  0  0  0 |     TCC  2  2  2  2 |     TAC  0  1  0  0 |     TGC  6  5  6  5
Leu TTA  0  0  0  0 |     TCA  1  2  1  1 | *** TAA  0  0  0  0 | *** TGA  0  0  0  0
    TTG  0  0  0  0 |     TCG  1  0  1  0 |     TAG  0  0  0  0 | Trp TGG  0  0  0  0
------------------------------------------------------------------------------------
Leu CTT  0  0  0  0 | Pro CCT  0  0  0  0 | His CAT  1  1  1  3 | Arg CGT  1  1  1  2
    CTC  0  0  0  0 |     CCC  0  0  0  0 |     CAC  8  7  7  5 |     CGC  2  3  2  2
    CTA  0  0  0  1 |     CCA  0  0  0  0 | Gln CAA  0  0  0  0 |     CGA  0  0  0  0
    CTG  1  1  1  0 |     CCG  0  0  0  0 |     CAG  0  0  0  0 |     CGG  3  3  3  3
------------------------------------------------------------------------------------
Ile ATT  0  0  0  0 | Thr ACT  0  0  0  0 | Asn AAT  0  0  0  0 | Ser AGT  0  0  0  0
    ATC  1  1  1  1 |     ACC  0  0  0  0 |     AAC  0  0  0  0 |     AGC  0  0  0  0
    ATA  0  0  0  0 |     ACA  0  0  0  0 | Lys AAA  0  0  0  1 | Arg AGA  6  5  6  6
Met ATG  0  0  0  0 |     ACG  0  0  0  0 |     AAG  2  2  2  2 |     AGG 13 14 13 14
------------------------------------------------------------------------------------
Val GTT  0  0  0  0 | Ala GCT  0  0  0  0 | Asp GAT  0  0  0  0 | Gly GGT  0  0  0  0
    GTC  0  0  0  0 |     GCC  0  0  0  0 |     GAC  0  0  0  0 |     GGC  1  1  1  1
    GTA  0  0  0  0 |     GCA  0  0  0  0 | Glu GAA  0  0  0  0 |     GGA  0  0  0  0
    GTG  0  0  0  0 |     GCG  0  0  0  0 |     GAG  0  0  0  0 |     GGG  0  0  0  0
------------------------------------------------------------------------------------

Codon position x base (3x4) table for each sequence.

#1: SHI
position  1:    T:0.20000    C:0.34000    A:0.44000    G:0.02000
position  2:    T:0.04000    C:0.08000    A:0.22000    G:0.66000
position  3:    T:0.10000    C:0.34000    A:0.18000    G:0.38000
Average         T:0.11333    C:0.25333    A:0.28000    G:0.35333

#2: ASA
position  1:    T:0.24490    C:0.26531    A:0.46939    G:0.02041
position  2:    T:0.04082    C:0.08163    A:0.16327    G:0.71429
position  3:    T:0.14286    C:0.30612    A:0.14286    G:0.40816
Average         T:0.14286    C:0.21769    A:0.25850    G:0.38095

#3: ATE
position  1:    T:0.22917    C:0.27083    A:0.47917    G:0.02083
position  2:    T:0.04167    C:0.08333    A:0.16667    G:0.70833
position  3:    T:0.12500    C:0.31250    A:0.14583    G:0.41667
Average         T:0.13194    C:0.22222    A:0.26389    G:0.38194

#4: CGL
position  1:    T:0.26531    C:0.24490    A:0.44898    G:0.04082
position  2:    T:0.04082    C:0.06122    A:0.16327    G:0.73469
position  3:    T:0.12245    C:0.32653    A:0.16327    G:0.38776
```

```
Average         T:0.14286    C:0.21088    A:0.25850    G:0.38776

#5: CNI
position  1:    T:0.22917    C:0.31250    A:0.43750    G:0.02083
position  2:    T:0.04167    C:0.08333    A:0.16667    G:0.70833
position  3:    T:0.14583    C:0.31250    A:0.14583    G:0.39583
Average         T:0.13889    C:0.23611    A:0.25000    G:0.37500

#6: MAR
position  1:    T:0.23529    C:0.29412    A:0.45098    G:0.01961
position  2:    T:0.03922    C:0.07843    A:0.15686    G:0.72549
position  3:    T:0.13725    C:0.29412    A:0.15686    G:0.41176
Average         T:0.13725    C:0.22222    A:0.25490    G:0.38562

#7: MCA
position  1:    T:0.22449    C:0.30612    A:0.44898    G:0.02041
position  2:    T:0.04082    C:0.08163    A:0.14286    G:0.73469
position  3:    T:0.14286    C:0.30612    A:0.14286    G:0.40816
Average         T:0.13605    C:0.23129    A:0.24490    G:0.38776

#8: MGE
position  1:    T:0.23529    C:0.29412    A:0.45098    G:0.01961
position  2:    T:0.03922    C:0.07843    A:0.13725    G:0.74510
position  3:    T:0.13725    C:0.29412    A:0.15686    G:0.41176
Average         T:0.13725    C:0.22222    A:0.24837    G:0.39216

#9: MAG
position  1:    T:0.24490    C:0.30612    A:0.42857    G:0.02041
position  2:    T:0.04082    C:0.08163    A:0.14286    G:0.73469
position  3:    T:0.14286    C:0.30612    A:0.14286    G:0.40816
Average         T:0.14286    C:0.23129    A:0.23810    G:0.38776

#10: PDU
position  1:    T:0.23529    C:0.29412    A:0.45098    G:0.01961
position  2:    T:0.03922    C:0.07843    A:0.13725    G:0.74510
position  3:    T:0.13725    C:0.29412    A:0.15686    G:0.41176
Average         T:0.13725    C:0.22222    A:0.24837    G:0.39216

#11: PLU
position  1:    T:0.23529    C:0.29412    A:0.45098    G:0.01961
position  2:    T:0.03922    C:0.07843    A:0.13725    G:0.74510
position  3:    T:0.13725    C:0.29412    A:0.15686    G:0.41176
Average         T:0.13725    C:0.22222    A:0.24837    G:0.39216

#12: MAU
position  1:    T:0.21569    C:0.31373    A:0.45098    G:0.01961
position  2:    T:0.03922    C:0.07843    A:0.17647    G:0.70588
position  3:    T:0.07843    C:0.33333    A:0.17647    G:0.41176
Average         T:0.11111    C:0.24183    A:0.26797    G:0.37908

#13: PSU
position  1:    T:0.22000    C:0.32000    A:0.44000    G:0.02000
position  2:    T:0.04000    C:0.08000    A:0.22000    G:0.66000
position  3:    T:0.06000    C:0.40000    A:0.14000    G:0.40000
Average         T:0.10667    C:0.26667    A:0.26667    G:0.36000

#14: PRO
position  1:    T:0.22000    C:0.32000    A:0.44000    G:0.02000
```

position 2: T:0.04000 C:0.08000 A:0.22000 G:0.66000
position 3: T:0.06000 C:0.40000 A:0.14000 G:0.40000
Average T:0.10667 C:0.26667 A:0.26667 G:0.36000

#15: PCM
position 1: T:0.22449 C:0.30612 A:0.44898 G:0.02041
position 2: T:0.04082 C:0.08163 A:0.20408 G:0.67347
position 3: T:0.06122 C:0.38776 A:0.14286 G:0.40816
Average T:0.10884 C:0.25850 A:0.26531 G:0.36735

#16: MMU
position 1: T:0.19608 C:0.31373 A:0.47059 G:0.01961
position 2: T:0.03922 C:0.07843 A:0.21569 G:0.66667
position 3: T:0.13725 C:0.31373 A:0.17647 G:0.37255
Average T:0.12418 C:0.23529 A:0.28758 G:0.35294

Sums of codon usage counts

Phe F	TTT	0	Ser S	TCT	1	Tyr Y	TAT	0	Cys C	TGT	26
	TTC	0		TCC	30		TAC	12		TGC	71
Leu L	TTA	0		TCA	18	*** *	TAA	0	*** *	TGA	0
	TTG	10		TCG	14		TAG	0	Trp W	TGG	0
Leu L	CTT	0	Pro P	CCT	0	His H	CAT	27	Arg R	CGT	39
	CTC	0		CCC	0		CAC	71		CGC	43
	CTA	1		CCA	0	Gln Q	CAA	0		CGA	7
	CTG	5		CCG	0		CAG	0		CGG	46
Ile I	ATT	0	Thr T	ACT	0	Asn N	AAT	0	Ser S	AGT	0
	ATC	16		ACC	0		AAC	0		AGC	0
	ATA	0		ACA	0	Lys K	AAA	2	Arg R	AGA	95
Met M	ATG	0		ACG	0		AAG	26		AGG	220
Val V	GTT	0	Ala A	GCT	0	Asp D	GAT	0	Gly G	GGT	0
	GTC	0		GCC	0		GAC	0		GGC	17
	GTA	0		GCA	0	Glu E	GAA	0		GGA	0
	GTG	0		GCG	0		GAG	0		GGG	0

(Ambiguity data are not used in the counts.)

Codon position x base (3x4) table, overall

position 1: T:0.22836 C:0.29987 A:0.45044 G:0.02133
position 2: T:0.04015 C:0.07905 A:0.17315 G:0.70765
position 3: T:0.11669 C:0.32622 A:0.15433 G:0.40276
Average T:0.12840 C:0.23505 A:0.25931 G:0.37725

Codon frequencies under model, for use in evolver (TTT TTC TTA TTG ... GGG):

```
  0.00000000  0.00000000  0.00000000  0.01232779
  0.00117925  0.03590478  0.02207005  0.01725890
  0.00000000  0.01436191  0.00000000  0.00000000
  0.03066038  0.08617148  0.00000000  0.00000000
  0.00000000  0.00000000  0.00122611  0.00616389
  0.00000000  0.00000000  0.00000000  0.00000000
  0.03183962  0.08497465  0.00000000  0.00000000
  0.05362182  0.06015171  0.01018747  0.06661921
```

```
  0.00000000  0.01920087  0.00000000  0.00000000
  0.00000000  0.00000000  0.00000000  0.00000000
  0.00000000  0.00000000  0.00245900  0.03214130
  0.00000000  0.00000000  0.11830660  0.27282714
  0.00000000  0.00000000  0.00000000  0.00000000
  0.00000000  0.00000000  0.00000000  0.00000000
  0.00000000  0.00000000  0.00000000  0.00000000
  0.00000000  0.02034604  0.00000000  0.00000000
```

```
Nei & Gojobori 1986. dN/dS (dN, dS)
(Pairwise deletion)
(Note: This matrix is not used in later ML. analysis.
Use runmode = -2 for ML pairwise comparison.)

SHI
ASA  0.1706 (0.0564 0.3308)
ATE  0.1505 (0.0579 0.3844) 0.0000 (0.0000 0.0299)
CGL  0.3464 (0.0971 0.2803) 0.2321 (0.0366 0.1579) 0.3001 (0.0375 0.1250)
CNI  0.1438 (0.0480 0.3336) 0.1401 (0.0279 0.1989) 0.1742 (0.0286 0.1640) 0.6236 (0.0571 0.0915)
MAR  0.2005 (0.0566 0.2824) 0.2301 (0.0366 0.1589) 0.2976 (0.0375 0.1259) 1.2866 (0.0754 0.0586) 0.9512 (0.0280 0.0294)
MCA  0.2010 (0.0567 0.2819) 0.2306 (0.0366 0.1586) 0.2982 (0.0375 0.1257) 1.2890 (0.0754 0.0585) 0.9530 (0.0280 0.0294)-1.0000 (0.0181 0.0000)
MGE  0.2369 (0.0666 0.2811) 0.2910 (0.0460 0.1582) 0.3764 (0.0472 0.1254) 1.4644 (0.0855 0.0584) 1.2829 (0.0376 0.0293)-1.0000 (0.0087 0.0000)-1.0000 (0.0090 0.0000)
MAG  0.2353 (0.0696 0.2959) 0.2902 (0.0481 0.1657) 0.3759 (0.0493 0.1312) 1.4681 (0.0894 0.0609) 1.2853 (0.0393 0.0305)-1.0000 (0.0091 0.0000)-1.0000 (0.0094 0.0000)-1.0000 (0.0000 0.0000)
PDU  0.2369 (0.0666 0.2811) 0.2910 (0.0460 0.1582) 0.3764 (0.0472 0.1254) 1.4644 (0.0855 0.0584) 1.2829 (0.0376 0.0293)-1.0000 (0.0087 0.0000)-1.0000 (0.0090 0.0000)-1.0000 (0.0000 0.0000)-1.0000 (0.0000 0.0000)
PLU  0.2369 (0.0666 0.2811) 0.2910 (0.0460 0.1582) 0.3764 (0.0472 0.1254) 1.4644 (0.0855 0.0584) 1.2829 (0.0376 0.0293)-1.0000 (0.0087 0.0000)-1.0000 (0.0090 0.0000)-1.0000 (0.0000 0.0000)-1.0000 (0.0000 0.0000)-1.0000 (0.0000 0.0000)
MAU  0.1482 (0.0452 0.3048) 0.0526 (0.0272 0.5173) 0.0594 (0.0279 0.4696) 0.1843 (0.0655 0.3552) 0.0905 (0.0375 0.4142) 0.1287 (0.0461 0.3579) 0.0765 (0.0273 0.3573) 0.1030 (0.0367 0.3562) 0.1020 (0.0383 0.3755) 0.1030 (0.0367 0.3562) 0.1030 (0.0367 0.3562)
PSU  0.1088 (0.0183 0.1680) 0.1962 (0.0551 0.2810) 0.2326 (0.0565 0.2429) 0.4033 (0.0948 0.2350) 0.1654 (0.0469 0.2832) 0.2337 (0.0553 0.2367) 0.2342 (0.0553 0.2363) 0.2760 (0.0650 0.2356) 0.2745 (0.0679 0.2475) 0.2760 (0.0650 0.2356) 0.2760 (0.0650 0.2356) 0.2828 (0.0458 0.1618)
PRO  0.1106 (0.0183 0.1658) 0.1512 (0.0553 0.3660) 0.1745 (0.0567 0.3250) 0.3238 (0.0751 0.2320) 0.1333 (0.0374 0.2802) 0.2377 (0.0555 0.2336) 0.2382 (0.0555 0.2332) 0.2806 (0.0653 0.2326) 0.2793 (0.0682 0.2441) 0.2806 (0.0653 0.2326) 0.2806 (0.0653 0.2326) 0.2874 (0.0459 0.1598) 0.2894 (0.0175 0.0605)
PCM  0.1088 (0.0183 0.1680) 0.1962 (0.0551 0.2810) 0.2326 (0.0565 0.2429) 0.4033 (0.0948 0.2350) 0.1654 (0.0469 0.2832) 0.2337 (0.0553 0.2367) 0.2342 (0.0553 0.2363) 0.2760 (0.0650 0.2356) 0.2745 (0.0679 0.2475) 0.2760 (0.0650 0.2356) 0.2760 (0.0650 0.2356) 0.2828 (0.0458 0.1618)-1.0000 (0.0000 0.0000) 0.2935 (0.0179 0.0611)
MMU  0.0433 (0.0176 0.4062) 0.0665 (0.0550 0.8274) 0.0743 (0.0564 0.7587) 0.1618 (0.0946 0.5848) 0.0863 (0.0468 0.5417) 0.1170 (0.0552 0.4717) 0.1173 (0.0552 0.4707) 0.1383 (0.0649 0.4692) 0.1362 (0.0678 0.4976) 0.1383 (0.0649 0.4692) 0.1383 (0.0649 0.4692) 0.1006 (0.0441 0.4381) 0.0536 (0.0178 0.3327) 0.0631 (0.0179 0.2835) 0.0536 (0.0178 0.3327)
```

```
TREE #  1:  (16, (1, ((((13, 15), 14), 12), ((2, 3), (4, (5, (7, ((9, 6), (8, (10, 11)))))))))));   MP score: -1
This is a rooted tree.  Please check!
check convergence..
lnL(ntime: 30  np: 35):   -414.618395      +0.000000
  17..16   17..18   18..1    18..19   19..20   20..21   21..22   22..13   22..15   21..14   20..12   19..23   23..24   24..2    24..3    23..25   25..4    25..26   26..5    26..27   27..7    27..28   28..29   29..9    29..6
28..30   30..8    30..31   31..10   31..11
 0.104932 0.097778 0.100033 0.018530 0.032202 0.000004 0.040977 0.000004 0.000004 0.041150 0.187552 0.225321 0.069806 0.018542 0.019550 0.000004 0.111088 0.057084 0.063566 0.040867 0.000004 0.040059 0.000004 0.000004 0.018840
0.000004 0.000004 0.000004 0.000004 0.000004 2.491783 0.331447 0.000000 0.000001 1.740981

Note: Branch length is defined as number of nucleotide substitutions per codon (not per neucleotide site).

tree length =   1.28792

(16: 0.104932, (1: 0.100033, ((((13: 0.000004, 15: 0.000004): 0.040977, 14: 0.041150): 0.000004, 12: 0.187552): 0.032202, ((2: 0.018542, 3: 0.019550): 0.069806, (4: 0.111088, (5: 0.063566, (7: 0.000004, ((9: 0.000004, 6:
0.018840): 0.000004, (8: 0.000004, (10: 0.000004, 11: 0.000004): 0.000004): 0.000004): 0.040059): 0.040867): 0.057084): 0.000004): 0.225321): 0.018530): 0.097778);

(MMU: 0.104932, (SHI: 0.100033, ((((PSU: 0.000004, PCM: 0.000004): 0.040977, PRO: 0.041150): 0.000004, MAU: 0.187552): 0.032202, ((ASA: 0.018542, ATE: 0.019550): 0.069806, (CGL: 0.111088, (CNI: 0.063566, (MCA: 0.000004, ((MAG:
0.000004, MAR: 0.018840): 0.000004, (MGE: 0.000004, (PDU: 0.000004, PLU: 0.000004): 0.000004): 0.000004): 0.040059): 0.040867): 0.057084): 0.000004): 0.225321): 0.018530): 0.097778);

Detailed output identifying parameters

kappa (ts/tv) =  2.49178


dN/dS (w) for site classes (K=3)

p:   0.33145  0.00000  0.66855
w:   0.00000  1.00000  1.74098

dN & dS for each branch
```

branch	t	N	S	dN/dS	dN	dS	N*dN	S*dS
17..16	0.105	55.6	106.4	1.1639	0.0385	0.0331	2.1	3.5
17..18	0.098	55.6	106.4	1.1639	0.0359	0.0309	2.0	3.3
18..1	0.100	55.6	106.4	1.1639	0.0367	0.0316	2.0	3.4
18..19	0.019	55.6	106.4	1.1639	0.0068	0.0058	0.4	0.6
19..20	0.032	55.6	106.4	1.1639	0.0118	0.0102	0.7	1.1
20..21	0.000	55.6	106.4	1.1639	0.0000	0.0000	0.0	0.0
21..22	0.041	55.6	106.4	1.1639	0.0151	0.0129	0.8	1.4
22..13	0.000	55.6	106.4	1.1639	0.0000	0.0000	0.0	0.0
22..15	0.000	55.6	106.4	1.1639	0.0000	0.0000	0.0	0.0
21..14	0.041	55.6	106.4	1.1639	0.0151	0.0130	0.8	1.4
20..12	0.188	55.6	106.4	1.1639	0.0689	0.0592	3.8	6.3
19..23	0.225	55.6	106.4	1.1639	0.0828	0.0711	4.6	7.6
23..24	0.070	55.6	106.4	1.1639	0.0256	0.0220	1.4	2.3
24..2	0.019	55.6	106.4	1.1639	0.0068	0.0059	0.4	0.6
24..3	0.020	55.6	106.4	1.1639	0.0072	0.0062	0.4	0.7
23..25	0.000	55.6	106.4	1.1639	0.0000	0.0000	0.0	0.0
25..4	0.111	55.6	106.4	1.1639	0.0408	0.0351	2.3	3.7
25..26	0.057	55.6	106.4	1.1639	0.0210	0.0180	1.2	1.9
26..5	0.064	55.6	106.4	1.1639	0.0233	0.0201	1.3	2.1
26..27	0.041	55.6	106.4	1.1639	0.0150	0.0129	0.8	1.4
27..7	0.000	55.6	106.4	1.1639	0.0000	0.0000	0.0	0.0
27..28	0.040	55.6	106.4	1.1639	0.0147	0.0126	0.8	1.3
28..29	0.000	55.6	106.4	1.1639	0.0000	0.0000	0.0	0.0
29..9	0.000	55.6	106.4	1.1639	0.0000	0.0000	0.0	0.0
29..6	0.019	55.6	106.4	1.1639	0.0069	0.0059	0.4	0.6
28..30	0.000	55.6	106.4	1.1639	0.0000	0.0000	0.0	0.0
30..8	0.000	55.6	106.4	1.1639	0.0000	0.0000	0.0	0.0
30..31	0.000	55.6	106.4	1.1639	0.0000	0.0000	0.0	0.0
31..10	0.000	55.6	106.4	1.1639	0.0000	0.0000	0.0	0.0
31..11	0.000	55.6	106.4	1.1639	0.0000	0.0000	0.0	0.0

Naive Empirical Bayes (NEB) analysis
Positively selected sites (*: P>95%; **: P>99%)
(amino acids refer to 1st sequence: SHI)

		Pr(w>1)	post mean +- SE for w
1	S	0.633	1.103
3	R	1.000**	1.741
4	R	1.000**	1.741
5	L	0.607	1.056
6	H	1.000**	1.741
7	R	0.656	1.142
8	I	0.669	1.164
10	K	1.000**	1.741
11	R	0.630	1.097
13	R	0.657	1.144
14	S	0.669	1.164
16	R	0.593	1.033
17	R	0.589	1.025
18	R	0.660	1.148
19	R	0.646	1.125
20	R	0.662	1.152
21	H	1.000**	1.741

```
    22 S      0.546         0.951
    24 R      1.000**       1.741
    26 R      0.589         1.025
    27 R      0.593         1.033
    28 R      0.661         1.152
    29 H      0.506         0.881
    31 R      0.657         1.144
    33 C      1.000**       1.741
    34 K      1.000**       1.741
    35 R      0.662         1.152
    36 S      1.000**       1.741
    37 R      0.646         1.124
    38 -      0.665         1.157
    39 *      0.597         1.039
    40 R      0.589         1.025
    41 R      0.589         1.025
    42 R      0.662         1.152
    44 R      0.589         1.025
    46 R      0.592         1.031
    47 K      1.000**       1.741
    48 C      0.504         0.878
    49 R      0.589         1.025
    50 R      0.582         1.014
    51 H      1.000**       1.741
    52 H      1.000**       1.741
    53 H      1.000**       1.741
    54 -      0.655         1.141


Bayes Empirical Bayes (BEB) analysis (Yang, Wong & Nielsen 2005. Mol. Biol. Evol. 22:1107-1118)
Positively selected sites (*: P>95%; **: P>99%)
(amino acids refer to 1st sequence: SHI)

            Pr(w>1)     post mean +- SE for w

     6 H      0.601         2.249 +- 1.770
    52 H      0.632         2.397 +- 1.905



The grid (see ternary graph for p0-p1)

w0:   0.050  0.150  0.250  0.350  0.450  0.550  0.650  0.750  0.850  0.950
w2:   1.500  2.500  3.500  4.500  5.500  6.500  7.500  8.500  9.500 10.500


Posterior on the grid

w0:   0.099  0.096  0.093  0.092  0.093  0.096  0.100  0.105  0.110  0.113
w2:   0.300  0.255  0.148  0.091  0.062  0.045  0.034  0.026  0.021  0.018

Posterior for p0-p1 (see the ternary graph)

 0.025
 0.018 0.023 0.024
 0.012 0.016 0.019 0.023 0.021
 0.008 0.011 0.014 0.017 0.019 0.020 0.017
 0.006 0.008 0.010 0.013 0.014 0.017 0.017 0.017 0.013
```

```
 0.005 0.006 0.008 0.009 0.011 0.013 0.014 0.015 0.014 0.013 0.010
 0.004 0.005 0.006 0.007 0.009 0.010 0.011 0.012 0.012 0.013 0.011 0.010 0.008
 0.003 0.004 0.005 0.006 0.007 0.008 0.009 0.010 0.010 0.011 0.010 0.010 0.009 0.008 0.007
 0.003 0.004 0.005 0.005 0.006 0.007 0.008 0.009 0.009 0.009 0.009 0.009 0.008 0.008 0.008 0.007 0.006
 0.002 0.003 0.004 0.005 0.006 0.006 0.007 0.008 0.008 0.008 0.008 0.008 0.007 0.007 0.007 0.007 0.006 0.006 0.005

sum of density on p0-p1 =   1.000000

Time used:  1:52
```
